# Ionic Liquid‐Functionalized Periodic Mesoporous Organosilica: A Robust Support for Palladium Nanoparticles in Carbonylative Suzuki Coupling Reactions

**DOI:** 10.1002/asia.202401802

**Published:** 2025-03-19

**Authors:** Manan Sohanwal, Suheir Omar, Raed Abu‐Reziq

**Affiliations:** ^1^ Institute of Chemistry, Casali Center of Applied Chemistry, Center for Nanoscience and Nanotechnology the Hebrew University of Jerusalem 9190401 Israel

**Keywords:** periodic mesoporous organosilica, palladium nanoparticles, supported ionic liquid, carbonylative Suzuki reaction, carbonyl compounds

## Abstract

This study presents the synthesis and application of palladium nanoparticles (Pd NPs) supported on ionic‐liquid‐functionalized periodic mesoporous organosilica (PMO‐IL) as an efficient heterogeneous catalyst for carbonylative Suzuki coupling reactions. The PMO‐IL material was prepared via a sol‐gel polycondensation process using tetraethyl orthosilicate (TEOS) and a bis‐silylated ionic liquid monomer. The ionic liquid groups within the PMO framework facilitated the adsorption of palladium salts through ion exchange, followed by reduction to form Pd NPs. Comprehensive characterization of the Pd_(np)_@PMO‐IL system, including high‐resolution microscopy (HR‐SEM and HR‐TEM), X‐ray diffraction (XRD), solid‐state NMR, FT‐IR, and nitrogen adsorption‐desorption (BET) analyses, confirmed its structure, morphology, and high surface area. The catalytic system demonstrated remarkable activity, selectivity, and recyclability in the carbonylative Suzuki coupling reaction, achieving high turnover numbers (TON) and turnover frequencies (TOF) under mild conditions. This hybrid material highlights the potential of ionic liquid‐functionalized PMOs as versatile supports for metal nanoparticles in sustainable catalytic applications.

## Graphical abstract

An ionic liquid‐functionalized periodic mesoporous organosilica (PMO‐IL) is prepared via a sol‐gel method and serves as a support for palladium nanoparticles. The resulting Pd_(np)_@PMO‐IL catalyst exhibits high activity, selectivity, and recyclability in carbonylative Suzuki coupling reactions under mild conditions, providing an efficient and sustainable route to biaryl ketones.

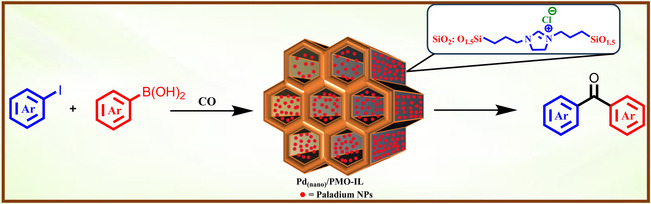

## 1 Introduction

In recent years, the discovery of periodic mesoporous organosilica (PMO) materials has marked a significant advancement across various fields.[^1^, ^2^, ^3^, ^4^] These materials exhibit immense potential in applications such as optical adsorbents, biological and medical agents, catalyst supports, and more.[^1^, ^5^, ^6^, ^7^, ^8^, ^9^, ^10^, ^11^, ^12^] Typically, PMOs are synthesized through sol‐gel hydrolysis and condensation processes involving bridged organosilica precursors and a structure‐directing agent. Their distinctive features, including well‐ordered mesoscopic channels with uniformly distributed organic functional groups, high surface area, excellent thermal stability, and customizable physicochemical properties, make them highly desirable for diverse biomedical and chemical applications.[^13^, ^14^, ^15^, ^16^]

A key advantage of PMOs lies in their hydrophobic organic groups embedded within the framework, which enhance the activity and stability of various guest molecules. One effective strategy for functionalizing PMO materials with organic spacers involves the use of ionic liquids (ILs). The integration of PMOs with ionic liquids has emerged as a promising method for creating novel materials with specialized functionalities, particularly in catalytic applications.[^17^, ^18^, ^19^, ^20^, ^21^, ^22^, ^23^, ^24^, ^25^, ^26^, ^27^, ^28^, ^29^, ^30^, ^31^, ^32^, ^33^, ^34^] Ionic liquids are commonly immobilized via covalent anchoring, polymerization, encapsulation, or sol‐gel condensation methods.[^35^, ^36^, ^37^, ^38^, ^39^, ^40^, ^41^, ^42^, ^43^, ^44^] Ionic liquids offer several advantageous properties, including low volatility, high thermal stability, and the ability to uniformly distribute on solid surfaces. These characteristics, combined with their ability to immobilize and stabilize metal nanoparticles (NPs),[^45^, ^46^, ^47^] have driven growing interest in using hybrid systems of ILs and PMOs for catalytic purposes.[^48^, ^49^] Immobilizing metal NPs on solid supports such as organic, inorganic, or hybrid materials addresses key challenges like recovery and agglomeration, making these systems highly efficient for various catalytic processes.[^50^, ^51^, ^52^, ^53^, ^54^, ^55^, ^56^, ^57^, ^58^]

The carbonylative Suzuki coupling reaction has emerged as a powerful tool in organic synthesis, enabling the construction of carbon‐carbon bonds with the concurrent incorporation of a carbonyl group.^[59]^ This process facilitates the direct and efficient synthesis of biaryl ketones from carbon monoxide, aryl halides, and aryl boronic acids via simple and atom‐economical transformations. Conventionally, such ketones are produced via Friedel‐Crafts acylation, a method unsuitable for industrial applications due to its inherent drawbacks.[^60^, ^61^] The Carbonylative Suzuki coupling, primarily utilizing palladium‐based homogeneous catalysts, has been the subject of extensive research.^[59]^ However, homogeneous systems pose significant challenges in catalyst recovery and reuse, limiting their practical application. To address these challenges, heterogeneous catalytic systems have been developed to facilitate catalyst separation and improve reusability.[^62^, ^63^] In this work, we present carbonylative Suzuki coupling reactions utilizing a heterogeneous system comprising palladium nanoparticles supported on PMO functionalized with ionic liquid groups (PMO‐IL). Notably, the application of PMO‐IL systems in carbonylative Suzuki coupling reactions has not been reported to date. The catalytic system was synthesized via a sol‐gel process, wherein an ionic liquid‐based silane monomer was co‐polymerized with tetraethoxysilane in the presence of a Pluronic P123 co‐polymer under acidic conditions. This approach yielded a highly ordered 2D‐hexagonal mesostructure in the PMO‐IL bulk material. Following the development of the organic‐silica hybrid framework, palladium nanoparticles were successfully incorporated into the mesopores of the PMO‐IL structure. The resulting catalytic material exhibited excellent performance in carbonylative Suzuki coupling reactions, demonstrating both high reactivity and selectivity.

## Experimental Section

### General Information

X‐ray powder diffraction (XRD) patterns were obtained on a D8 advanced diffractometer (Bruker AXS, Karlsruhe, Germany) with Cukα radiation. The infrared spectra were recorded using a Perkin Elmer (FTIR 65) spectrometer. Scanning electron microscopy (SEM) was performed using a Sirion SEM microscope (FEI Company), a Shottky‐type emission source, and a secondary electron (SE) detector, operated at a voltage of 5 kV. Transmission electron microscopy (TEM), scanning transmission electron microscopy (STEM), and electron diffraction spectroscopy (EDS) were performed with (S)TEM Tecnai F20 G2 (FEI Company, USA) operated at 200 kV. Thermogravimetric analysis (TGA) was performed on a Mettler Toledo TG 50 analyzer. Measurements were carried out at a temperature range that extended from 25 to 900 °C and at a heating rate of 10 °C /min under an inert atmosphere (N_2_). The specific surface areas were calculated by using the Brunauer‐Emmett‐Teller (BET) equation and utilizing a high‐speed gas sorption analyzer, Quantachrome Nova 1200e instrument. A gas chromatography (GC) instrument (Aglient Technologies, 7890A) with a capillary column (HP‐5, 30 meters) was used to determine the reactions’ conversion and selectivity. ^1^H NMR and ^13^C NMR spectra were recorded using a Bruker DRX‐400 and DRX‐500 instrument. Inductively coupled plasma mass spectrometry measurements (ICP) were performed on a 7500cx (Agilent company) instrument using external standard calibration for determining the palladium loadings.

### Synthesis Of 1,3‐Bis(3‐(Triethoxysilyl)Propyl)‐4,5‐Dihydro‐1H‐Imidazol‐3‐Ium Chloride (Bridged‐IL)

(3‐Chloropropyl)triethoxysilane (4.3 g, 18 mmol) and 1‐(3‐(triethoxysilyl)propyl)‐4,5‐dihydro‐1*H*‐imidazole (5 g, 18 mmol) were heated under nitrogen at 120 °C. The progress of the reaction was monitored by ^1^H NMR; the reaction was completed after four days. The mixture was cooled down to room temperature and a brown‐yellowish viscous liquid (8.9 g) was obtained (96 % yield). ^1^H NMR (400 MHz, CDCl_3_) δ ppm: 0.57–0.669 (m, 4H), 1.128 (t, J=7.2 Hz, 18H), 1.716–1.793 (m, 4H), 3.564 (t, J=7.2 Hz, 4H), 3.318 (q, J=7.2 Hz, 12H), 3.916 (s, 4H), 10.05 (s, 1H). ^13^C NMR (100 MHz, CDCl_3_) δ ppm: 7.08, 17.96, 20.85, 47.94, 50.04, 56.99, 58.14, 158.29. IR: 3382, 2966, 2911, 2843, 1652 cm‐1. Anal. calcd. for C_21_H_47_ClN_2_O_6_Si_2_: C 48.95, H 9.19, Cl 6.88, N 5.44; Found: C 46.70, H 9.23, Cl 6.56, N 5.02.

### Synthesis Of Ionic Liquid Functionalized Periodic Mesoporous Organosilica (PMO‐IL)^[64]^

The PMO‐IL bulk system was prepared by hydrolysis and co‐condensation of tetraethyl orthosilicate (TEOS) and 1,3‐bis(3‐(triethoxysilyl)propyl)‐4,5‐dihydro‐1*H*‐imidazol‐3‐ium chloride (**Bridged‐IL**) in the presence of Pluronic P123 under acidic conditions. Typically, Pluronic P123 (1.67 g) was added to a solution containing 10.5 g of deionized water, 46 mL of HCl (2 M), and 8.8 g of potassium chloride. The obtained mixture was then stirred at 40 °C. After a clear solution was achieved, a mixture of tetraethoxysilane (6.5 mmol) and **Bridged‐IL** (0.89 mmol) was added dropwise to the reaction vessel and stirred at the same temperature for 24 hr. Later, the obtained mixture was aged for three days in a Teflon vessel at 100 °C. The solid product was washed in a Soxhlet apparatus using acetone for 24 hours to remove the surfactant and then with ethanol for extra 24 hr. The final PMO‐IL bulk material was dried at 54 °C for 16 hours to afford a fine white‐beige powder.

### Supporting Palladium Nanoparticles On The PMO‐IL Bulk Material (Pd_(Np)_@PMO‐IL)

First, 0.5 g of a solid PMO‐IL sample was dispersed in 30 mL of methanol. To this mixture, 73 mg (0.25 mmol) of Na_2_PdCl_4_, dissolved in 30 mL of methanol, were added dropwise and the resulting mixture was sonicated for 30 minutes and stirred for 12 h at room temperature. Then, 38 mg (1 mmol) of sodium borohydride were added, and the mixture was stirred for 24 hours at room temperature. The catalytic Pd_(np)_@PMO‐IL system was centrifuged and washed three times with methanol and then dried at 54 °C for 16 hours. The palladium loading was 0.52 mmolg^−1^, as determined by ICP analysis.

### Synthesis Of PMO Functionalized With Phenylene (PMO‐Ph) And Ethylene (PMO‐Et) Groups

The PMO systems functionalized with either phenylene (PMO‐Ph) or ethylene (PMO‐Et) groups were synthesized via hydrolysis and co‐condensation of TEOS with the respective bridged organosilane precursor, using Pluronic P123 as a structure‐directing agent under acidic conditions.

In a typical procedure, 1.67 g of Pluronic P123 was dissolved in a solution containing 10.5 g of deionized water, 46 mL of hydrochloric acid (HCl, 2M), and 8.8 g of potassium chloride. The mixture was stirred at 40 °C until a clear solution was obtained. Subsequently, a mixture of TEOS (6.5 mmol) and either bis(triethoxysilyl)benzene (0.89 mmol) for PMO‐Ph or bis(triethoxysilyl)ethane (0.89 mmol) for PMO‐Et was added dropwise, followed by continuous stirring for 24 hours.

The resulting mixture was then transferred to a Teflon vessel and aged at 100 °C for three days to enhance structural development. The obtained solid product was purified using a Soxhlet apparatus, first with acetone for 24 hours, followed by ethanol for another 24 hours, ensuring the removal of residual surfactant. Finally, the PMO materials were dried at 54 °C for 16 hours, yielding a fine white‐beige powder, confirming successful synthesis of both PMO‐Ph and PMO‐Et.

### Incorporation Of Palladium Nanoparticles Into PMO‐Ph And PMO‐Et

0.5 g of the solid PMO‐Ph or PMO‐Et material was dispersed in 30 mL of methanol under continuous stirring. A solution of Na_2_PdCl_4_ (73 mg, 0.25 mmol) in 30 mL of methanol was then added dropwise to the dispersion. The mixture was sonicated for 30 minutes, followed by stirring at room temperature for 12 hours to facilitate the adsorption of palladium ions onto the PMO surface. Then, sodium borohydride (38 mg, 1 mmol) was introduced into the reaction mixture, and stirring was continued for an additional 24 hours at room temperature. The resulting materials were then separated by centrifugation, washed three times with methanol to remove residual impurities, and dried at 54 °C for 16 hours.

### General Procedure For The Carbonylative Suzuki Coupling Reaction

The appropriate amounts of iodoarene (0.26 mmol) and K_2_CO_3_ (0.78 mmol), along with phenylboronic acid (0.29 mmol), were added to a 4 mL solvent containing the supported Pd‐catalyst (5 mg, 0.0026 mmol Pd). The mixture was placed in a 25 mL glass‐lined autoclave; the autoclave was sealed, purged three times with carbon monoxide, and pressurized with 100 psi of carbon monoxide. The reaction was heated at 80 or 100 °C. After 14 hours the autoclave was cooled to room temperature and the carbon monoxide was carefully released. The catalyst was filtered and the solution was concentrated. The product was then extracted with ether and washed with water, dried over magnesium sulfate, and then concentrated under reduced pressure. The crude products were analyzed by GC and ^1^H NMR. Purified products were characterized using ^1^H NMR and ^13^C NMR spectroscopy.

## 2 Results and Discussion

### 2.1 Preparation Of A Catalytic Ionic Liquid‐Modified Periodic Mesoporous Organosilica Bulk System (Pd_(Np)_@PMO‐IL)

**Scheme 1:**
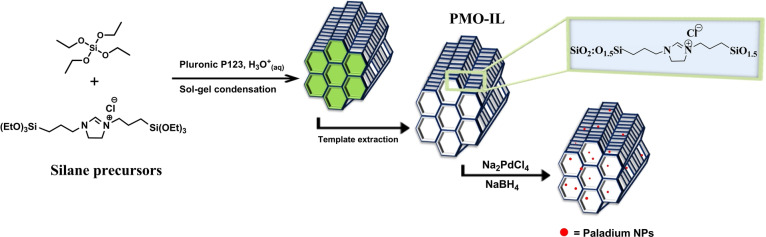
Illustration of the preparation of the Pd_(np)_@PMO‐IL catalytic system.

The Bridged‐IL was synthesized via a solvent‐free substitution reaction between (3‐chloropropyl)triethoxysilane and 1‐(3‐(triethoxysilyl)propyl)‐4,5‐dihydro‐*1H*‐imidazole at 120 °C. Subsequently, the PMO‐IL bulk material was prepared through a conventional sol‐gel condensation reaction of tetraethoxysilane (TEOS) and Bridged‐IL under acidic conditions, using Pluronic P123 as a structure‐directing agent. The ionic liquid groups incorporated into the PMO framework facilitated the adsorption of palladium salt through an ion‐exchange process. This was achieved by stirring the PMO‐IL material with a methanolic solution of Na_2_PdCl_4_ for 24 hours. Palladium nanoparticles were then easily formed by reducing the adsorbed palladium salt with an excess of sodium borohydride, as illustrated in Scheme 1.

### 2.2 Characterization Of Pd_(Np)_@PMO‐IL

**Figure 1:**
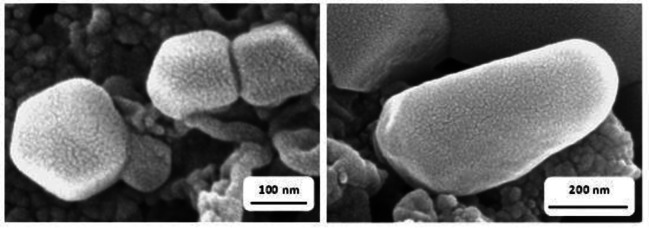
SEM micrographs of PMO‐IL system.

Various characterization techniques were utilized to analyze the structural features of the PMO‐IL system. Scanning Electron Microscopy (SEM) was employed to examine the morphological properties of the material (Figure 1). The PMO‐IL system displayed irregular morphologies with no distinct shapes, featuring particles of varying dimensions, with widths ranging from 100 nm to 500 nm.

**Figure 2:**
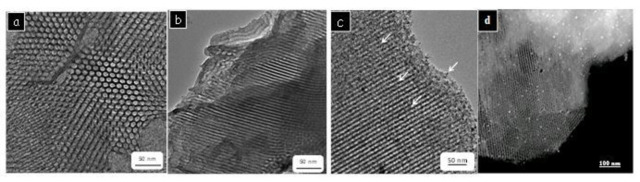
S/TEM micrographs of PMO‐IL: (a) and (b); Pd_(np)_@PMO‐IL: (c) and (d).

Scanning/transmission electron microscopy (S/TEM) provided further insight into the structural organization of the PMO‐IL system, revealing the presence of 2D hexagonally ordered mesostructures. These structures exhibited both honeycomb‐like arrangements and tubular mesochannels (Figures 2a, 2b). Moreover, TEM and STEM analyses of Pd_(np)_@PMO‐IL confirmed the successful formation of palladium nanoparticles and their uniform deposition within the mesoscopic channels of the PMO‐IL system (Figures 2c, 2d).

**Figure 3:**
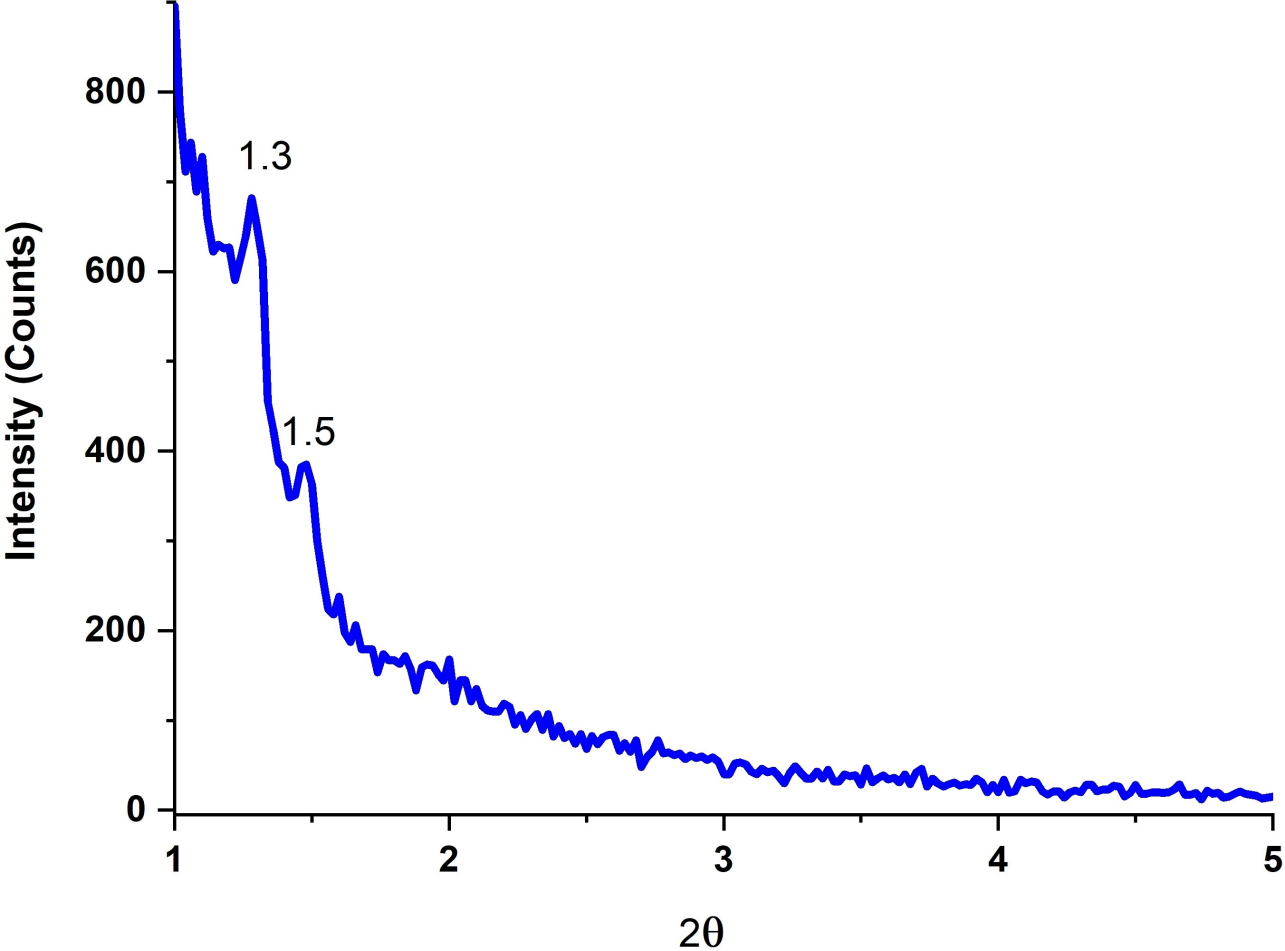
XRD pattern of PMO‐IL.

While the TEM images demonstrated a highly ordered structure, the XRD pattern of the PMO‐IL exhibited two weak diffraction peaks at 2θ=1.3° and 2θ=1.5° (Figure 3). These peaks are attributed to the periodic arrangement of the mesoporous organosilica framework.

**Figure 4:**
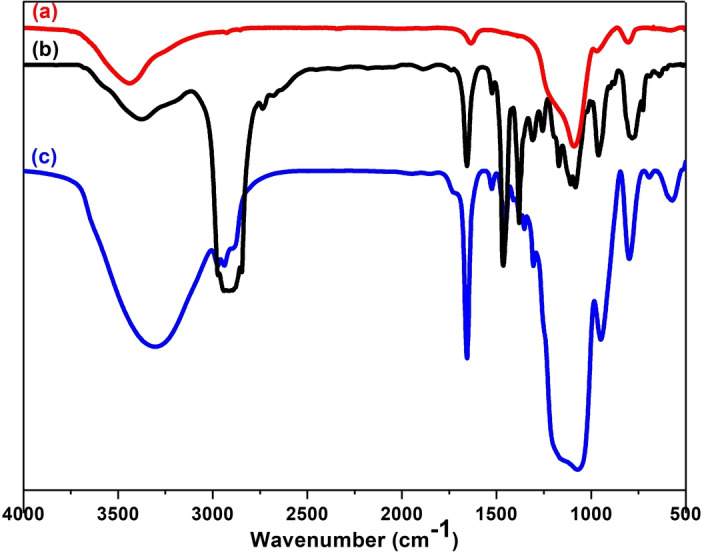
IR spectra of (a) silica bulk material, (b) Bridged‐IL, and (c) PMO‐IL.

The framework of the PMO‐IL was analyzed using FT‐IR spectroscopy (Figure 4). The IR spectrum of PMO‐IL (Figure 4, curve c) was compared with that of the synthesized Bridged‐IL (Figure 4, curve b) and a control bulk system prepared solely from TEOS (Figure 4, curve a) to confirm the incorporation of ionic liquid groups into the PMO material. Absorption bands observed at 1160 cm^−1^ and within the range of 3100–3750 cm^−1^ in curves a and c correspond to the asymmetric stretching vibrations of siloxane groups (Si−O−Si) and the stretching vibrations of Si−OH groups, respectively. The sharp peak at 1656 cm^−1^, present in curve c, confirmed the incorporation of the imidazolium group into the PMO‐IL system, as this frequency is characteristic of the stretching vibrations of C=N bonds associated with the Bridged‐IL (curves b and c).

**Figure 5:**
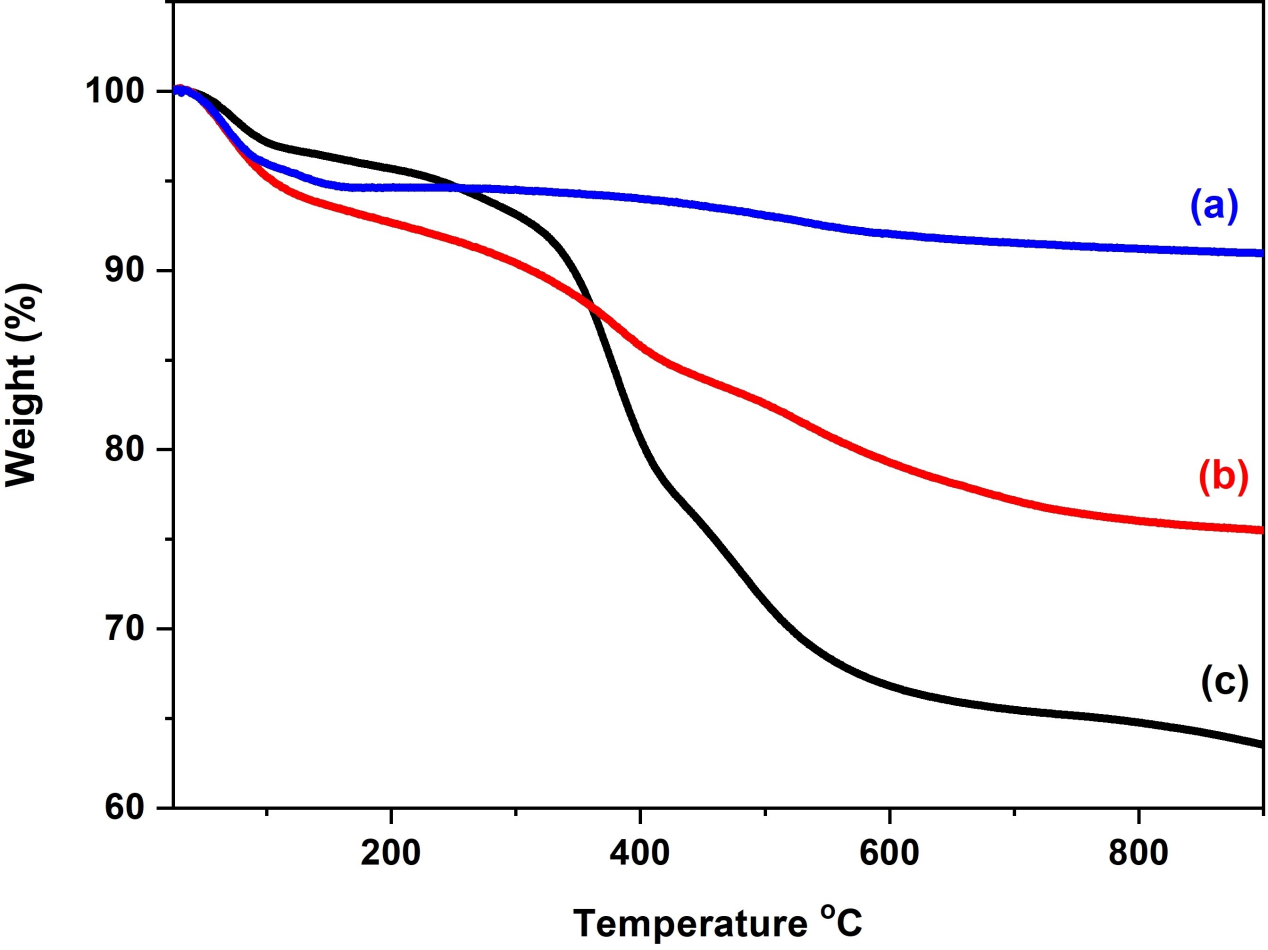
TGA curves of (a) bare silica bulk material; (b) PMO‐IL, and (c) Pd_(np)_@PMO‐IL.

Thermogravimetric analysis (TGA) was performed to confirm the presence of ionic liquid groups in the hybrid PMO‐IL system and to quantify their content (Figure 5). The results revealed an increase in organic content after the incorporation of Bridged‐IL into the silica framework and further after the formation of palladium nanoparticles (NPs). The bare silica material showed a minimal weight loss of 8.7 %, attributed to residual solvents and unreacted species (Figure 5, curve a). In contrast, the PMO‐IL system exhibited a total weight loss of approximately 18 %, while the Pd_(np)_@PMO‐IL system showed a weight loss of about 24 %. These losses, occurring predominantly in the temperature range of 200–600 °C, are associated with the decomposition of ionic liquid groups (Figure 5, curves b and c).

**Figure 6:**
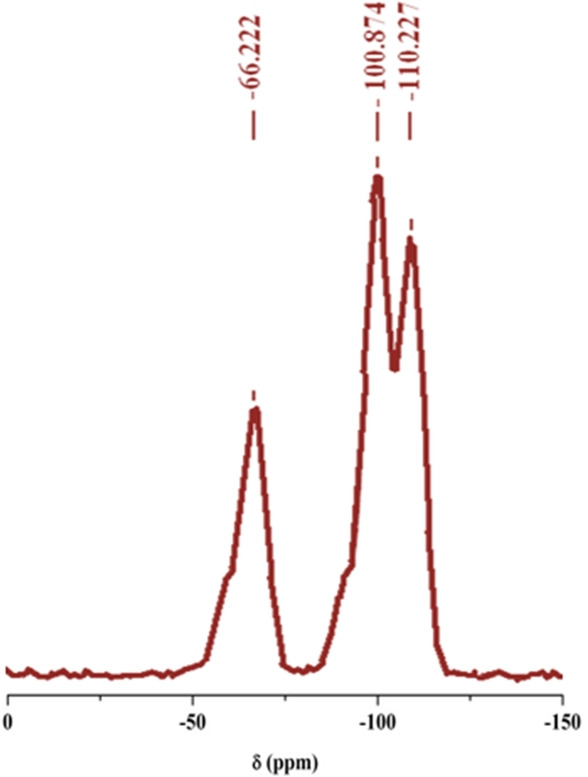
^29^Si CP‐MAS NMR spectroscopy.

Solid‐state ^29^Si CP‐MAS NMR spectroscopy provided additional evidence for the incorporation of the imidazolium group into the silica framework. As shown in Figure 6, two upfield resonance peaks were observed, corresponding to the native silica absorptions of siloxane groups (Q^4^, δ=−110.22 ppm) and single silanol groups (Q^3^, δ=−100.87 ppm). A distinct chemical shift at δ= −66.22 ppm was assigned to T^3^ organosilica species. These results confirm that Q^4^, Q^3^, and T^3^ are the primary microstructural components forming the network structure of the PMO‐IL bulk material.

**Figure 7:**
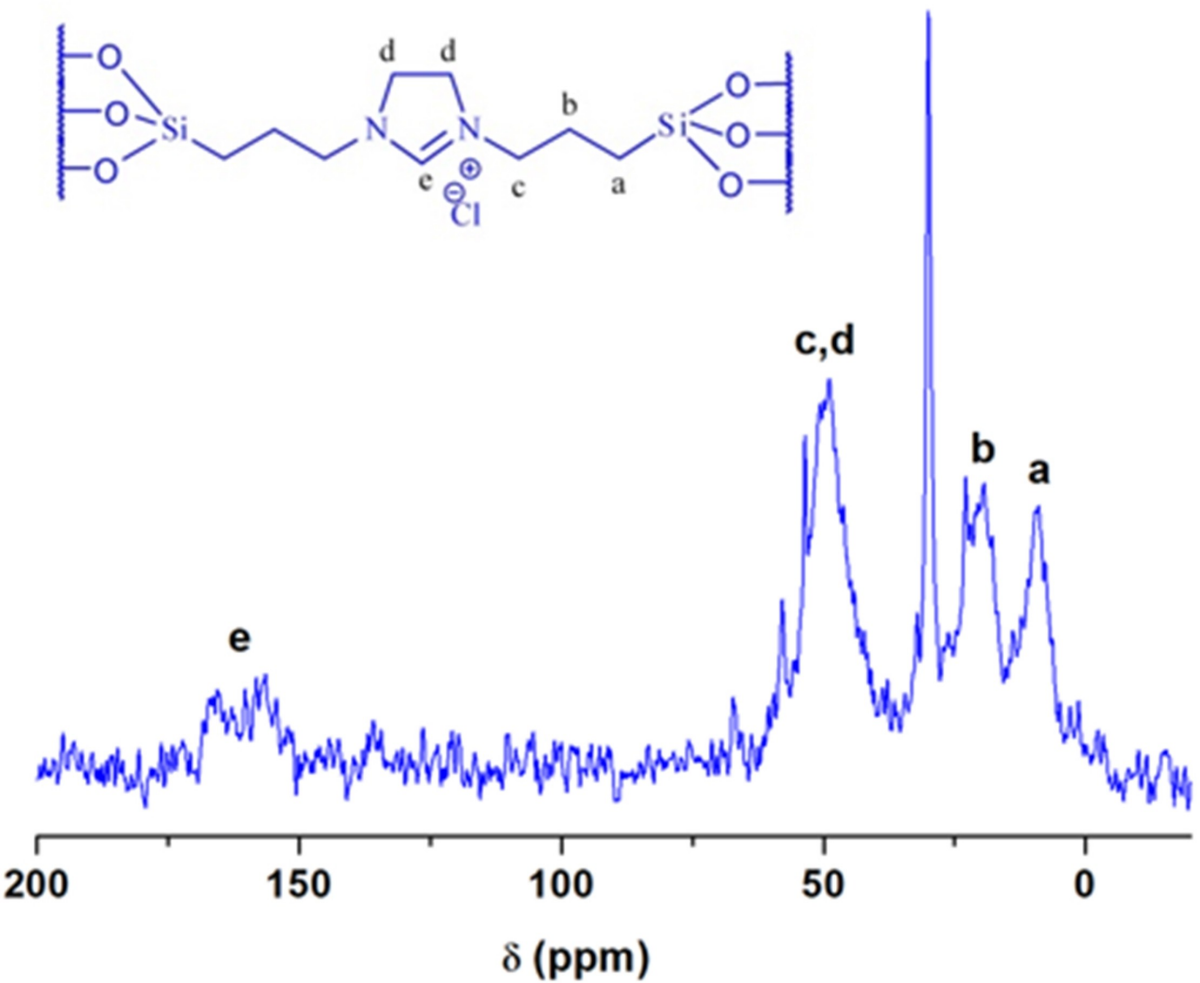
^13^C CP‐MAS NMR spectroscopy.

To further confirm the presence of the ionic liquid group, ^13^C CP‐MAS NMR spectroscopy was performed. The ^13^C CP‐MAS NMR spectrum (Figure 7) displayed absorption peaks in the range of 0–160 ppm, which are characteristic of the carbon species in the ionic liquid groups. The peaks were assigned as follows: δ (ppm)=9.1 (Si**C**H_2_), 22.3 (SiCH_2_ **C**H_2_CH_2_N), 49.7 (SiCH_2_CH_2_ **C**H_2_N, N**C**H_2_ **C**H_2_N), and 159.2 (N**C**HN). This analysis conclusively confirmed the successful incorporation of the ionic liquid groups into the silica framework.

**Figure 8:**
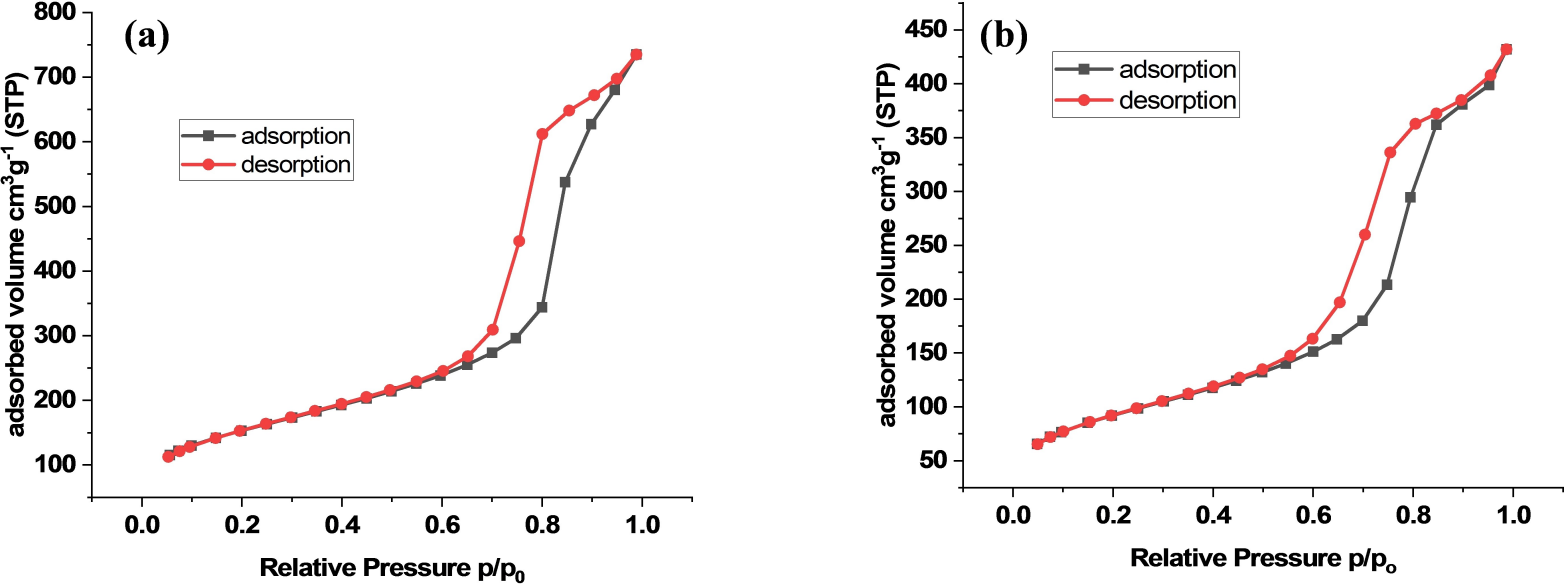
N_2_ adsorption‐desorption isotherms of (a) PMO‐IL and (b) Pd_(np)_@PMO‐IL.

The N_2_ adsorption‐desorption isotherms of the PMO‐IL and Pd_(np)_@PMO‐IL systems were analyzed using Brunauer–Emmett–Teller (BET) methodology to assess their porosity and surface properties. The BET isotherm for the PMO‐IL system exhibited a type IV hysteresis loop, characteristic of mesoporous materials (Figure 8a). This system demonstrated a surface area of 541 m^2^/g and an average pore size of 3.7 nm. For the Pd_(np)_@PMO‐IL system, the isotherm also showed a type IV hysteresis loop (Figure 8b); however, the surface area decreased to 329 m^2^/g, and the average pore size reduced to 2.8 nm. This reduction in surface area and pore size can be attributed to the incorporation of palladium nanoparticles within the mesoporous framework, which partially occupies the pore spaces and alters the material‘s structural characteristics. These results confirm that the palladium nanoparticles are effectively embedded within the PMO‐IL system while preserving the mesoporous nature of the material.

**Figure 9:**
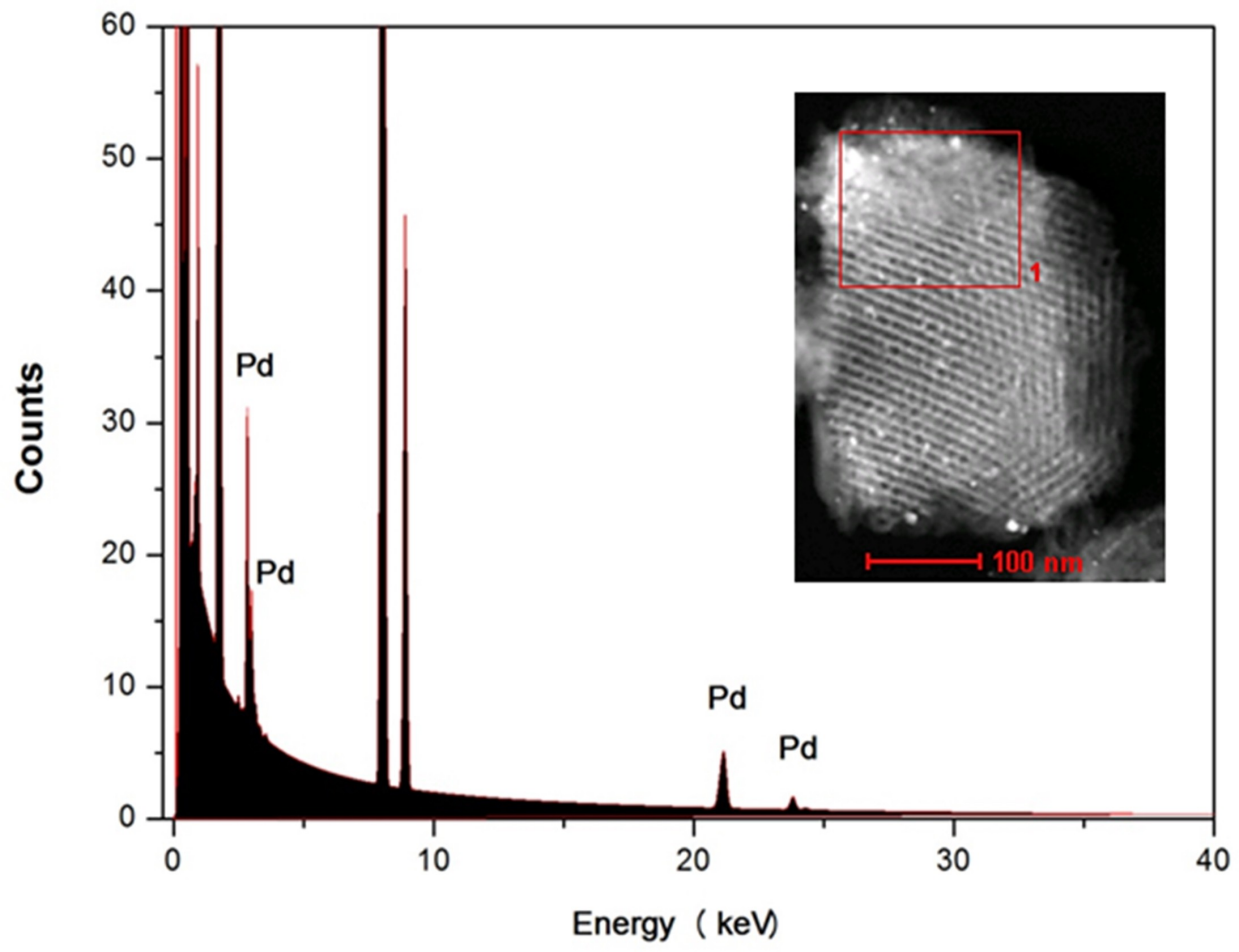
EDX spectroscopy of the Pd_(np)_@PMO‐IL system.

**Figure 10:**
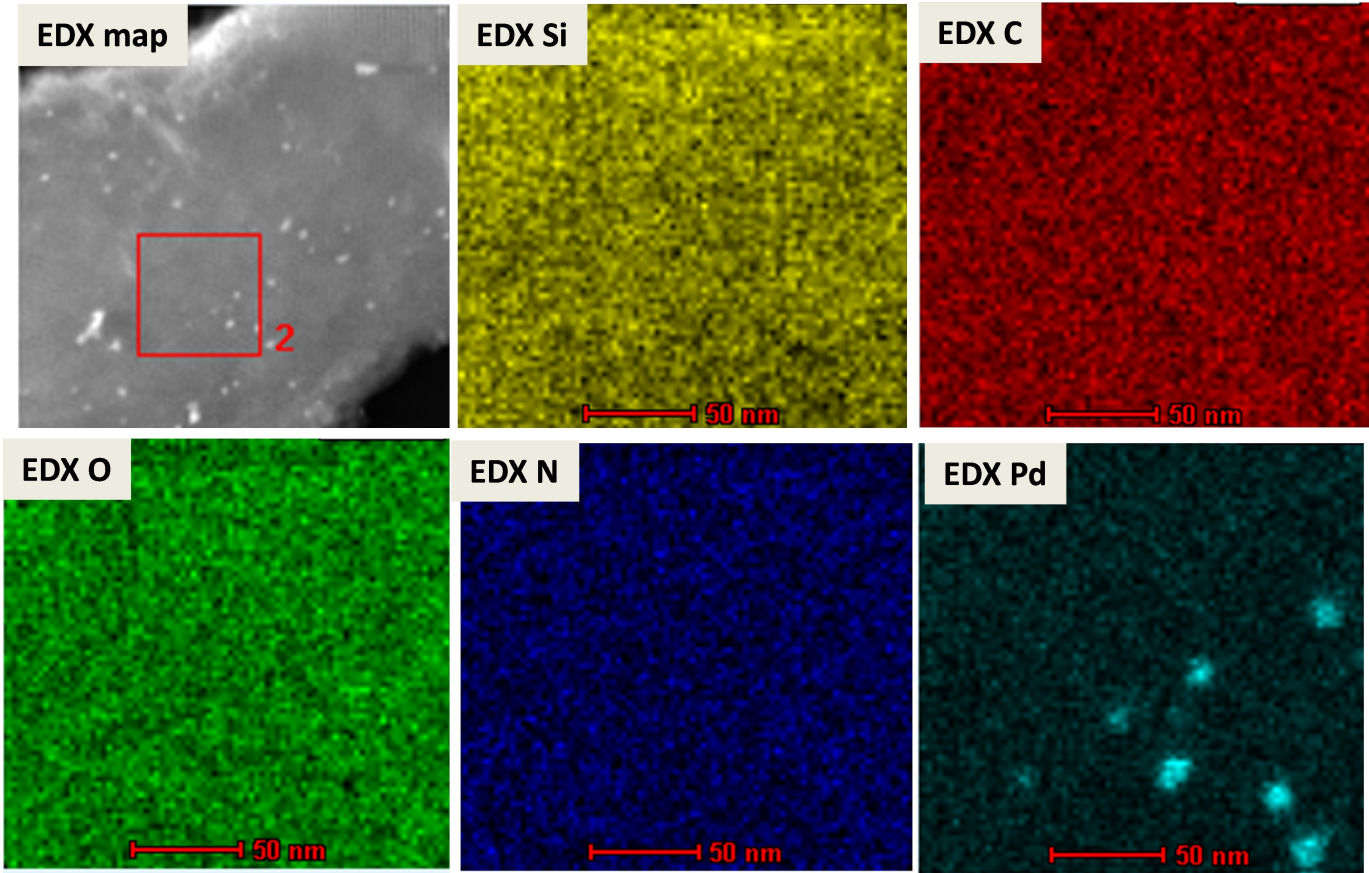
EDX mapping analysis of the Pd_(np)_@PMO‐IL system.

The chemical composition of the resulting Pd_(np)_@PMO‐IL system was further analyzed using Energy‐Dispersive X‐ray (EDX) spectroscopy (Figure 9) and EDX mapping analysis (Figure 10). EDX spectroscopy confirmed the presence of palladium nanoparticles, providing direct evidence of their incorporation into the system. Complementary EDX mapping analysis offered a detailed visualization of the elemental distribution within the material. The mapping results revealed the uniform presence of nitrogen, silicon, oxygen, and carbon, indicating their homogeneous integration throughout the PMO‐IL framework. In contrast, palladium was detected at a lower density, specifically localized within the mesopores. This pattern clearly demonstrates the successful deposition of palladium nanoparticles within the mesostructured channels of the Pd_(np)_@PMO‐IL system, corroborating the uniform incorporation of ionic liquid groups and the targeted loading of palladium nanoparticles.

To explore the role of ionic liquid groups in the formation and stabilization of palladium nanoparticles, we conducted comparative studies by synthesizing two additional types of PMO using TEOS and either 1,4‐bis(triethoxysilyl)benzene or 1,2‐bis(triethoxysilyl)ethane in a molar ratio of 7.3 : 1. This ratio was chosen to match that used in the preparation of PMO‐IL, yielding PMO‐Ph and PMO‐Et, respectively.

We then attempted to incorporate palladium nanoparticles within their frameworks using the same procedure applied for the Pd_(np)_@PMO‐IL system. However, at the end of the synthesis, we observed that black palladium precipitated separately from the white suspension of the PMO particles, indicating unsuccessful incorporation of palladium into the PMO structures (Supporting Information, Figure S1).

To confirm this result, we conducted SEM and TEM analyses. SEM images revealed that both PMO‐Ph and PMO‐Et formed irregularly shaped particles without a defined morphology (Supporting Information, Figure S2a for PMO‐Ph and Figure S3a for PMO‐Et). Furthermore, TEM analysis confirmed the absence of palladium nanoparticles within the frameworks of both materials (Supporting Information, Figures S2b and S3b).

BET surface area analysis further supported these findings. PMO‐Ph exhibited an initial surface area of 597.8 m^2^/g with an average pore size of 4.6 nm (Supporting Information, Figure S4a). After attempting to incorporate palladium, the surface area slightly increased to 659 m^2^/g with an average pore size of 4.7 nm (Supporting Information, Figure S4b). Similarly, PMO‐Et initially had a surface area of 336.8 m^2^/g and an average pore size of 4.8 nm (Supporting Information, Figure S5a), which changed to 309.9 m^2^/g and 4.7 nm, respectively, after the incorporation process (Supporting Information, Figure S5b). These results confirmed that palladium nanoparticles were not successfully formed within the channels of either PMO‐Ph or PMO‐Et.

This highlights the crucial role of IL groups in PMO‐IL, which facilitate palladium salt adsorption through ion exchange and stabilize palladium nanoparticles within the porous framework. Previous studies have reported that IL groups play a significant role in metal nanoparticle stabilization, primarily through the formation of N‐heterocyclic carbene (NHC)–metal interactions.^[65]^

### 2.3 Catalysis

The catalytic activity of the Pd_(np)_@PMO‐IL system was evaluated using the heterogeneous carbonylative Suzuki coupling reaction of iodoarenes with phenylboronic acid. The reactions were conducted under different solvent conditions at 80 °C, using potassium carbonate as a base, 100 psi of carbon monoxide (CO), and 1 mol% palladium catalyst with a variety of aryl halides. The results, summarized in Table 1, demonstrate the high catalytic efficiency of the system, particularly with iodoarenes as substrates.

**Table 1:** Carbonylative Suzuki coupling reaction catalyzed by Pd_(np)_@PMO‐IL.^[a]^

Entry	Substrate	Solvent	Conversion (%)^[b]^	Selectivity (%)^[b]^	TON	TOF (h^−1^)
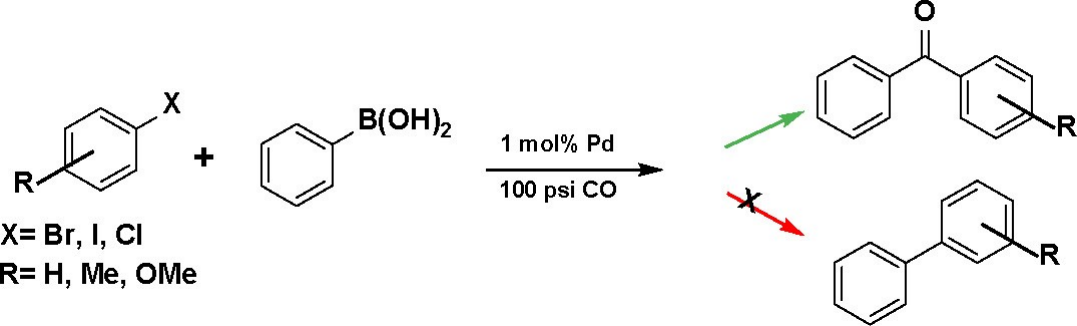						
**1**	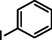	Toluene	84	98	84	6
**2**	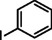	Anisole	99	>99	99	7.07
**3**	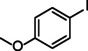	Toluene	100	88	100	7.14
**4**.	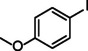	Anisole	>99	100	99	7.07
**5**	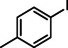	Toluene	41	96	41	2.9
**6**	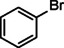	Anisole	5	12	5	0.35
**7**	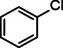	Anisole	–	–	–	–
**8**	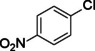	Anisole	–	–	–	–
**9^[c]^**	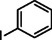	Anisole	100	100	1000	71.4
**10^[d]^**	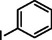	Anisole	72	97	720	720

[a] Reaction conditions: 1 mol% catalyst, 1 mmol substrate, 1.1 mmol phenylboronic acid, 3 mmol K_2_CO_3_, 4 mL solvent, 100 psi of CO, 80 °C, 14 hrs.; [b] Determined by ^1^H NMR and GC; [c] 0.1 mol% catalyst; [d] 0.1 mol% catalyst, 1 h.

Iodobenzene proved to be an excellent substrate, achieving efficient coupling when anisole was used as the solvent (Table 1, entry 2). In contrast, bromobenzene showed significantly lower reactivity, with very poor conversion under identical conditions (Table 1, entry 6), and chlorobenzene exhibited no reactivity at all (Table 1, entry 7). Similarly, a substrate with a strong electron‐withdrawing group, such as 1‐chloro‐4‐nitrobenzene, failed to undergo any reaction (Table 1, entry 8). Anisole outperformed toluene as a solvent; for example, iodobenzene achieved only 84 % conversion in toluene (Table 1, entry 2). Similar trends were observed with 4‐iodoanisole, which displayed complete conversion with high selectivity in anisole, while moderate selectivity was observed in toluene (Table 1, entries 3 and 4). A significantly lower conversion (41 %) was noted when 4‐iodotoluene was used in toluene (Table 1, entry 5).

Additionally, the catalyst exhibited remarkable performance at higher substrate‐to‐catalyst ratios and shorter reaction times. When the substrate‐to‐catalyst ratio was increased to 1000 : 1, very high turnover numbers (TON) were achieved (Table 1, entry 9). Furthermore, excellent turnover frequencies (TOF) were observed when the reaction time was reduced to just one hour (Table 1, entry 10). These results highlight the robustness and efficiency of the Pd_(np)_@PMO‐IL system for carbonylative Suzuki coupling reactions.

Furthermore, the PMO‐Ph and PMO‐Et systems, after palladium incorporation, showed no catalytic activity in the carbonylative Suzuki coupling of iodobenzene with phenylboronic acid, as no products were detected.

To assess its versatility, the Pd_(np)_@PMO‐IL catalyst was evaluated in carbonylative Suzuki coupling reactions using a variety of substituted iodoarenes and phenylboronic acids featuring both electron‐donating groups (EDGs) and electron‐withdrawing groups (EWGs). The results, summarized in Table 2, demonstrate the catalyst‘s high efficiency and selectivity, providing moderate to excellent isolated yields of aryl ketones under optimized conditions.

**Table 2:** Carbonylative Suzuki coupling reaction catalyzed by Pd_(np)_@PMO‐IL.^[a]^

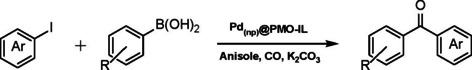				
Entry	Arylboronic Acid	Aryl Iodide	Product	Isolated yield (%)
**1**	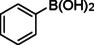	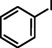	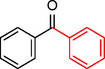	86
**2**	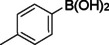	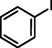	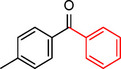	84
**3**	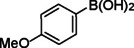	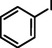	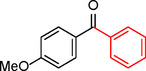	85
**4**	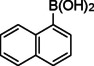	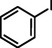	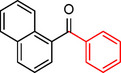	76
**5**	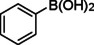	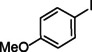	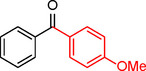	82
**6**	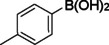	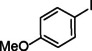	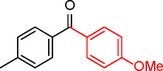	84
**7**	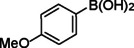	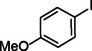	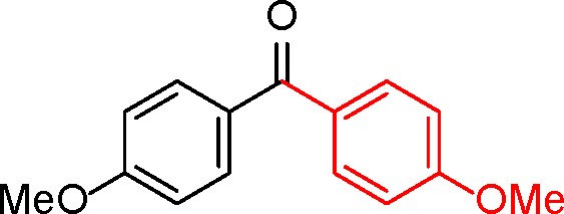	86
**8**	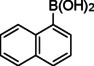	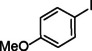	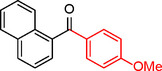	80
**9**	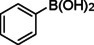	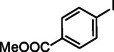	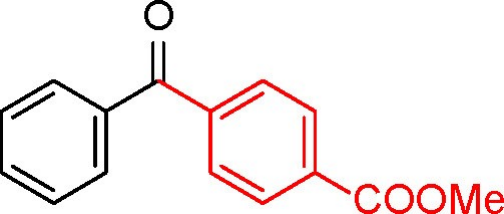	79
**10**	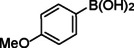	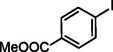	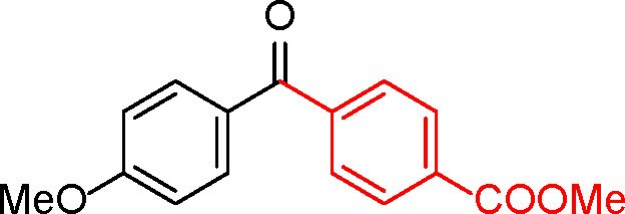	78
**11**	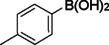	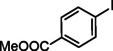	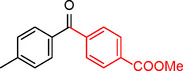	74
**12^[b]^**	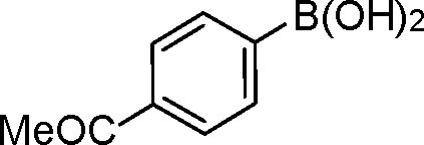	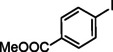	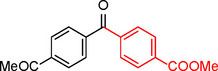	60
**13**	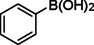	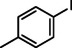	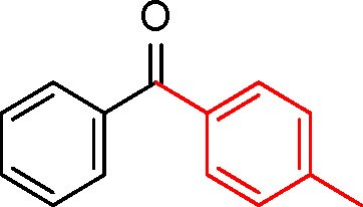	82
**14**	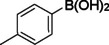	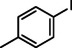	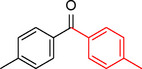	84
**15**	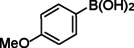	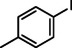	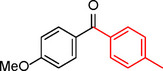	86
**16**	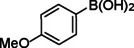	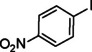	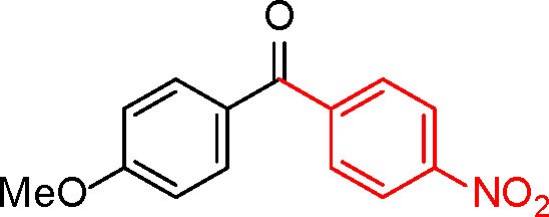	78
**17^[b]^**	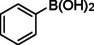	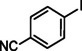	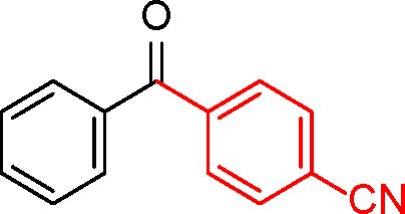	73
**18^[b]^**	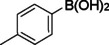	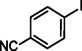	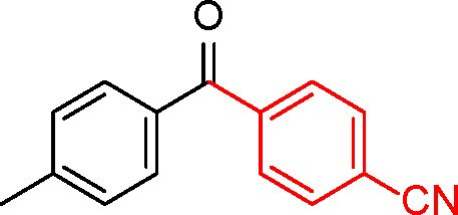	75
**19^[b]^**	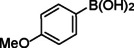	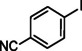	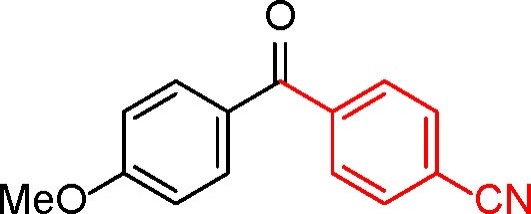	77
**20^[b]^**	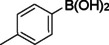	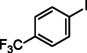	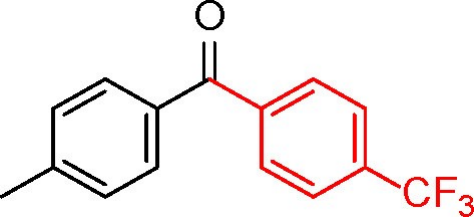	74
**21^[b]^**	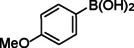	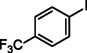	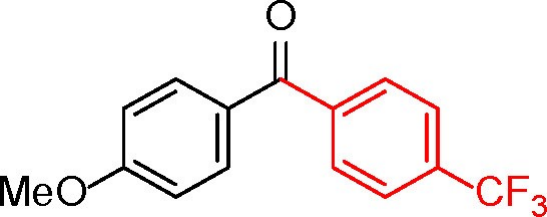	61
**22^[b]^**	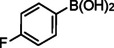	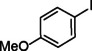	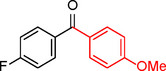	63
**23^[b]^**	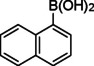	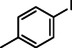	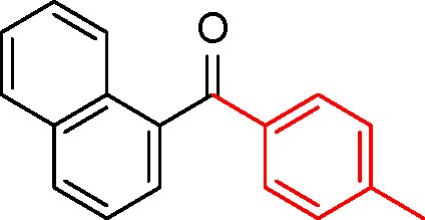	62
**24^[b]^**	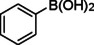	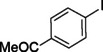	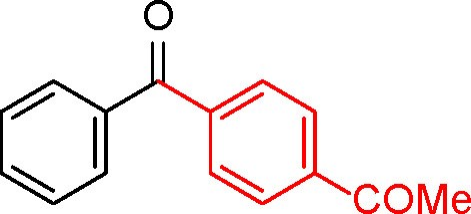	63
**25^[b]^**	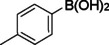	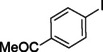	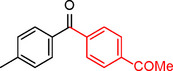	66
**26^[b]^**	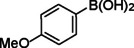	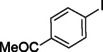	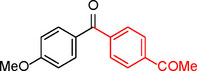	68
**27^[b]^**	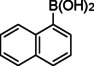	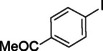	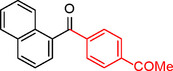	62
**28^[b]^**	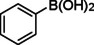	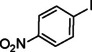	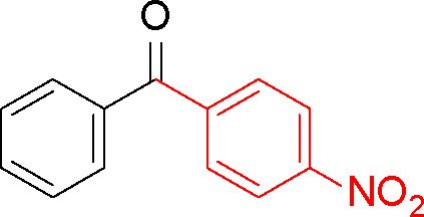	60
**29^[b]^**	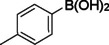	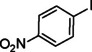	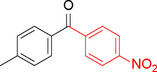	63
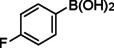		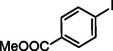	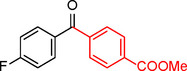	55

[a] Reaction conditions: 1 mol% catalyst, 1 mmol substrate, 1.1 mmol phenylboronic acid, 3 mmol K_2_CO_3_, 4 mL anisole, 100 psi of CO, 80 °C, 14 h; [b] Reaction was performed at 100 °C.

In entry 1, benzophenone was synthesized in high yield by coupling iodobenzene with phenylboronic acid at 80 °C under 100 psi of CO for 14 hours. Similarly, when phenylboronic acids bearing EDGs were reacted with iodobenzene (entries 2–3), excellent yields were achieved with exclusive formation of the carbonylative product. A slight decrease in yield was observed when the bulkier 1‐naphthylboronic acid was used as a coupling partner (entry 5). Comparable catalytic behavior was observed with 4‐iodoanisole, which produced high yields when coupled with 4‐methoxyphenylboronic acid (entry 7). However, the yield slightly decreased when 1‐naphthylboronic acid was used (entry 8), likely due to steric hindrance. Similar trends were observed when 4‐iodotoluene served as the substrate (entries 13–16).

In the case of methyl 4‐iodobenzoate, reactions with various phenylboronic acids (entries 9–12) afforded moderate to good yields. Notably, an EWG‐substituted phenylboronic acid (entry 12) required an elevated temperature of 100 °C to achieve good conversion, highlighting the impact of electronic effects on reactivity. Similarly, for 4‐iodoacetophenone (entries 17–20), better yields were obtained at 100 °C due to improved substrate activation at higher temperatures. Reactions involving strongly electron‐withdrawing substituents on iodoarenes, such as iodo‐4‐nitrobenzene, 4‐iodobenzonitrile, and iodo‐4‐(trifluoromethyl)benzene (entries 21–28), resulted in lower yields, attributed to reduced substrate reactivity. A similar trend was observed for 4‐fluorophenylboronic acid (entries 29–30), where fluorine‘s electron‐withdrawing nature negatively affected yield.

Interestingly, attempts to couple 4‐methoxycarbonylphenylboronic acid with various iodoarenes were unsuccessful. However, reversing the coupling partners by reacting methyl 4‐iodobenzoate with different substituted phenylboronic acids (entries 9–12) led to successful reactions, reinforcing the influence of substrate electronic properties and steric factors on reactivity.

**Figure 11:**
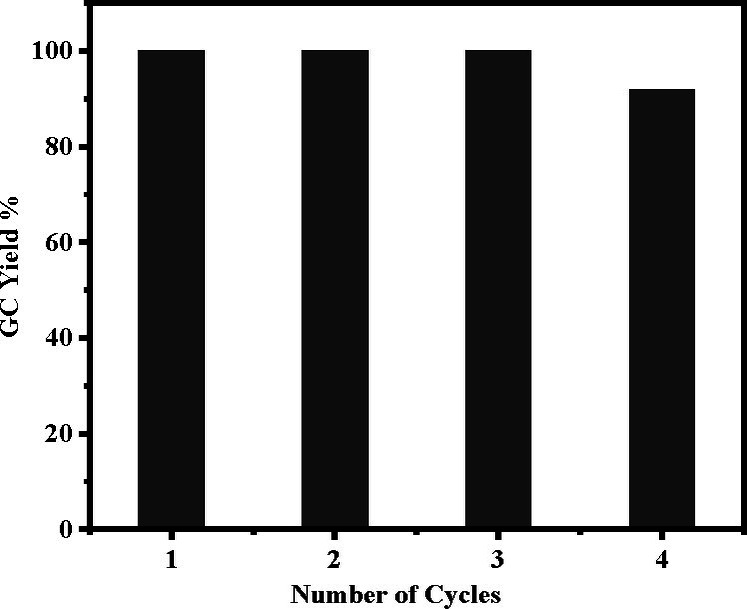
Pd_(np)_@PMO‐IL recyclability in the carbonylative Suzuki coupling of iodobenzene with phenylboronic acid. Reaction conditions: 1 mol% catalyst, 1 mmol iodobenzene, 1.1 mmol phenylboronic acid, 3 mmol K_2_CO_3_, 4 mL solvent, 100 psi of CO, 80 °C, 14 hours.

The recyclability of Pd_(np)_@PMO‐IL was also examined through four consecutive reactions of iodobenzene with phenylboronic acid (Figure 11). After each cycle, the catalyst was washed with anisole and reused without significant loss of activity. By the fourth cycle, a slight decrease of 8 % in yield was observed, attributed to minimal palladium leaching (0.8 ppm), as confirmed by inductively coupled plasma (ICP) analysis.

To assess the structural integrity of the catalyst after recycling, SEM and TEM analyses were performed following the fourth cycle. SEM images revealed no morphological changes (Supporting Information, Figure S6), while TEM analysis confirmed the continued presence of palladium nanoparticles within the PMO‐IL framework (Supporting Information, Figure S7). These findings demonstrate the structural stability and robustness of the catalyst even after multiple reaction cycles.

Additionally, the long‐term stability of Pd_(np)_@PMO‐IL was tested using a sample synthesized in 2016 and stored under ambient conditions. When applied to the carbonylative Suzuki coupling of iodobenzene with phenylboronic acid, the aged catalyst delivered full conversion with 100% selectivity toward the carbonyl product, as determined by GC analysis. Further SEM and TEM characterization confirmed that the catalyst retained its original structure (Supporting Information, Figures S8 and S9), underscoring its exceptional stability over extended storage periods.

## 3 Conclusions

This study successfully developed a novel heterogeneous catalytic system by supporting palladium nanoparticles (Pd NPs) on ionic liquid‐functionalized periodic mesoporous organosilica (PMO‐IL). Synthesized through a robust and straightforward sol‐gel process, the PMO‐IL material demonstrated excellent properties, including high surface area, thermal stability, and well‐ordered mesoporous structures, as confirmed by extensive characterization techniques such as TEM, SEM, FT‐IR, XRD, and NMR. The incorporation of ionic liquid groups into the silica framework enhanced the adsorption and stabilization of palladium nanoparticles, contributing to the catalyst‘s superior performance. The Pd_(np)_@PMO‐IL system exhibited remarkable activity and selectivity in carbonylative Suzuki coupling reactions under mild conditions, achieving high turnover numbers (TON) and turnover frequencies (TOF) across various substrates. Additionally, the catalyst displayed excellent recyclability, retaining most of its activity over multiple reaction cycles with minimal palladium leaching, thereby aligning with sustainable and green chemistry principles. Its heterogeneous nature facilitated easy recovery and reuse, enhancing its practicality. Furthermore, the PMO‐IL system demonstrated potential as a versatile platform for supporting other metal catalysts, with promising applications in various catalytic transformations.

## Conflict of Interests

The authors declare no conflict of interest.

4

## Supporting information

As a service to our authors and readers, this journal provides supporting information supplied by the authors. Such materials are peer reviewed and may be re‐organized for online delivery, but are not copy‐edited or typeset. Technical support issues arising from supporting information (other than missing files) should be addressed to the authors.

Supporting Information

## Data Availability

The data that support the findings of this study are available from the corresponding author upon reasonable request.
