# Supplementary material for: Ionic Liquid‐Functionalized Periodic Mesoporous Organosilica: A Robust Support for Palladium Nanoparticles in Carbonylative Suzuki Coupling Reactions
Source: Chem Asian J. 2025 Mar 19;20(10):e202401802. doi: 10.1002/asia.202401802 (PMC12128023; doi:10.1002/asia.202401802)
Supplement: Supplementary file 1 — Supporting Information [file ASIA-20-e202401802-s001.pdf]

Chemistry - An Asian Journal

Supporting Information

Ionic Liquid-Functionalized Periodic Mesoporous Organosilica: A Robust Support for Palladium Nanoparticles in Carbonylative Suzuki Coupling Reactions

Manan Sohanwal, Suheir Omar, and Raed Abu-Reziq*

Supporting Information

Ionic Liquid-Functionalized Periodic Mesoporous Organosilica: A Robust Support for Palladium Nanoparticles in Carbonylative Suzuki Coupling Reactions

*Manan Sohanwal, Suheir Omar and Raed Abu-Reziq**

Institute of Chemistry, Casali Center of Applied Chemistry, Center for Nanoscience and Nanotechnology, the Hebrew University of Jerusalem 9190401, Israel.

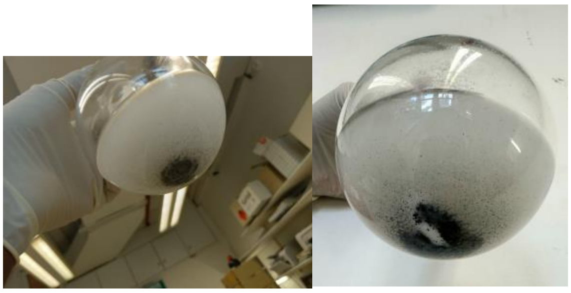

Figure S1. Photographs of the reaction system for the incorporation of palladium nanoparticle into PMO frameworks: PMO-Et (left) and PMO-Ph (right).

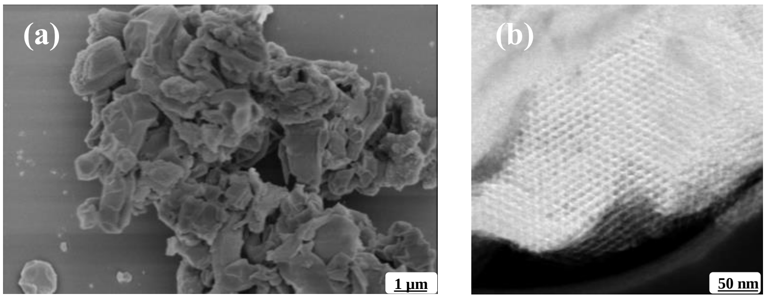

Figure S2. (a) SEM image of PMO-Ph after the incorporation of palladium. (b) TEM image of PMO-Ph after the incorporation of palladium.

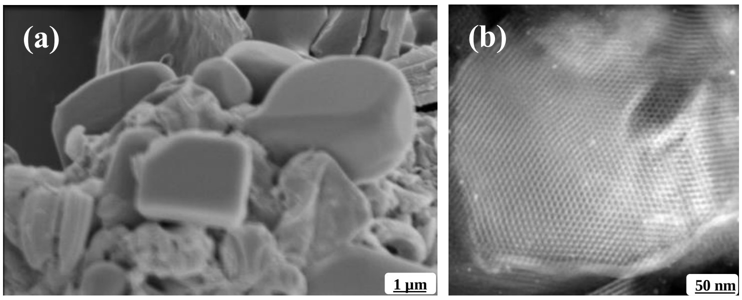

Figure S3. (a) SEM image of PMO-Et after the incorporation of palladium. (b) TEM image of PMO-Et after the incorporation of palladium.

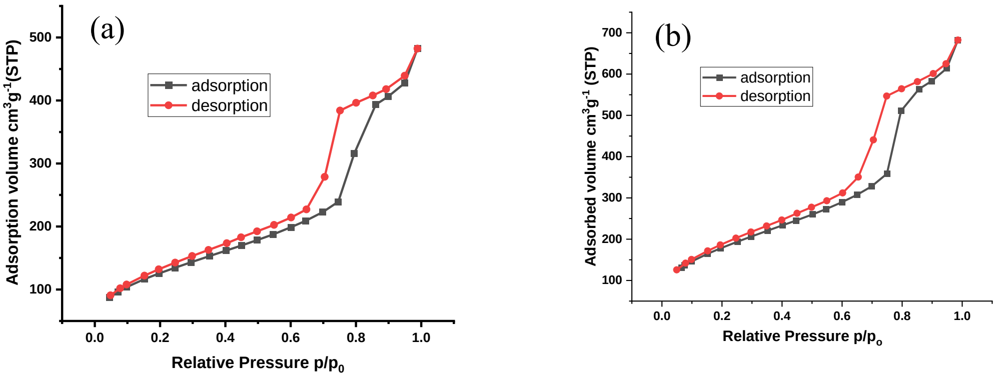

Figure S4. N_2 adsorption-desorption isotherms of (a) PMO-Ph before the incorporation of palladium. (b) PMO-Ph before the incorporation of palladium.

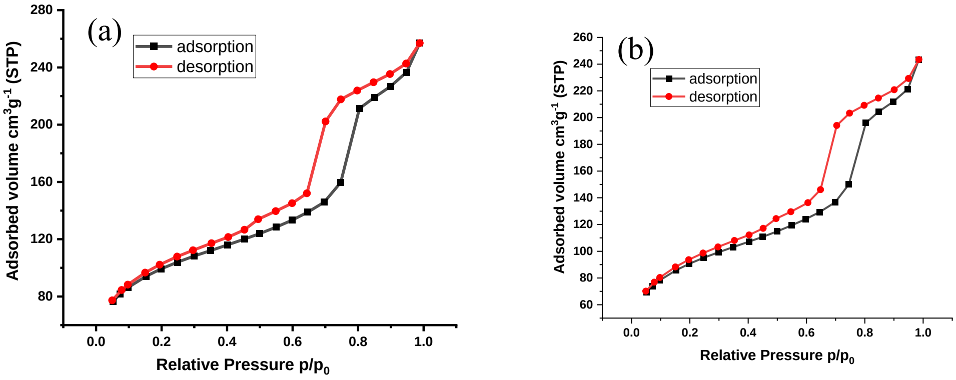

Figure S5. N_2 adsorption-desorption isotherms of (a) PMO-Et before the incorporation of palladium. (b) PMO-Et before the incorporation of palladium.

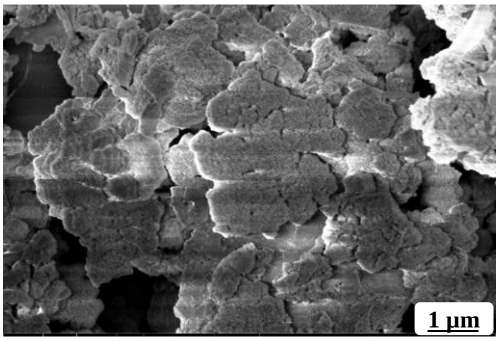

Figure S6. SEM image of $Pd_{(np)}$@PMO-IL after the fourth cycle.

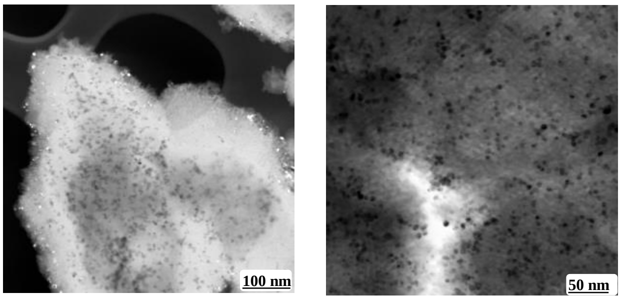

Figure S7. TEM images of $Pd_{(np)}$@PMO-IL after the fourth cycle.

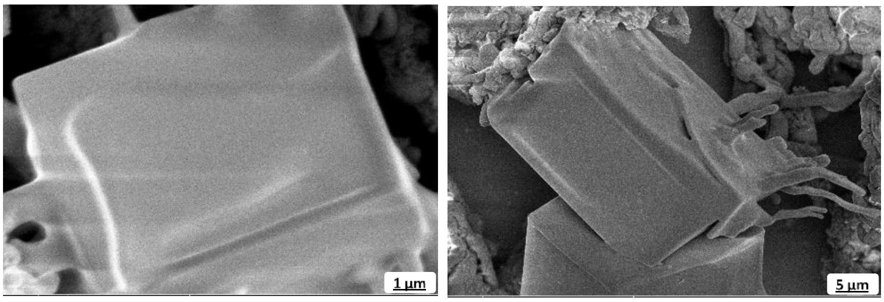

Figure S8. SEM images of $Pd_{(np)}$@PMO-IL prepared in 2016.

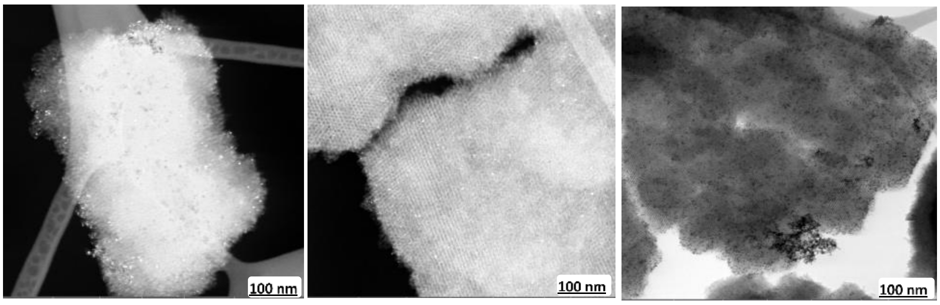

Figure S9. TEM images of $Pd_{(np)}$@PMO-IL prepared in 2016.

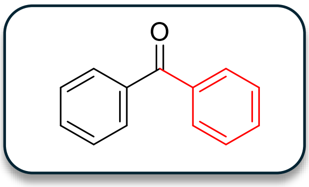

Benzophenone:[1] **^{1}H NMR** (400 MHz, $CDCl_3$) δ 7.63 – 7.58 (m, 4H), 7.41 – 7.36 (m, 2H), 7.31 – 7.25 (m,4H).**^{13}C NMR** (101 MHz, $CDCl_3$) δ 196.77, 137.63, 132.44, 130.08, 128.30.

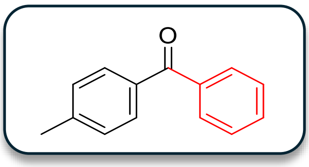

Phenyl(p-tolyl)methanone: [1] **^{1}H NMR** (400 MHz, $CDCl_3$) δ 7.79 (dd, *J* = 8.4, 1.4 Hz, 2H), 7.73 (d, *J* = 8.4 Hz, 2H), 7.60 – 7.55(m, 1H), 7.47 (t, *J* = 7.4 Hz, 2H), 7.28 (d, *J* = 7.8 Hz, 2H), 2.44 (s, 3H). **^{13}C NMR** (101 MHz, $CDCl_3$) δ 196.53, 143.26, 137.97, 134.90, 132.18, 130.33, 129.95, 129.00, 128.23, 21.68.

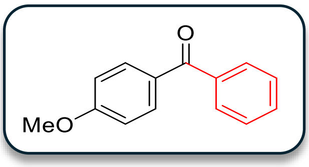

4-methoxyphenyl(phenyl)methanone: [1] **^{1}H NMR** (400 MHz, $CDCl_3$) δ 7.83 (d, *J* = 9.0 Hz, 2H), 7.76 (dd, *J* = 8.3, 1.3 Hz, 2H), 7.59 – 7.53 (m,1H), 7.47 (t, *J* = 7.4 Hz, 2H), 6.96 (d, *J* = 9.0 Hz, 2H), 3.88 (s, 3H). **^{13}C NMR** (101 MHz, $CDCl_3$) δ 195.58, 163.25, 138.31, 132.58, 131.91, 130.18, 129.74, 128.20, 113.58, 55.51.

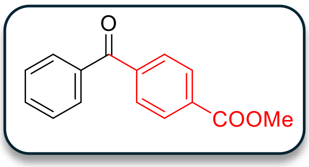

Methyl-4-benzoylbenzoate: [1] **^{1}H NMR** (400 MHz, $CDCl_3$) δ 8.15 (d, *J* = 8.8 Hz, 2H), 7.86 – 7.79 (m, 4H), 7.64 – 7.59 (m, 1H), 7.50 (t, *J* = 7.6 Hz, 2H), 3.97 (s, 3H). **^{13}C NMR** (101 MHz, $CDCl_3$) δ 196.06, 166.34, 141.33, 136.96, 133.23, 132.96, 130.12, 129.79, 129.52, 128.48, 52.49.

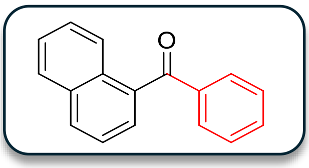

Napthalen-1-yl(phenyl)methanone: [1] **^{1}H NMR** (400 MHz, $CDCl_3$) δ 8.11 (d, *J* = 7.5 Hz, 1H), 8.01 (d, *J* = 8.3 Hz, 1H), 7.95 – 7.91 (m, 1H), 7.90 – 7.85 (m, 2H), 7.59 (d, *J* = 8.4 Hz, 2H), 7.57 – 7.49 (m, 3H), 7.46 (t, *J* = 7.8 Hz, 2H). **^{13}C NMR** (101 MHz, $CDCl_3$) δ 198.06, 138.35, 136.39, 133.75, 133.26, 131.29, 130.99, 130.43, 128.48, 128.43, 127.79, 127.28, 126.49, 125.72, 124.36.

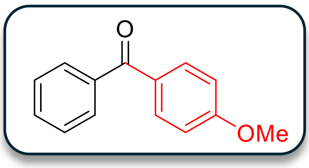

4-methoxyphenyl(phenyl)methanone: [2] **^{1}H NMR** (400 MHz, $CDCl_3$) δ 7.83 (d, *J* = 9.0 Hz, 2H), 7.76 (dd, *J* = 8.3, 1.3 Hz, 2H), 7.59 – 7.53 (m,1H), 7.47 (t, *J* = 7.4 Hz, 2H), 6.96 (d, *J* = 9.0 Hz, 2H), 3.88 (s, 3H). **^{13}C NMR** (101 MHz, $CDCl_3$) δ 195.58, 163.25, 138.31, 132.58, 131.91, 130.18, 129.74, 128.20, 113.58, 55.51.

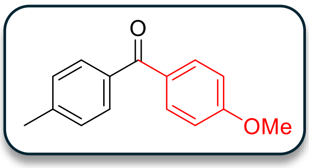

4-methoxyphenyl(p-tolyl)methaone: [1] **^{1}H NMR** (400 MHz, $CDCl_3$) δ 7.73 (d, *J* = 9.0 Hz, 2H), 7.59 (d, *J* = 8.3 Hz, 2H), 7.18 (d, *J* = 7.8 Hz, 2H), 6.87 (d, *J* = 9.0 Hz, 2H), 3.79 (s, 3H), 2.35 (s, 3H). **^{13}C NMR** (101 MHz, $CDCl_3$) δ 195.40, 163.06, 142.63, 135.52, 132.44, 130.49, 130.01, 128.89, 113.50, 55.49, 21.62.

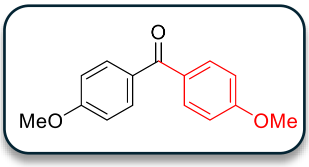

Bis(4-methoxyphenyl)methanone: [3] **^{1}H NMR** (400 MHz, $CDCl_3$) δ 7.79 (d, J = 8.9 Hz, 4H), 6.96 (d, J = 8.9 Hz, 4H), 3.88 (s, 6H).**^{13}C NMR** (101 MHz, $CDCl_3$) δ 194.49, 162.85, 132.24, 130.78, 113.47, 55.48.

O

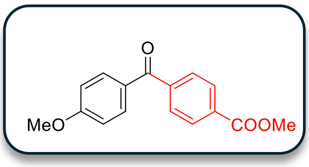

Methyl-4(4-methoxybenzoyl)benzoate: [1] **^{1}H NMR** (400 MHz, $CDCl_3$) δ 8.14 (d, J = 8.6 Hz, 2H), 7.80 (dd, J = 12.8, 8.8 Hz, 4H), 6.97 (d, J = 9.0 Hz, 2H), 3.96 (s, 3H), 3.89 (s, 3H). **^{13}C NMR** (101 MHz, $CDCl_3$) δ 194.81, 166.42, 163.64, 142.16, 132.78, 132.64, 129.58, 129.45, 113.76, 55.57, 52.45.

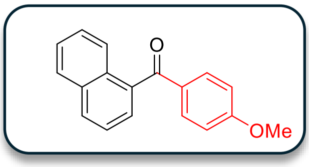

4-methoxyphenyl(naphthalen-1-yl)methanone: [4] **^{1}H NMR** (400 MHz, $CDCl_3$) δ 8.03 – 7.83 (m, 5H), 7.57 – 7.45 (m, 4H), 6.93 (d, J = 9.0 Hz, 2H), 3.87(s, 3H). **^{13}C NMR** (101 MHz, $CDCl_3$) δ 195.70, 162.78, 136.02, 132.64, 131.77, 130.06, 129.86, 129.61, 127.31, 125.98, 125.78, 125.33, 124.70, 123.40, 112.67, 54.50.

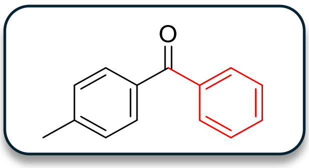

Phenyl(p-tolyl)methanone: [1] **^{1}H NMR** (400 MHz, $CDCl_3$) δ 7.79 (dd, J = 8.4, 1.4 Hz, 2H), 7.73 (d, J = 8.4 Hz, 2H), 7.60 – 7.55(m, 1H), 7.47 (t, J = 7.4 Hz, 2H), 7.28 (d, J = 7.8 Hz, 2H), 2.44 (s, 3H). **^{13}C NMR** (101 MHz, $CDCl_3$) δ 196.53, 143.26, 137.97, 134.90, 132.18, 130.33, 129.95, 129.00, 128.23, 21.68.

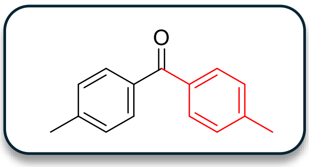

di-(p-tolyl)methanone: [3] **^{1}H NMR** (400 MHz, $CDCl_3$) δ 7.62 (d, J = 8.3 Hz, 4H), 7.18 (d, J = 8.4 Hz, 4H), 2.35 (s, 6H).**^{13}C NMR** (101 MHz, $CDCl_3$) δ 196.31, 142.95, 135.24, 130.21, 128.92, 21.65.

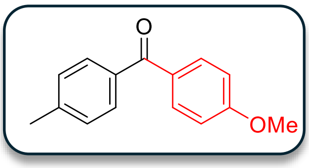

4-methoxyphenyl(p-tolyl)methanone: [1] **^{1}H NMR** (400 MHz, $CDCl_3$) δ 7.74 (d, J = 8.9 Hz, 2H), 7.60 (d, J = 8.3 Hz, 2H), 7.20 (d, J = 7.6 Hz, 2H), 6.88 (d, J = 9.0 Hz, 2H), 3.81 (s, 3H), 2.36 (s, 3H). **^{13}C NMR** (101 MHz, $CDCl_3$) δ 195.40, 163.06, 142.63, 135.52, 132.44, 130.49, 130.01, 128.89, 113.50, 55.49, 21.62.

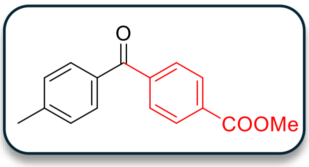

Methyl-4(4-methylbenzoyl)benzoate: [1] **^{1}H NMR** (400 MHz, $CDCl_3$) δ 8.14 (d, J = 8.8 Hz, 2H), 7.81 (d, J = 8.6 Hz, 2H), 7.71 (d, J = 8.3 Hz, 2H), 7.29 (d, J = 7.6 Hz, 2H), 3.96 (s, 3H), 2.45 (s, 3H).**^{13}C NMR** (101 MHz, $CDCl_3$) δ 195.81, 166.39, 143.93, 141.75, 134.29, 133.00, 130.35, 129.66, 129.47, 129.18, 52.46, 21.72.

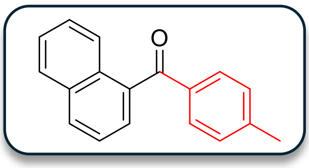

Naphthalen-1-yl(p-tolyl)mathanone: [5] **^{1}H NMR** (400 MHz, $CDCl_3$) δ 7.74 (d, J = 8.9 Hz, 2H), 7.60 (d, J = 8.3 Hz, 2H), 7.20 (d, J = 7.6 Hz, 2H), 6.88 (d, J = 9.0 Hz, 2H), 3.81 (s, 3H), 2.36 (s, 3H). **^{13}C NMR** (101 MHz, $CDCl_3$) δ 196.63, 143.13, 135.67, 134.64, 132.61, 129.88, 129.49, 128.10, 127.30, 126.25, 126.03, 125.32, 124.64, 123.30, 20.64.

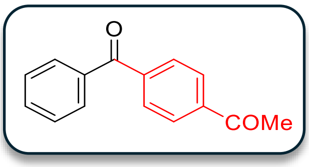

1-(4-benzoylphenyl)ethan-1-one: [1] **^{1}H NMR** (400 MHz, $CDCl_3$) δ 8.07 – 8.05 (m, 2H), 7.88 – 7.85 (m, 2H), 7.81 (dd, *J* = 8.4, 1.3 Hz, 2H), 7.65 – 7.60 (m, 1H), 7.53 – 7.48 (m, 2H), 2.67 (s, 3H). **^{13}C NMR** (101 MHz, $CDCl_3$) δ 197.53, 195.97, 141.36, 139.58, 136.94, 133.00, 130.12, 130.06, 128.49, 128.18, 26.92.

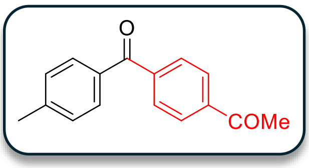

1-(4-(4-methylbezoyl)phenyl)ethan-1-one: [3] **^{1}H NMR** (400 MHz, $CDCl_3$) δ 8.05 (d, *J* = 8.8 Hz, 2H), 7.84 (d, *J* = 8.6 Hz, 2H), 7.72 (d, *J* = 8.1 Hz, 2H), 7.30 (d, *J* = 7.9 Hz, 2H), 2.67 (s, 3H), 2.45 (s, 3H). **^{13}C NMR** (101 MHz, $CDCl_3$) δ 197.58, 195.71, 143.98, 141.78, 139.40, 134.26, 130.35, 129.92, 129.19, 128.13, 26.90, 21.72.

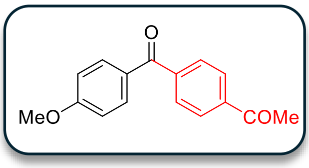

1-(4-(4-methoxybezoyl)phenyl)ethan-1-one: [3] **^{1}H NMR** (400 MHz, $CDCl_3$) δ 8.04 (d, *J* = 8.6 Hz, 2H), 7.82 (d, *J* = 3.5 Hz, 2H), 7.80 (d, *J* = 3.3 Hz, 2H), 6.97 (d, *J* = 9.0 Hz, 2H), 3.89 (s, 3H), 2.66 (s, 3H). **^{13}C NMR** (101 MHz, $CDCl_3$) δ 197.59, 194.69, 163.66, 142.19, 139.20, 132.62, 129.70, 129.54, 128.13, 113.78, 55.57, 26.88.

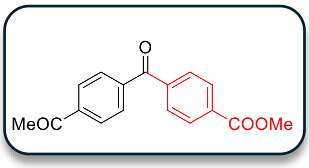

Methyl-4-(4-acetylbenzoyl)benzoate: [1] **^{1}H NMR** (400 MHz, $CDCl_3$) δ 8.16 (d, *J* = 8.1 Hz, 2H), 8.07 (d, *J* = 8.8 Hz, 2H), 7.86 (dd, *J* = 9.9, 8.1 Hz, 4H), 3.97 (s, 3H), 2.67 (s, 3H). **^{13}C NMR** (101 MHz, $CDCl_3$) δ 197.41, 195.28, 166.17, 140.56, 140.52, 139.95, 133.74, 130.14, 129.86, 129.68, 128.31, 52.55, 26.93.

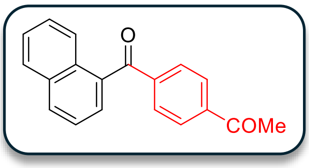

1-(4-(1-napthoyl)phenyl)ethan-1-one: [6] **^{1}H NMR** (400 MHz, $CDCl_3$) δ 8.13 (dd, *J* = 8.6, 1.1 Hz, 1H), 8.06 – 8.01 (m, 3H), 7.96 – 7.91 (m, 3H), 7.60 – 7.50 (m, 4H), 2.66 (s, 3H). **^{13}C NMR** (101 MHz, $CDCl_3$) δ 197.56, 197.26, 141.86, 140.13, 135.54, 133.79, 132.02, 130.90, 130.49, 128.54, 128.49, 128.30, 127.60, 126.67, 125.56, 124.32, 26.95.

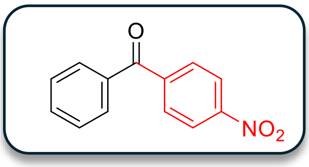

4-nitrophenyl(phenyl)mathanone: [1] **^{1}H NMR** (400 MHz, $CDCl_3$) δ 8.34 (d, *J* = 9.0 Hz, 2H), 7.94 (d, *J* = 9.0 Hz, 2H), 7.80 (dd, *J* = 8.4, 1.3 Hz, 2H), 7.68 – 7.63 (m, 1H), 7.55 – 7.50 (m, 2H). **^{13}C NMR** (101 MHz, $CDCl_3$) δ 194.82, 149.85, 142.90, 136.30, 133.49, 130.71, 130.12, 128.70, 123.56.

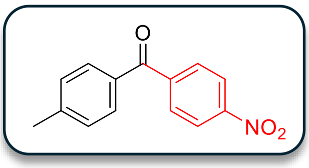

4-nitrophenyl(p-tolyl)mathanone: [7] **^{1}H NMR** (400 MHz, $CDCl_3$) δ 8.32 (d, *J* = 8.9 Hz, 2H), 7.87 (d, *J* = 8.9 Hz, 2H), 7.81 (d, *J* = 9.0 Hz, 2H), 6.99 (d, *J* = 9.0 Hz, 2H), 3.90 (s, 3H). **^{13}C NMR** (101 MHz, $CDCl_3$) δ 193.49, 164.03, 149.53, 143.81, 132.67, 130.34, 128.93, 123.49, 113.99, 55.64

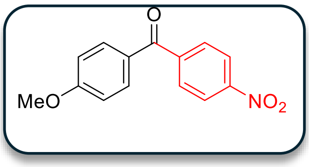

4-methoxyphenyl(4-nitrophenyl)mathanone: [7] **^{1}H NMR** (400 MHz, $CDCl_3$) δ 8.32 (d, *J* = 8.9 Hz, 2H), 7.90 (d, *J* = 8.9 Hz, 2H), 7.71 (d, *J* = 8.4 Hz, 2H), 7.32 (d, *J* = 7.8 Hz, 2H), 2.46 (s, 3H). **^{13}C NMR** (101 MHz, $CDCl_3$) δ 194.52, 149.70, 144.59, 143.34, 133.65, 130.56, 130.34, 129.40, 123.50, 21.76.

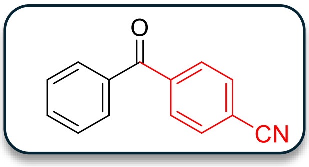

4-benzoylbenzonitrile: [1] **^{1}H NMR** (400 MHz, $CDCl_3$) δ 7.87 (d, *J* = 8.6 Hz, 2H), 7.83 – 7.72 (m, 4H), 7.66 – 7.61 (m, 1H), 7.51 (t, *J* = 7.6 Hz, 2H). **^{13}C NMR** (101 MHz, $CDCl_3$) δ 195.05, 141.24, 136.34, 133.35, 132.19, 130.25, 130.08, 128.66, 118.04, 115.66.

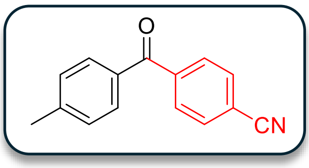

4-(4-methylbenzoyl)benzonitrile: [6] **^{1}H NMR** (400 MHz, $CDCl_3$) δ 7.84 (d, *J* = 8.4 Hz, 2H), 7.77 (d, *J* = 8.4 Hz, 2H), 7.69 (d, *J* = 8.1 Hz, 2H), 7.30 (d, *J* = 8.1 Hz, 2H), 2.45 (s, 3H). **^{13}C NMR** (101 MHz, $CDCl_3$) δ 194.78, 144.42, 141.66, 133.67, 132.13, 130.32, 130.13, 129.35, 118.10, 115.41, 21.76.

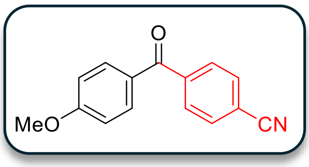

4-(4-methoxybenzoyl)benzonitrile: [6] **^{1}H NMR** (400 MHz, $CDCl_3$) δ 7.83 – 7.74 (m, 6H), 6.97 (d, *J* = 9.0 Hz, 2H), 3.89 (s, 3H). **^{13}C NMR** (101 MHz, $CDCl_3$) δ 193.73, 163.92, 142.11, 132.63, 132.11, 129.93, 128.96, 118.12, 115.15, 113.95, 55.62.

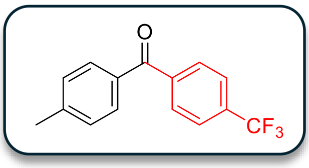

p-tolyl(4-(trifluoromethyl)phenylmethanone: [4] **^{1}H NMR** (400 MHz, $CDCl_3$) δ 7.87 (d, *J* = 7.9 Hz, 2H), 7.78 – 7.68 (m, 4H), 7.30 (d, *J* = 7.9 Hz, 2H), 2.45 (s, 3H). **^{13}C NMR** (101 MHz, $CDCl_3$) δ 195.27, 144.11, 141.14, 134.07, 130.35, 130.02, 129.24, 125.35, 125.31, 125.27, 125.23, 21.70.

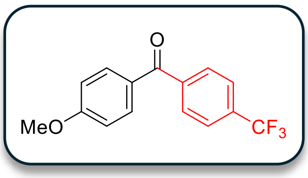

4-methoxyphenyl(4-(trifluoromethyl)phenyl)mathanone: [4] **^{1}H NMR** (400 MHz, $CDCl_3$) δ 7.86 – 7.79 (m, 4H), 7.74 (d, *J* = 8.1 Hz, 2H), 6.98 (d, *J* = 9.0 Hz, 2H), 3.90 (s, 3H). **^{13}C NMR** (101 MHz, $CDCl_3$) δ 194.29, 163.74, 141.54, 132.65, 129.80, 129.36, 125.32, 125.28, 125.25, 125.21, 113.83, 55.57.

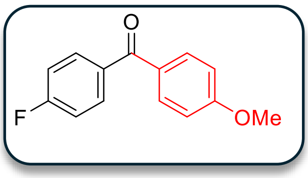

4-fluorophenyl(4-methoxyphenyl)methanone: [8] **^{1}H NMR** (400 MHz, $CDCl_3$) δ 7.80 (dt, *J* = 8.9, 2.7 Hz, 4H), 7.20 – 7.11 (m, 2H), 6.97 (d, *J* = 9.0 Hz, 2H), 3.89 (s, 3H). **^{13}C NMR** (101 MHz, $CDCl_3$) δ 194.15, 166.34, 163.82, 163.28, 134.47, 134.44, 132.43, 132.35, 132.26, 130.04, 115.45, 115.23, 113.65, 55.53.

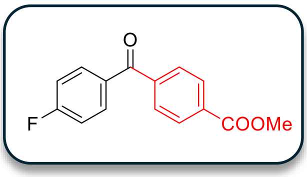

Methyl 4-(4-fluorobenzoyl)benzoate: [2] **^{1}H NMR** (400 MHz, $CDCl_3$) δ 8.17 – 8.13 (m, 2H), 7.87 – 7.78 (m, 4H), 7.21 – 7.14 (m, 2H), 3.96 (s, 3H). **^{13}C NMR** (101 MHz, $CDCl_3$) δ 194.54, 166.96, 166.25, 164.42, 141.22, 133.31, 133.23, 133.20, 132.81, 132.72, 129.61, 129.58, 115.82, 115.60, 52.50.

Reference

[1] X. Qi, L. Jiang, H. Li, X. Wu, *Chem. – Eur. J.* **2015**, *21*, 17650–17656.

[2] T. Ishiyama, H. Kizaki, T. Hayashi, A. Suzuki, N. Miyaura, *J. Org. Chem.* **1998**, *63*, 4726–4731.

[3] M. Mondal, A. Saha, *J. Org. Chem.* **2025**, *90*, 52–58.

[4] K. Zhang, Y. Yao, H. Sun, W. Zhang, Z. Gao, *J. Organomet. Chem.* **2024**, *1015*, 123181.

[5] P. Gautam, M. Dhiman, V. Polshettiwar, B. M. Bhanage, *Green Chem.* **2016**, *18*, 5890–5899.

[6] S. N. Jadhav, A. S. Kumbhar, C. V. Rode, R. S. Salunkhe, *Green Chem.* **2016**, *18*, 1898–1911.

[7] S. Chand, A. K. Sharma, A. K. Pandey, K. N. Singh, *Chem. Commun.* **2023**, *59*, 14827–14830.

[8] S. Wang, P. Xu, Z.-T. Liu, Y.-Q. Liu, H.-Q. Jiang, T.-Z. Hao, H.-X. Jiang, H. Xu, X.-D. Cao, D. Guo, X. Zhu, *ACS Cent. Sci.* **2025**, *11*, 46–56.

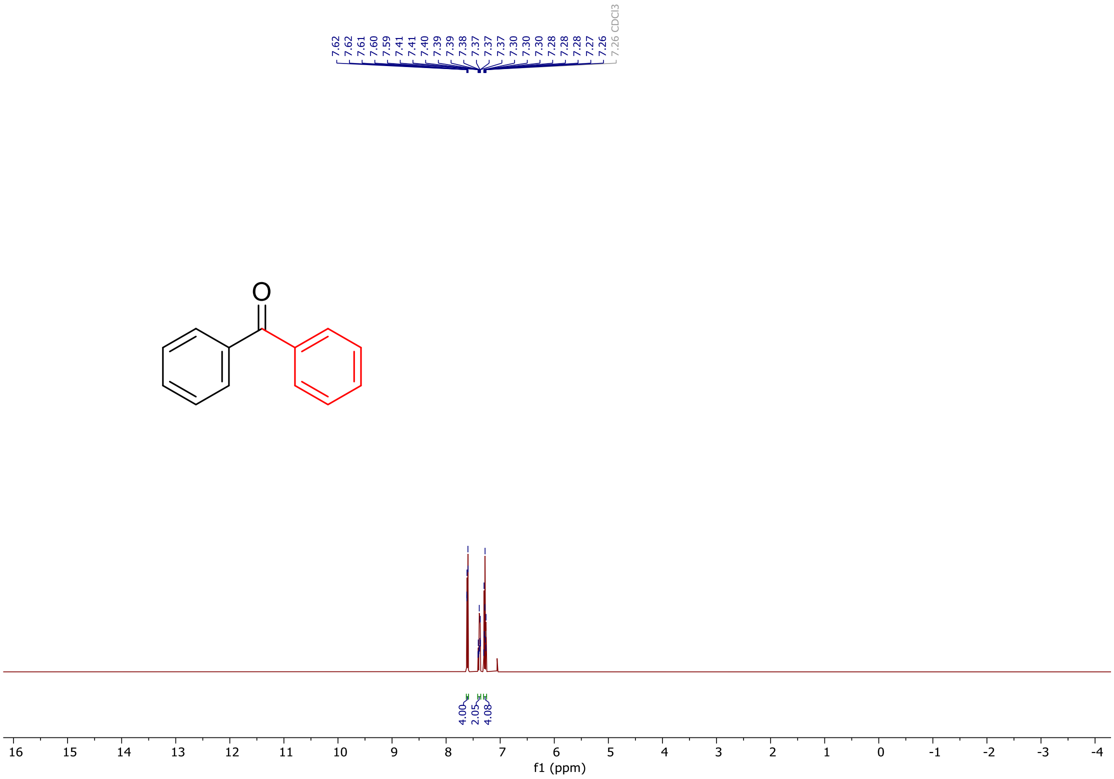

Figure S10. ^{1}H NMR of benzophenone.

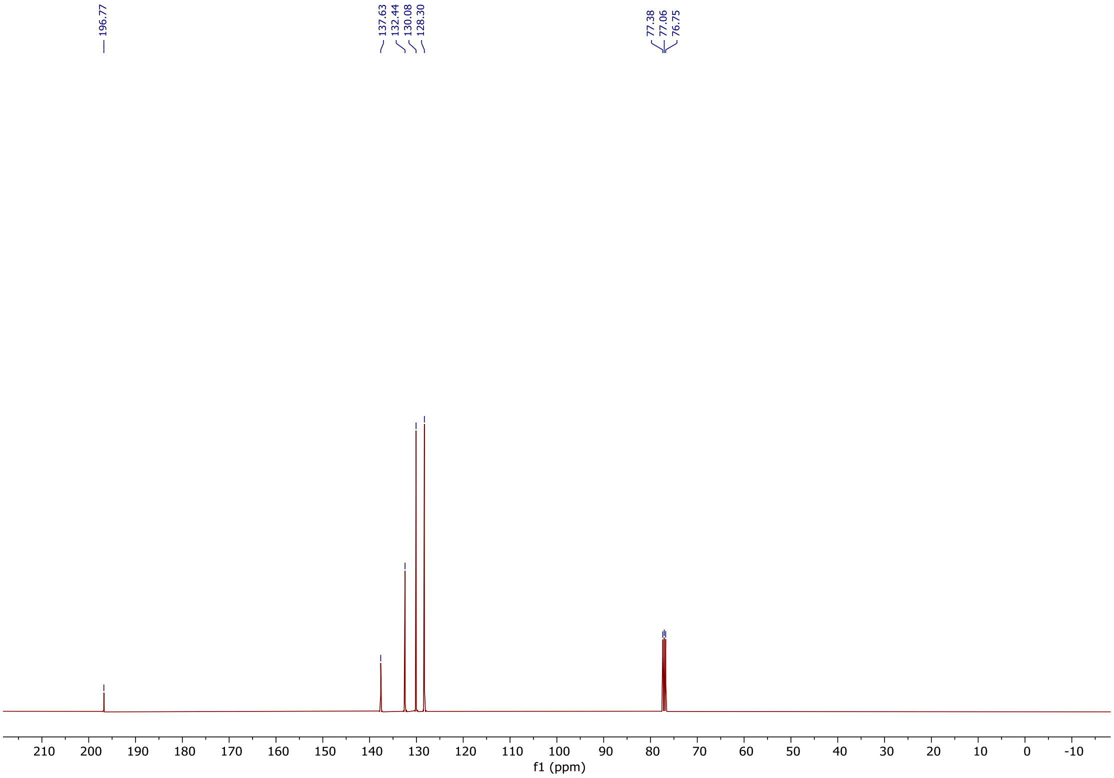

Figure S11. ^{13}C NMR of benzophenone.

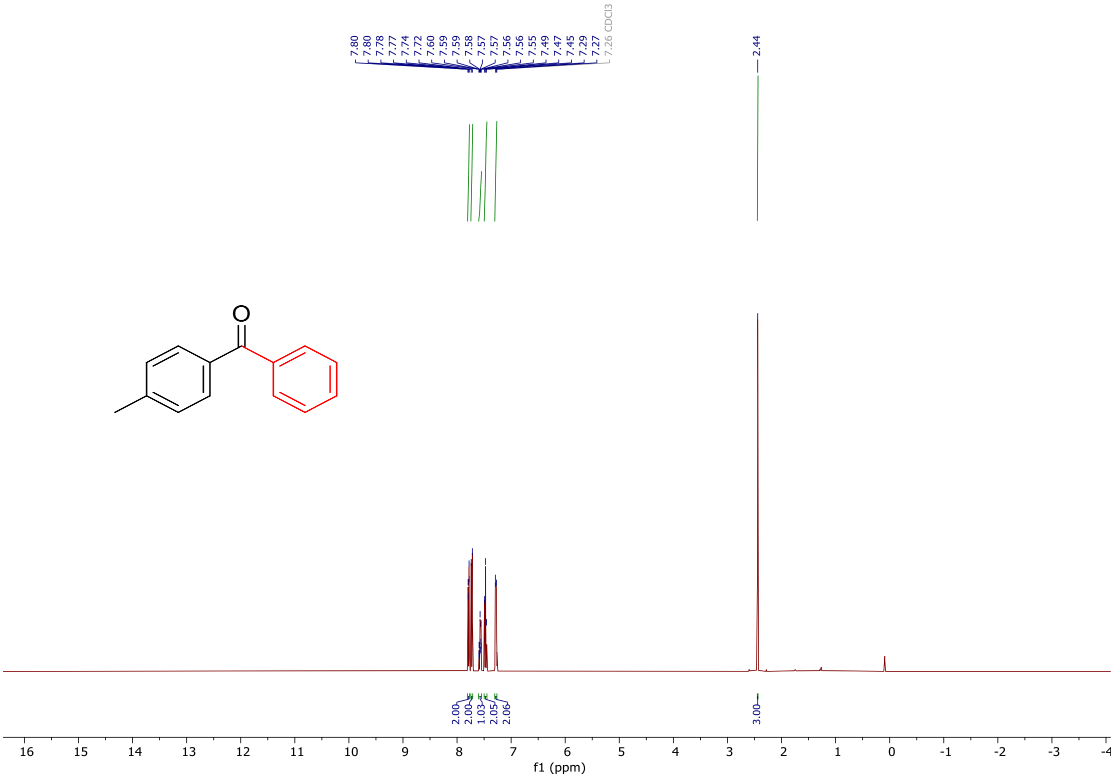

Figure S12. ^{1}H NMR of phenyl(p-tolyl)methanone

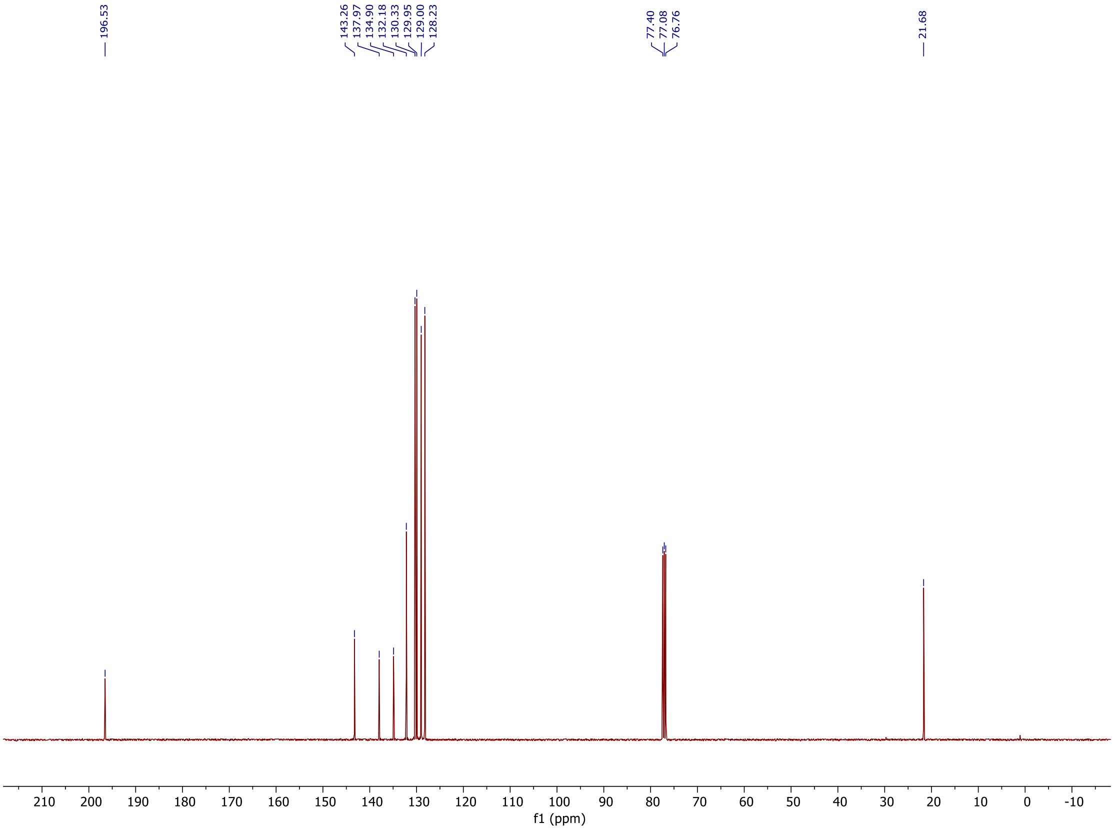

Figure S13. ^{13}C NMR of phenyl(p-tolyl)methanone

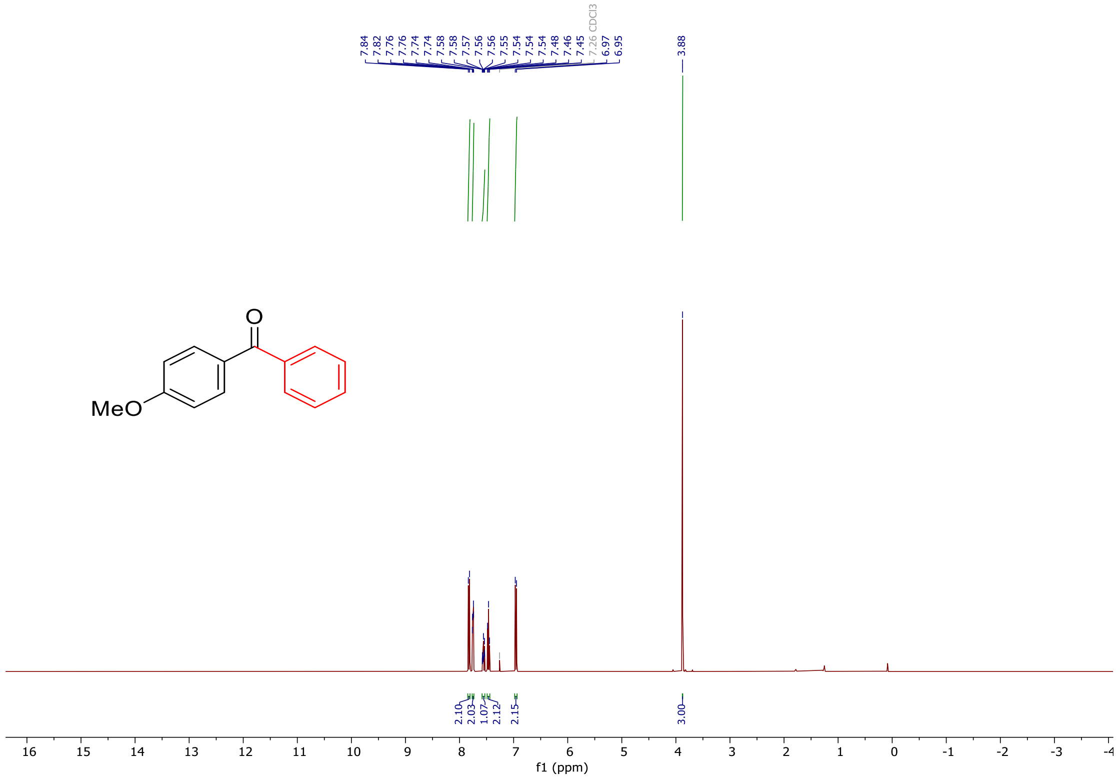

Figure S14. ^{1}H NMR of 4-methoxyphenyl(phenyl)methanone

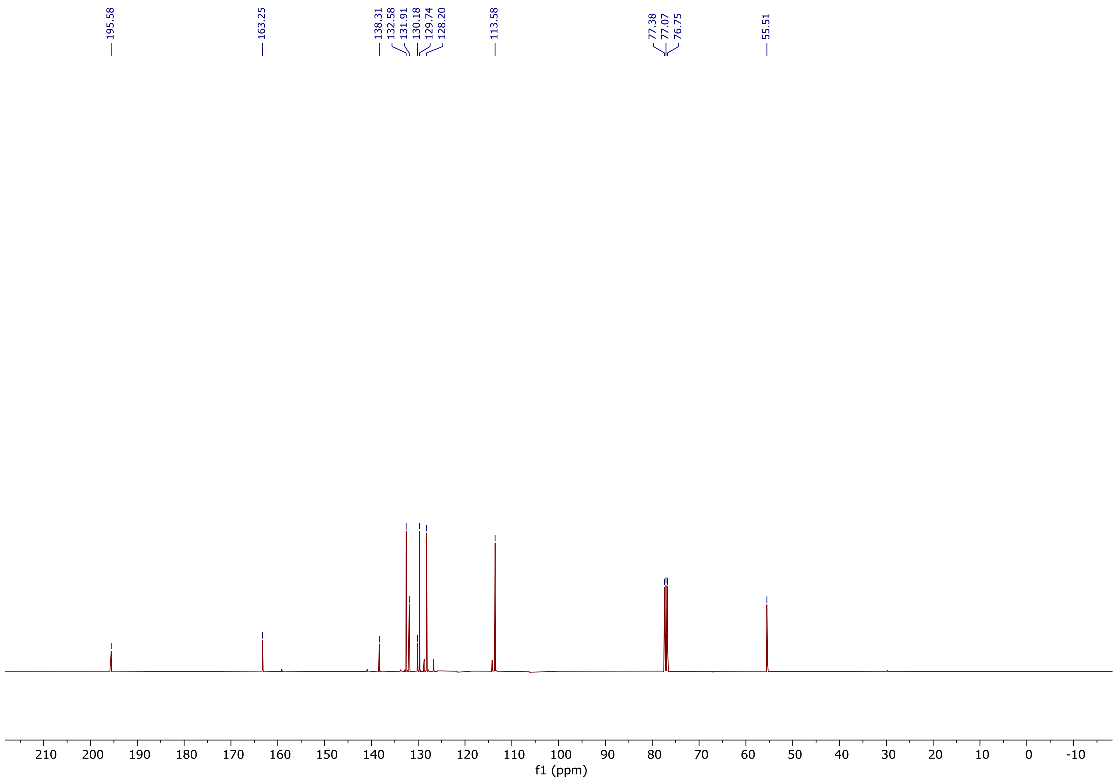

Figure S15. ^{13}C NMR of 4-methoxyphenyl(phenyl)methanone

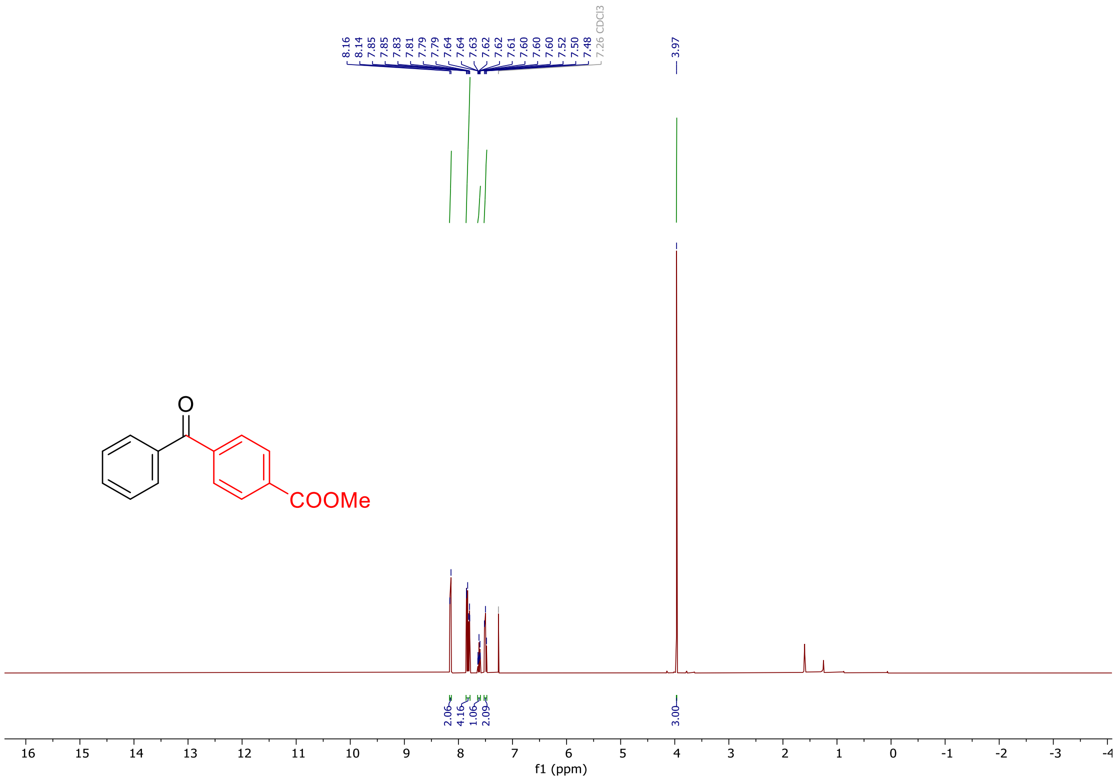

Figure S16. ^{1}H NMR of methyl-4-benzoylbenzoate

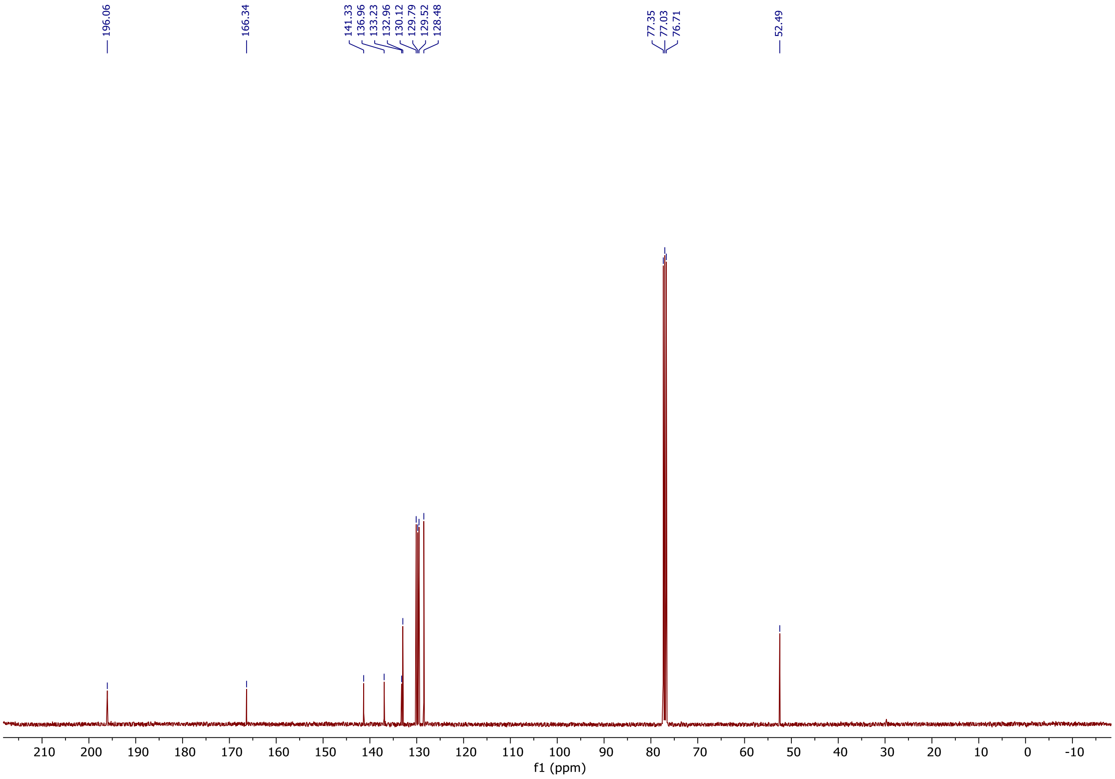

Figure S17. ^{13}C NMR of methyl-4-benzoylbenzoate

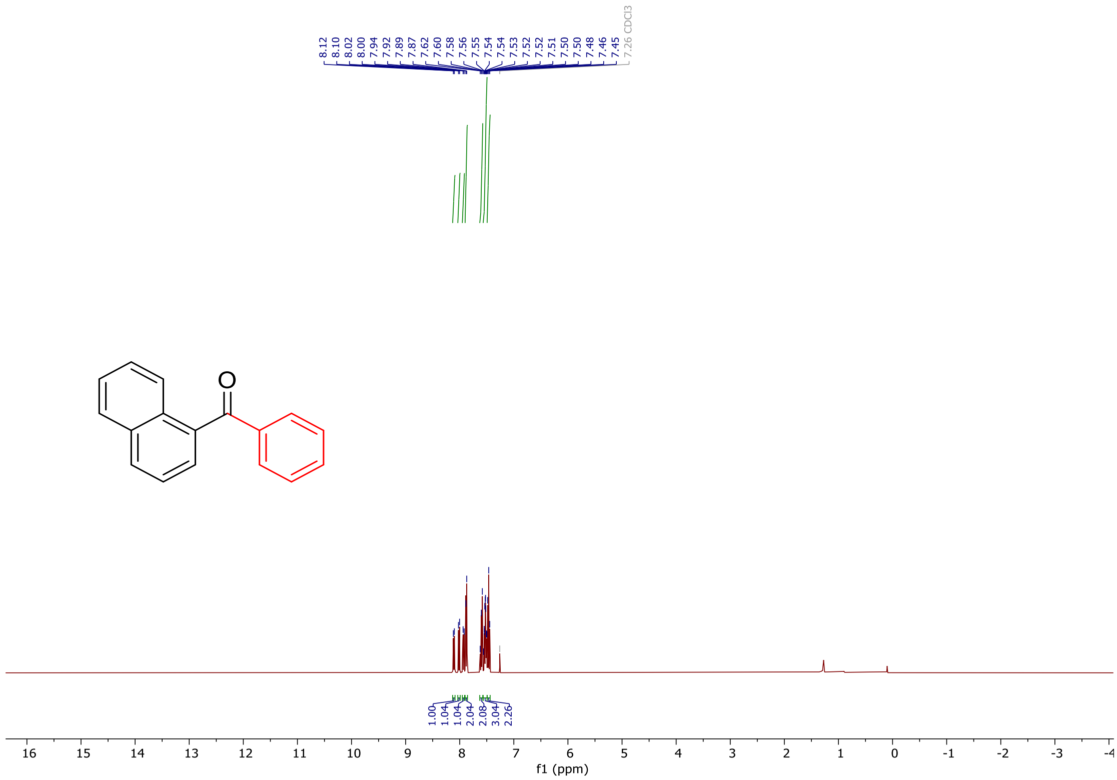

Figure S18. ^{1}H NMR of napthalen-1-yl(phenyl)methanone

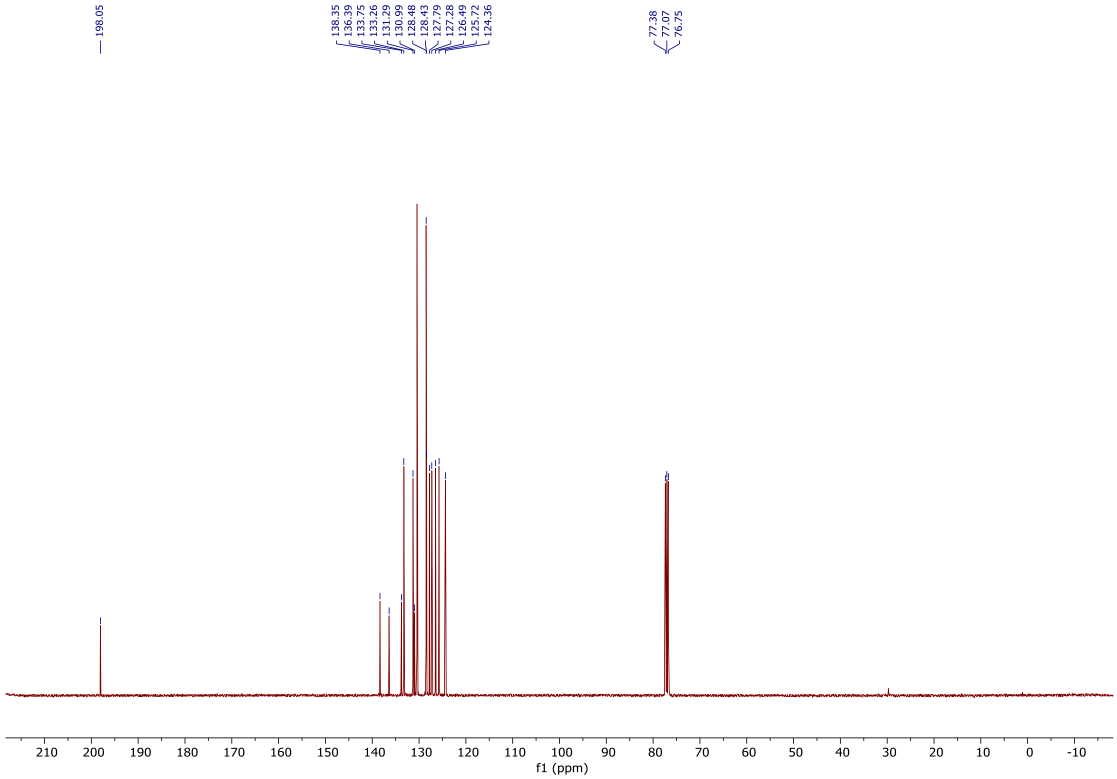

Figure S2. ^{13}C NMR of napthalen-1-yl(phenyl)methanone

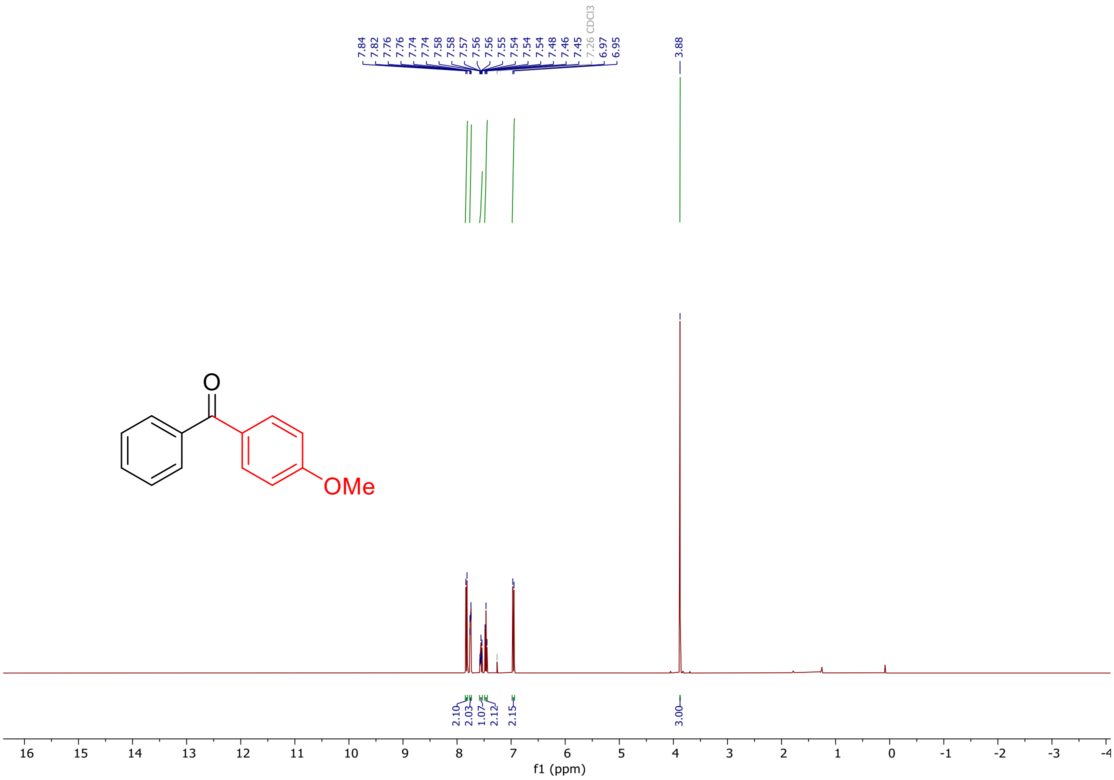

Figure S20. ^{1}H NMR of 4-methoxyphenyl(phenyl)methanone

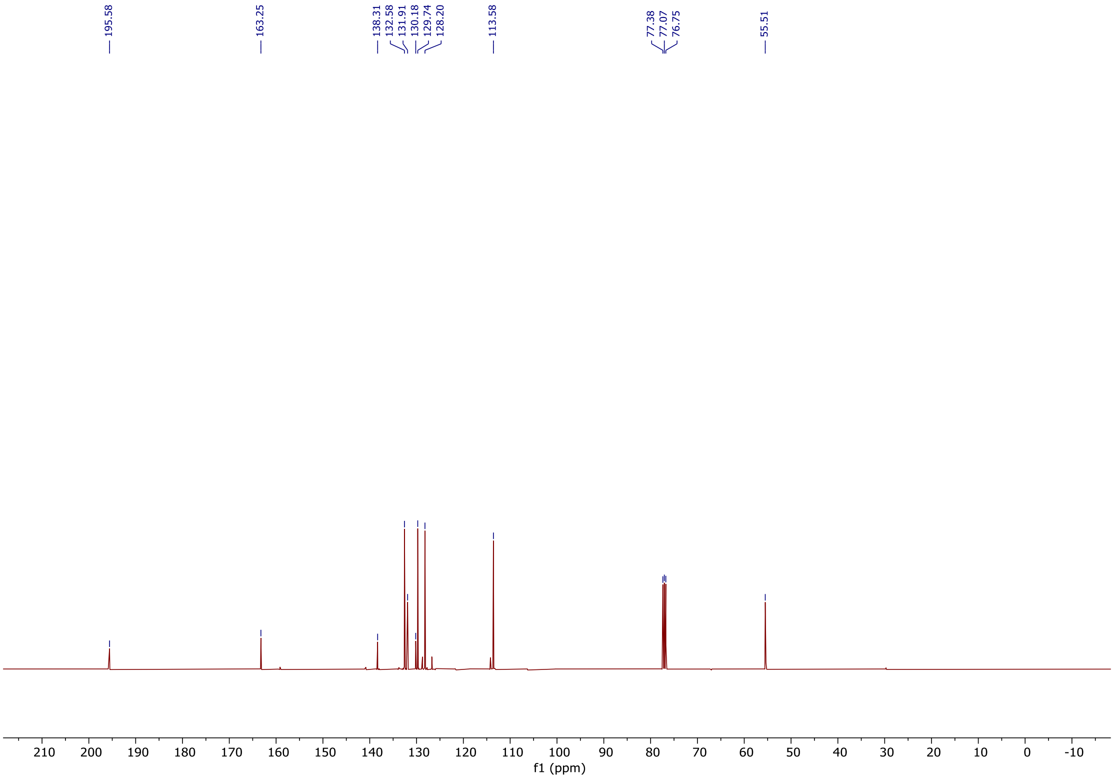

Figure S21. ^{13}C NMR of 4-methoxyphenyl(phenyl)methanone

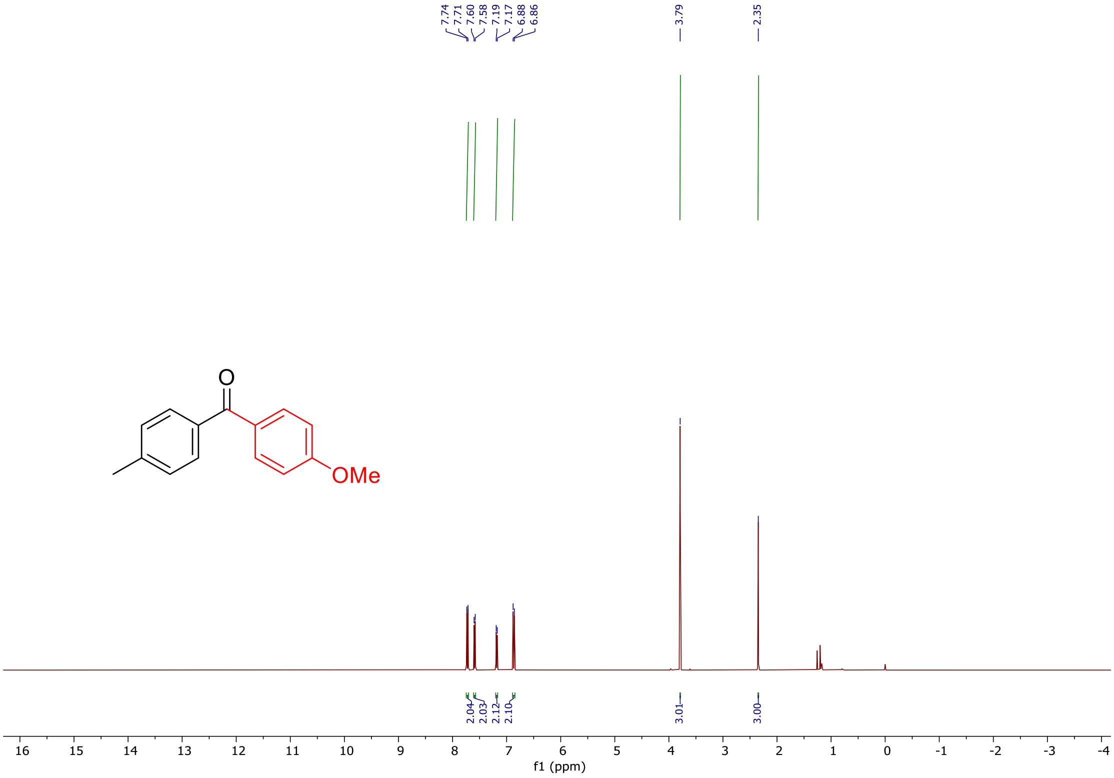

Figure S22. ^{1}H NMR of 4-methoxyphenyl(p-tolyl)methanone

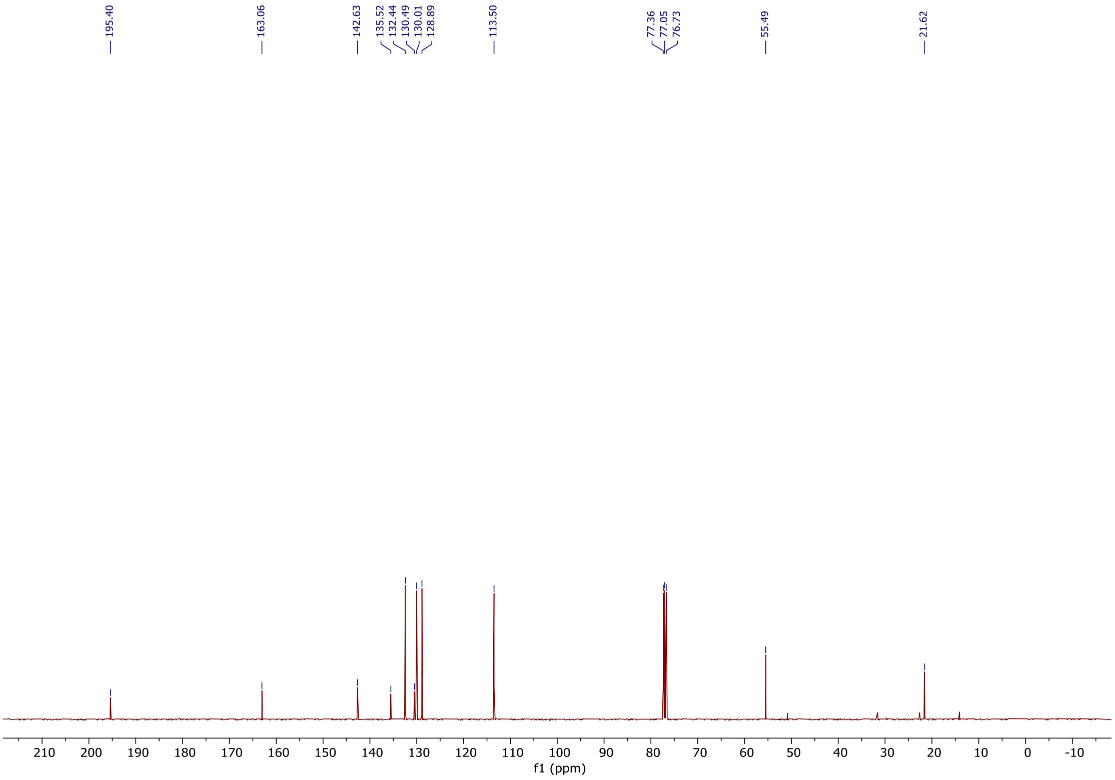

Figure S23. ^{13}C NMR of 4-methoxyphenyl(p-tolyl)methanone

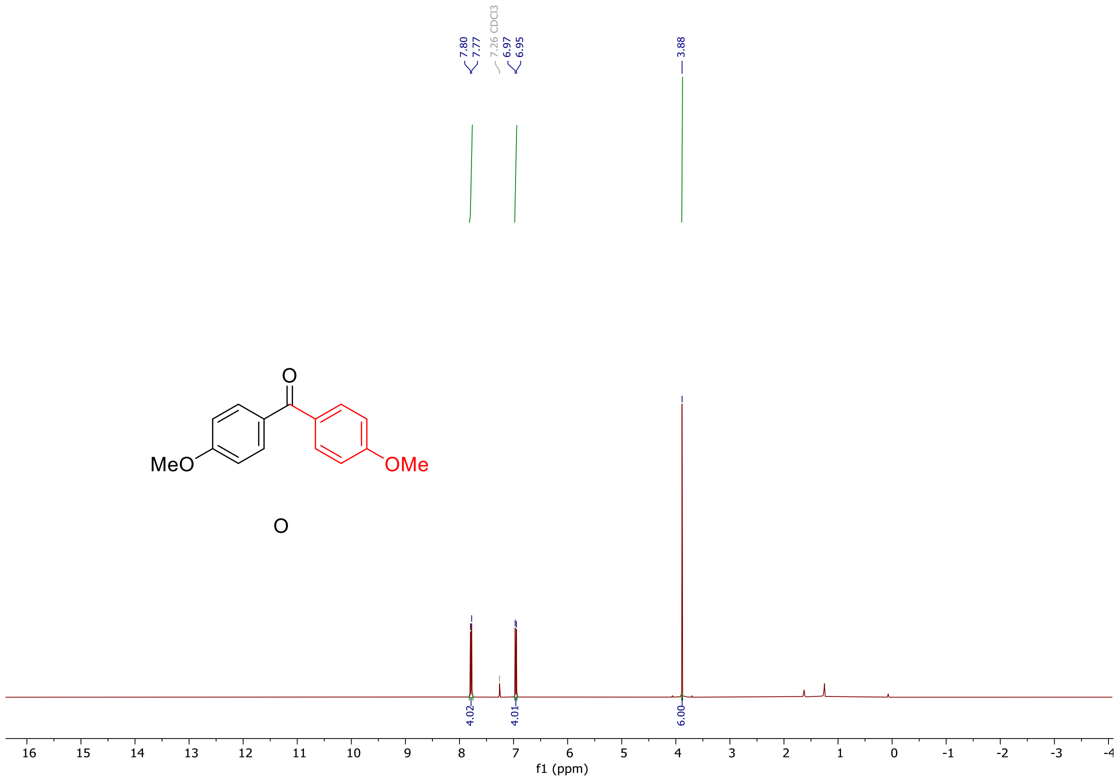

Figure S24. ^{1}H NMR of bis(4-methoxyphenyl)methanone

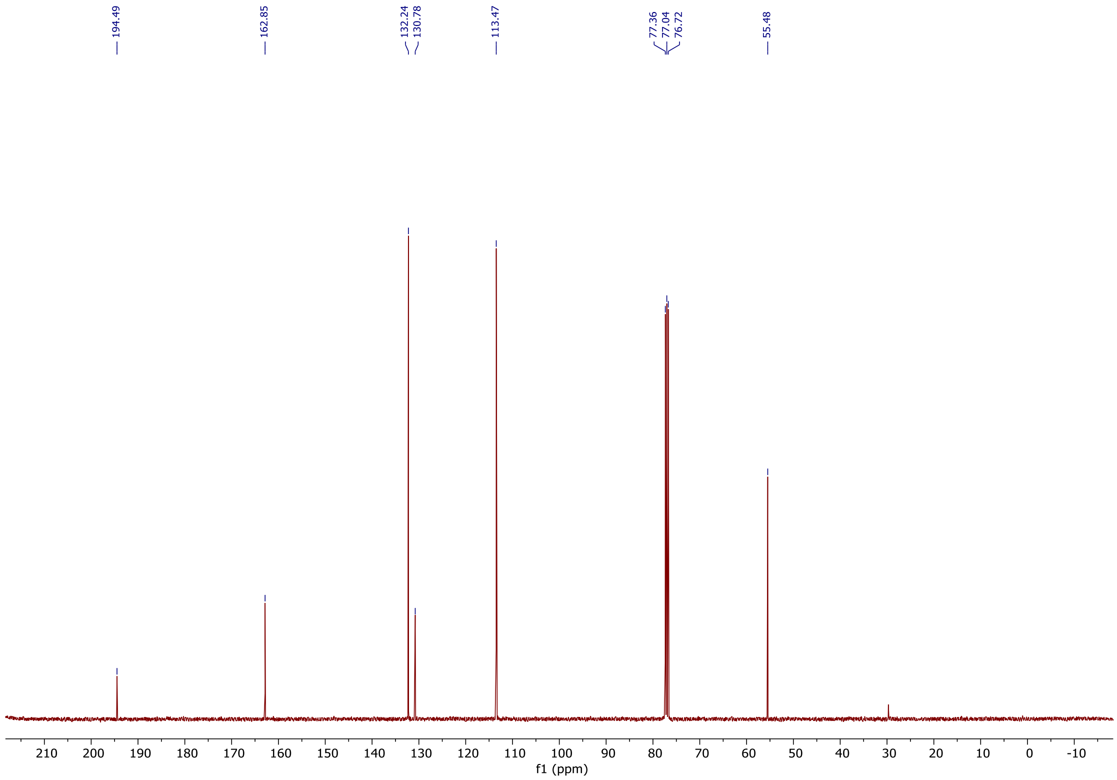

Figure S25. ^{13}C NMR of bis(4-methoxyphenyl)methanone

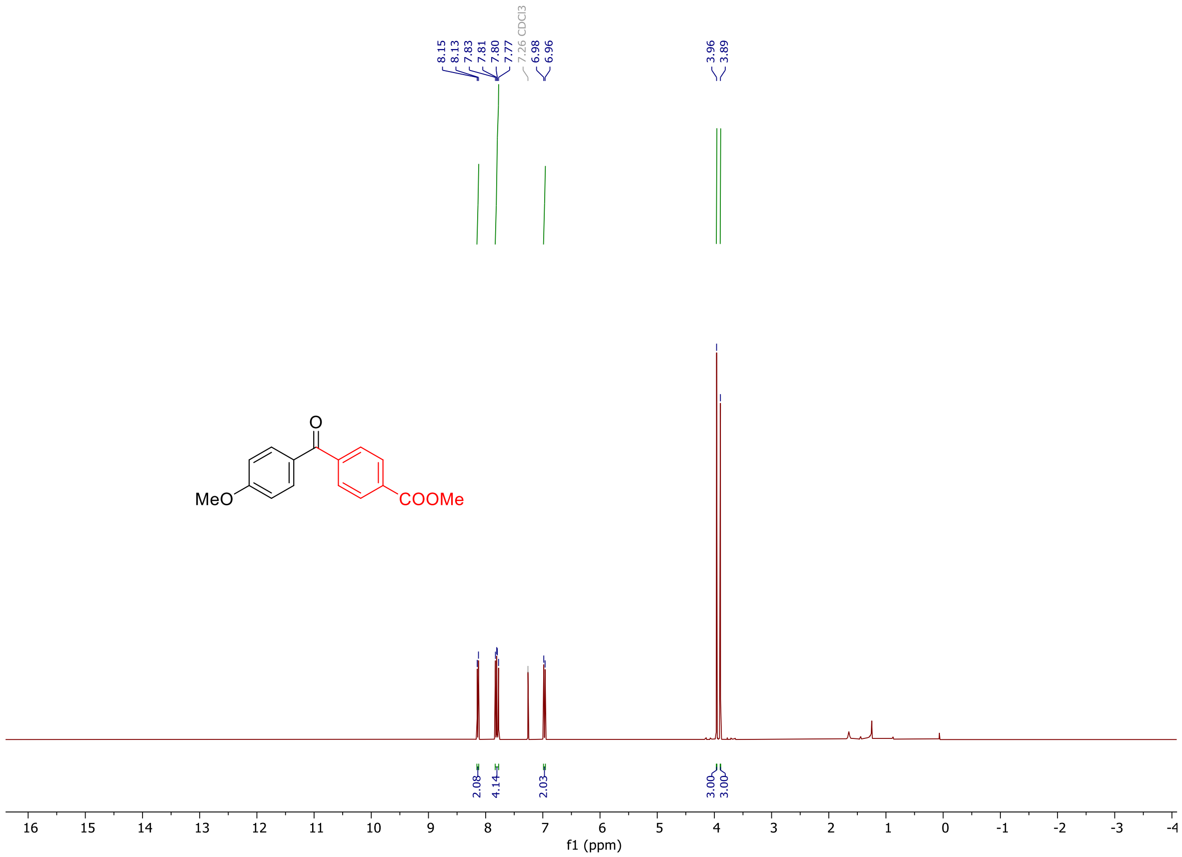

Figure S26. ^{1}H NMR of methyl-4(4-methoxybenzoyl)benzoate

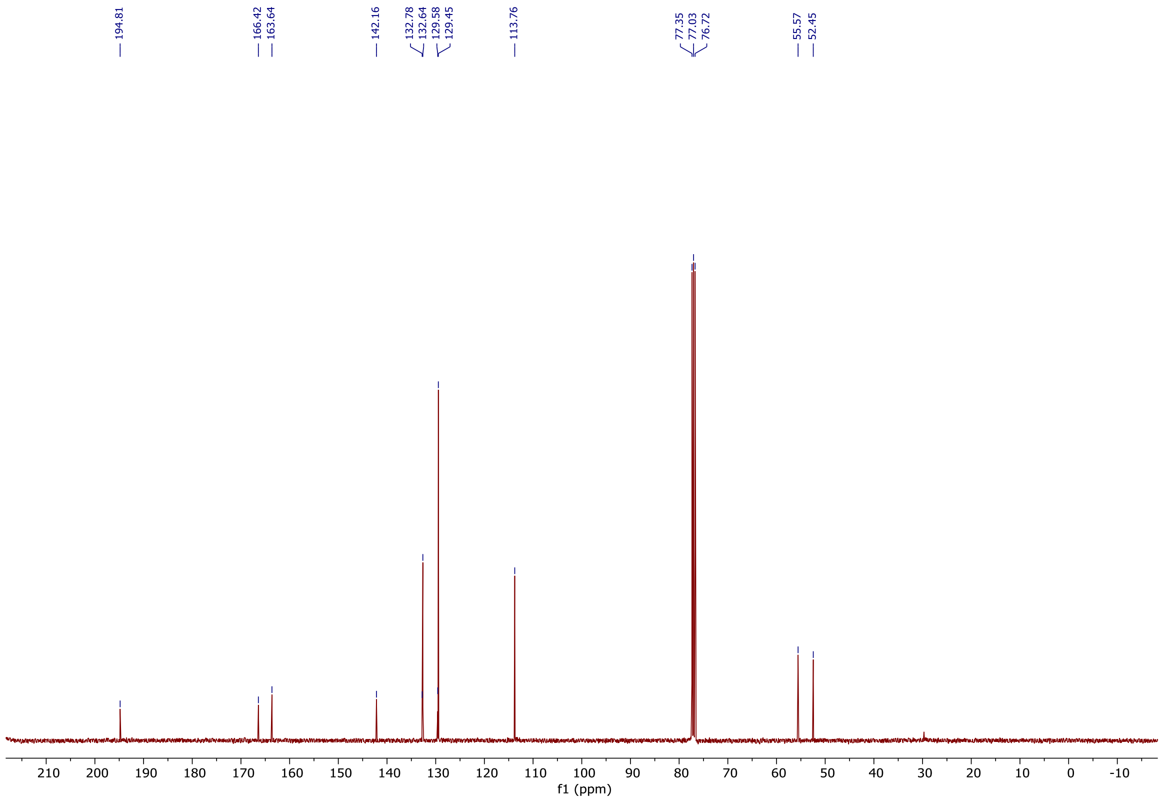

Figure S27. ^{13}C NMR of methyl-4(4-methoxybenzoyl)benzoate

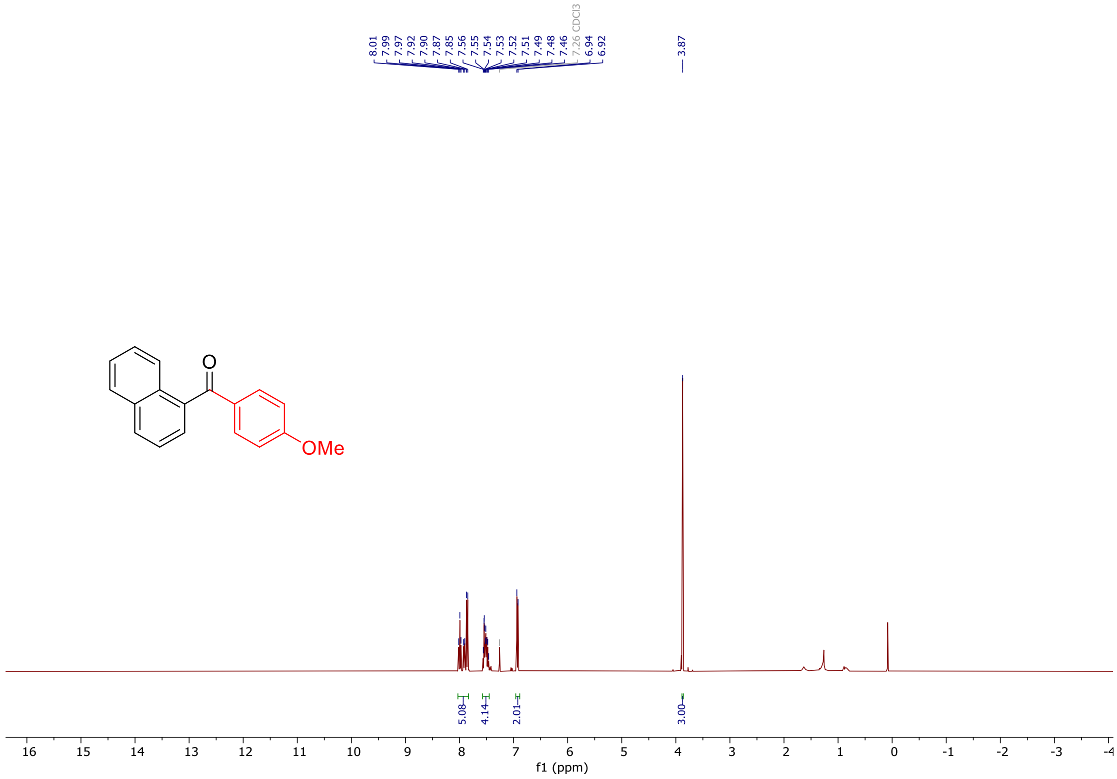

Figure S28. ^{1}H NMR of 4-methoxyphenyl(naphthalen-1-yl)methanone

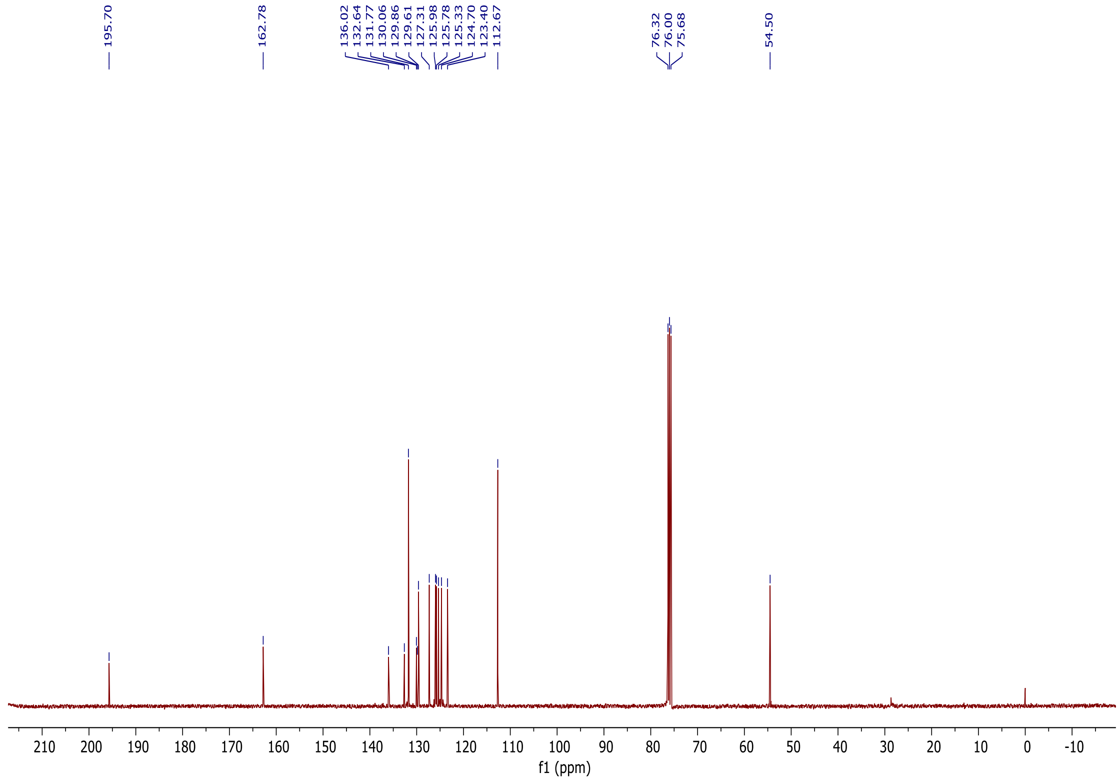

Figure S29. ^{13}C NMR of 4-methoxyphenyl(naphthalen-1-yl)methanone

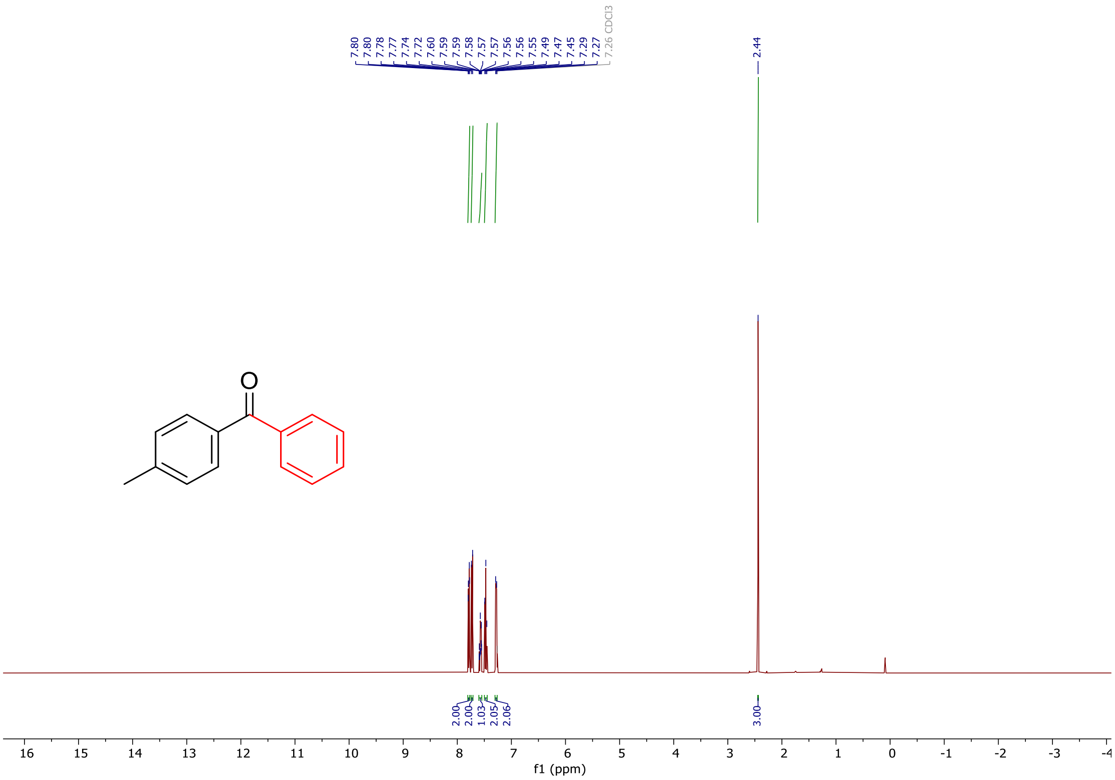

Figure S30. ^{1}H NMR of phenyl(p-tolyl)methanone

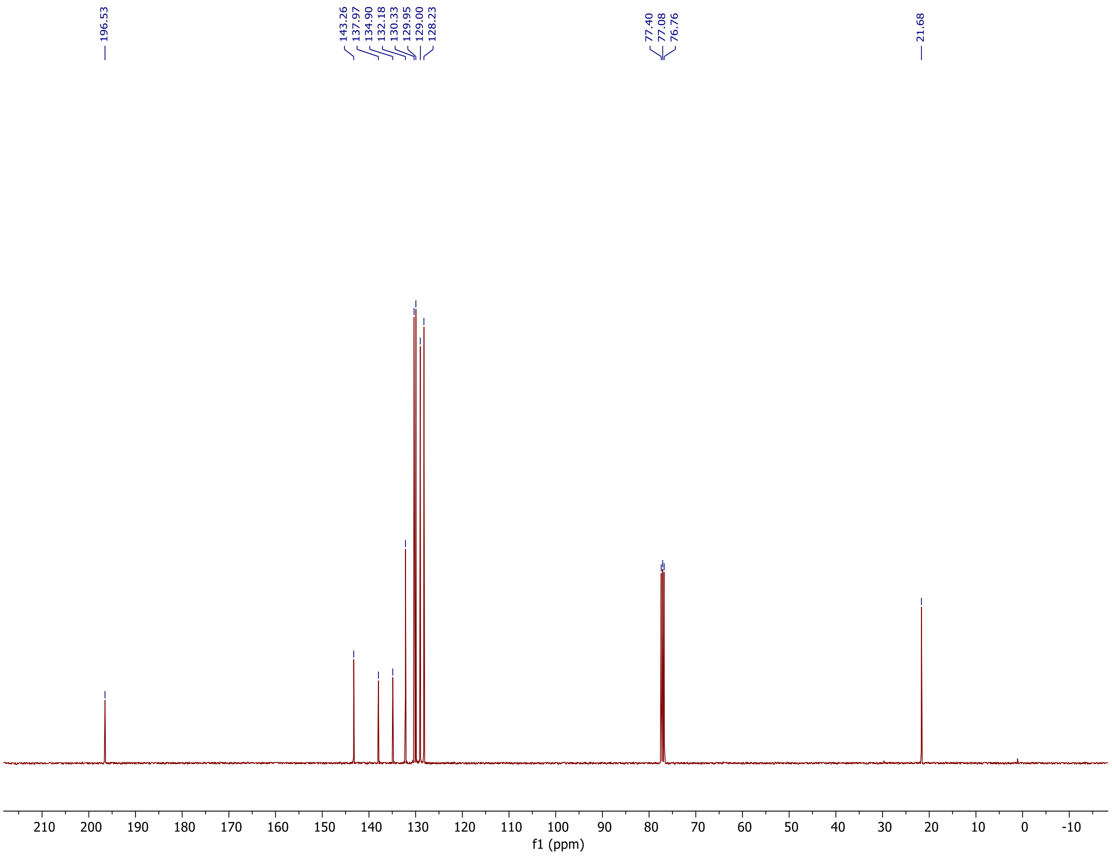

Figure S31. ^{13}C NMR of phenyl(p-tolyl)methanone

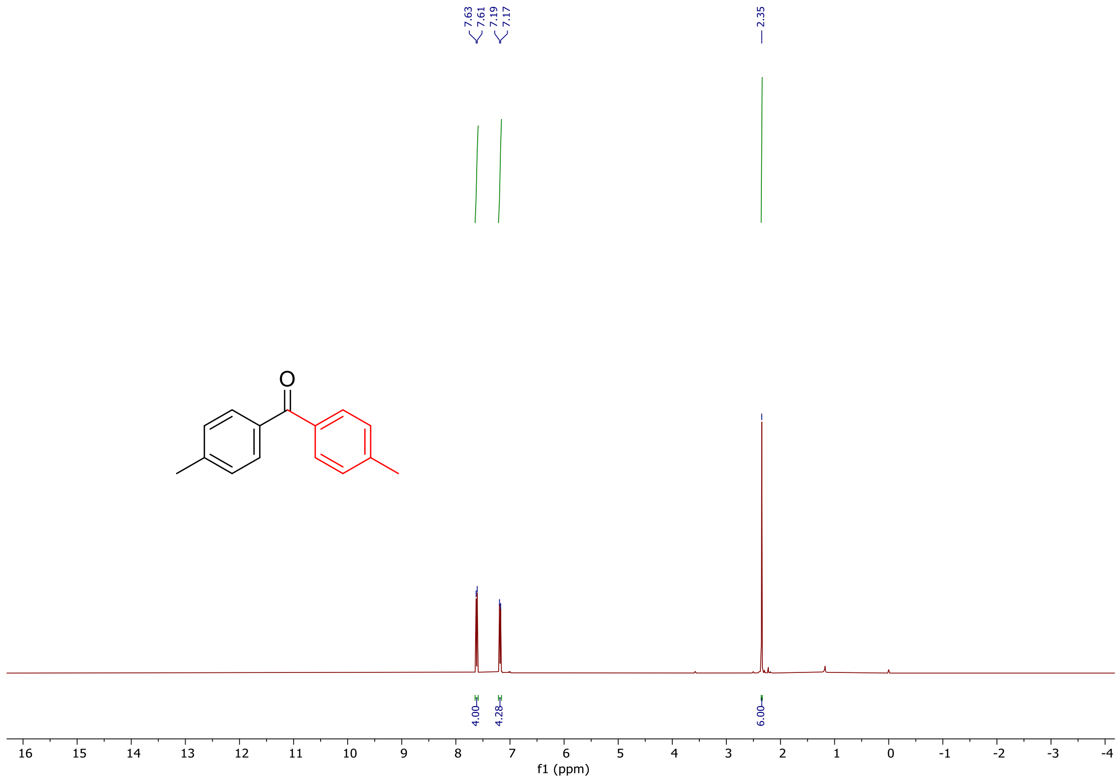

Figure S32. ^{1}H NMR of di-(p-tolyl)methanone

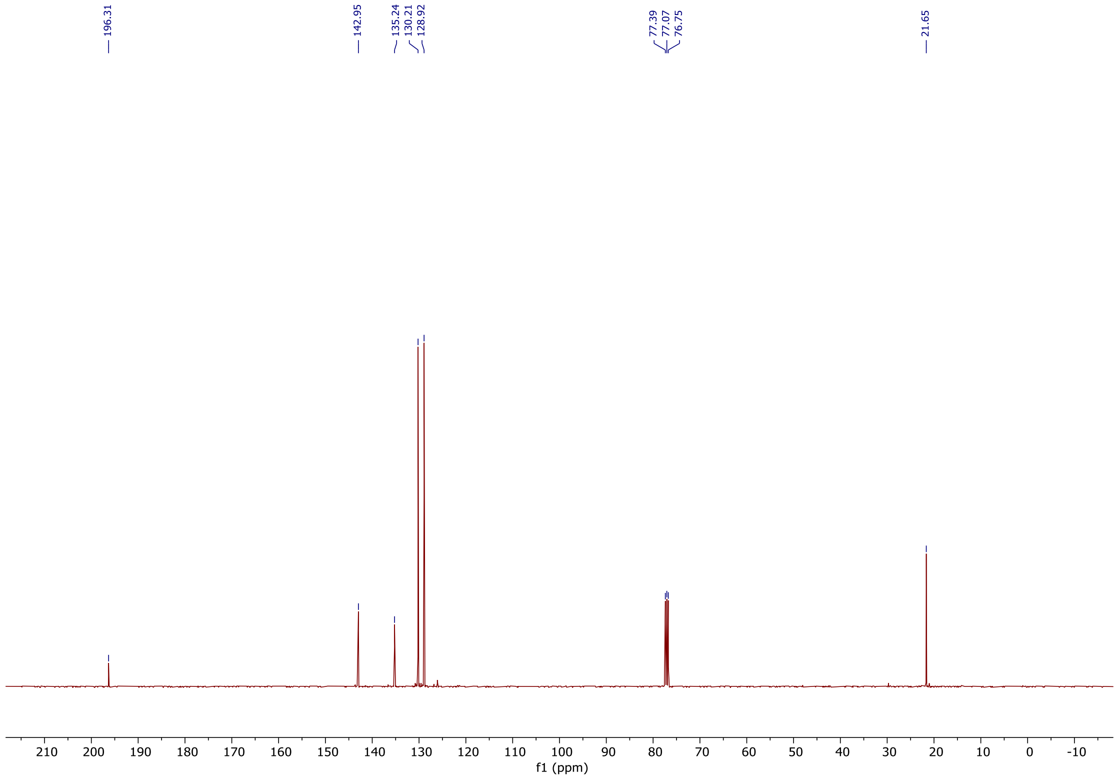

Figure S33. ^{13}C NMR of di-(p-tolyl)methanone

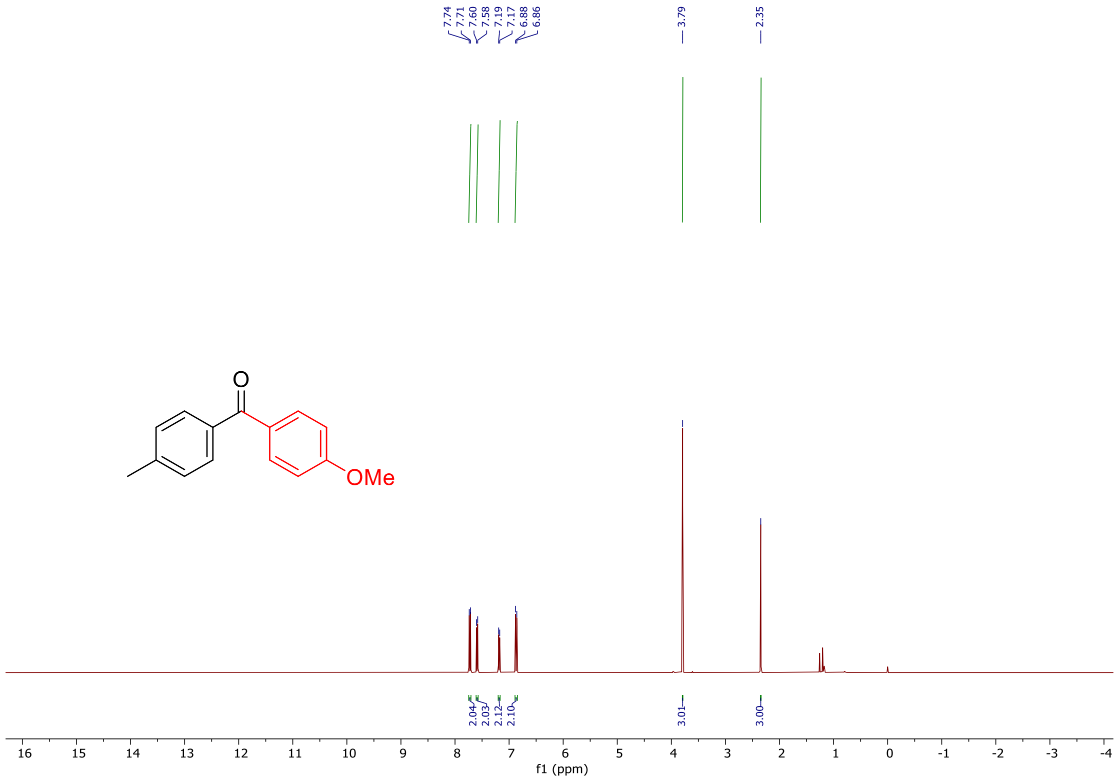

Figure S34. ^{1}H NMR of 4-methoxyphenyl(p-tolyl)methanone

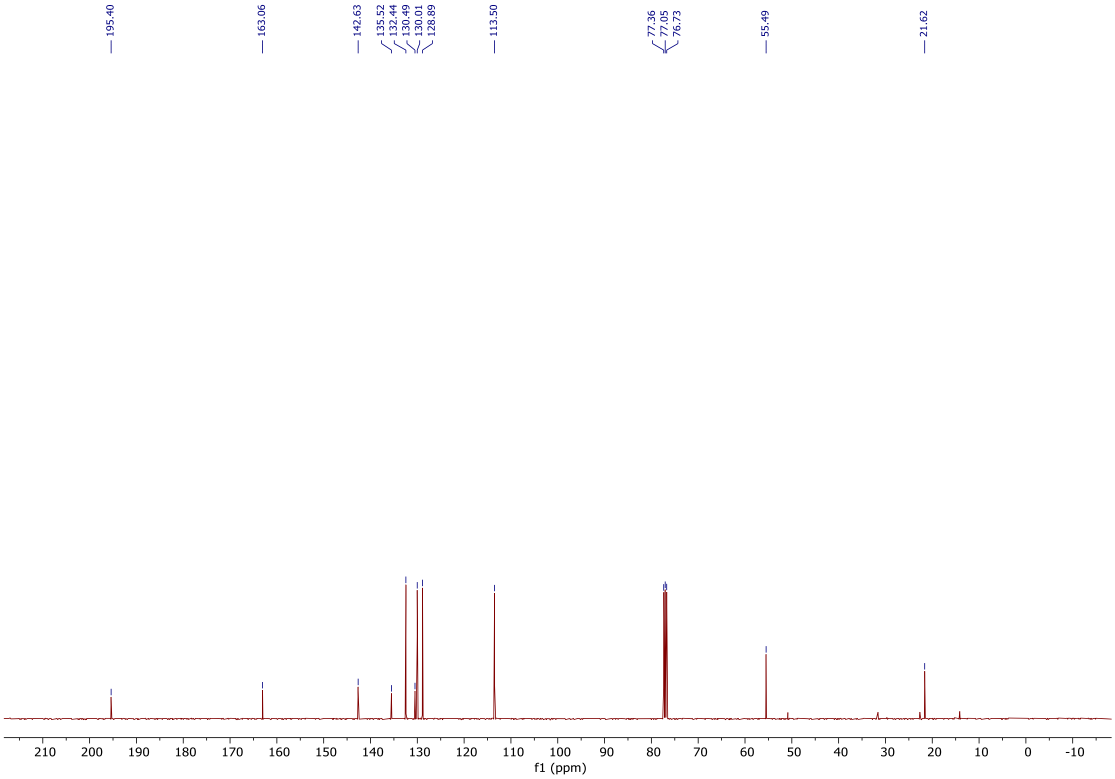

Figure S35. ^{13}C NMR of 4-methoxyphenyl(p-tolyl)methanone

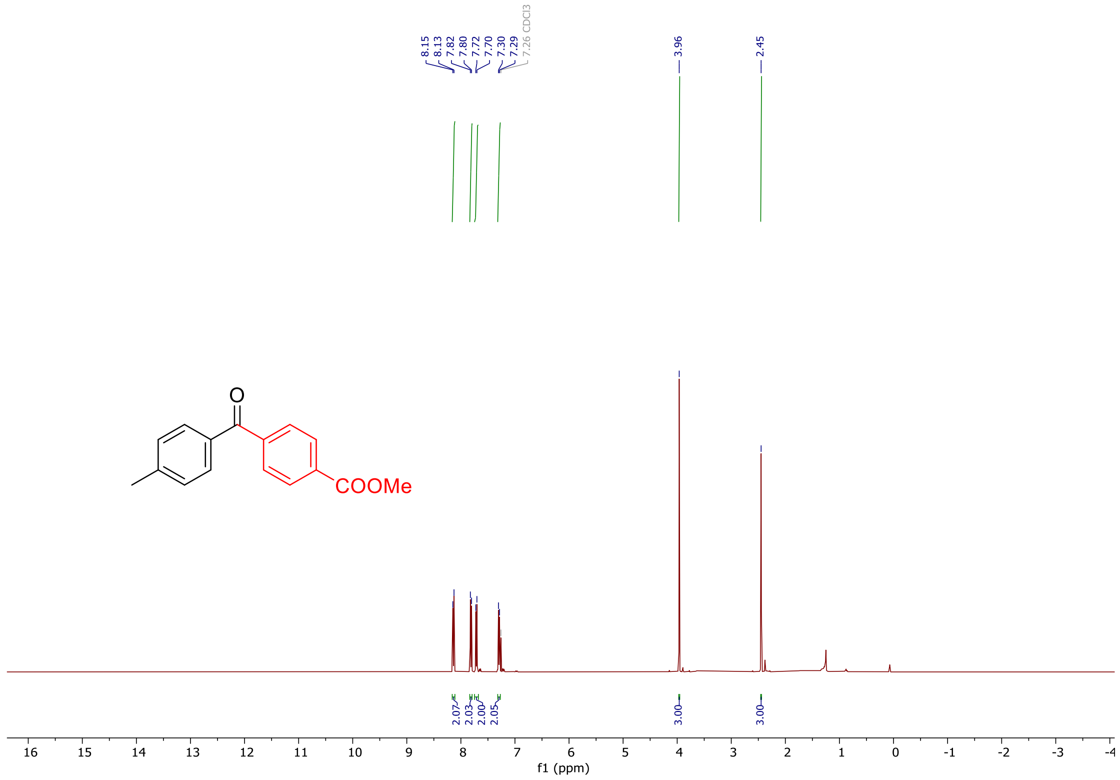

Figure S36. ^{1}H NMR of methyl-4(4-methylbenzoyl)benzoate

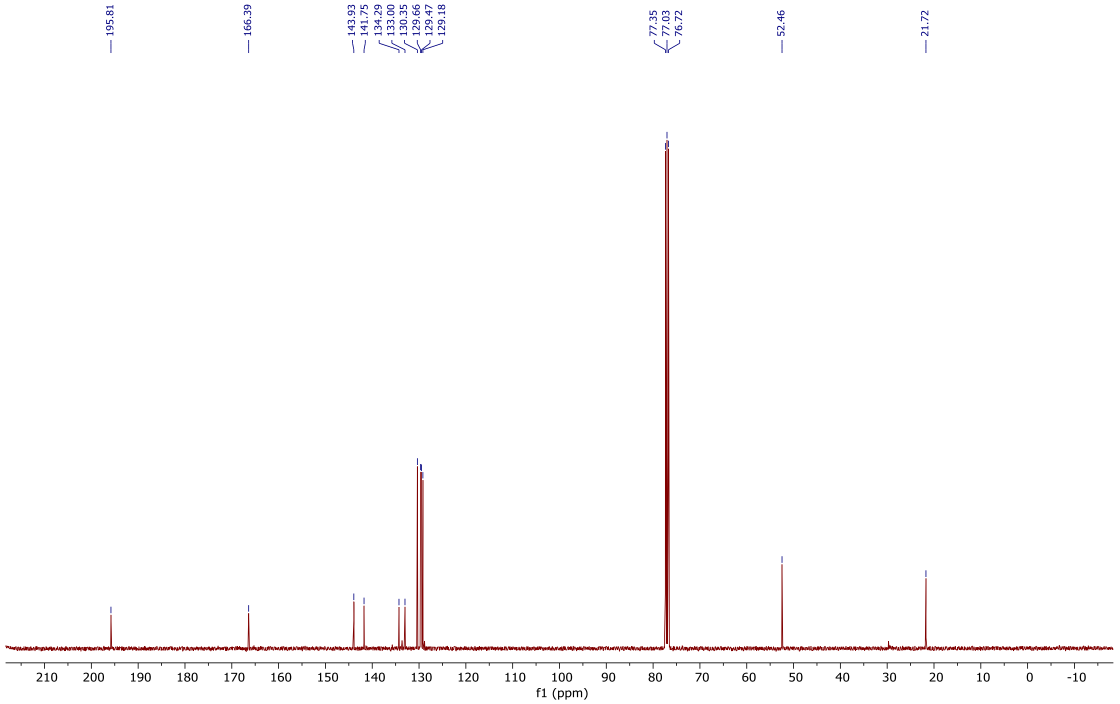

Figure S37. ^{13}C NMR of methyl-4(4-methylbenzoyl)benzoate

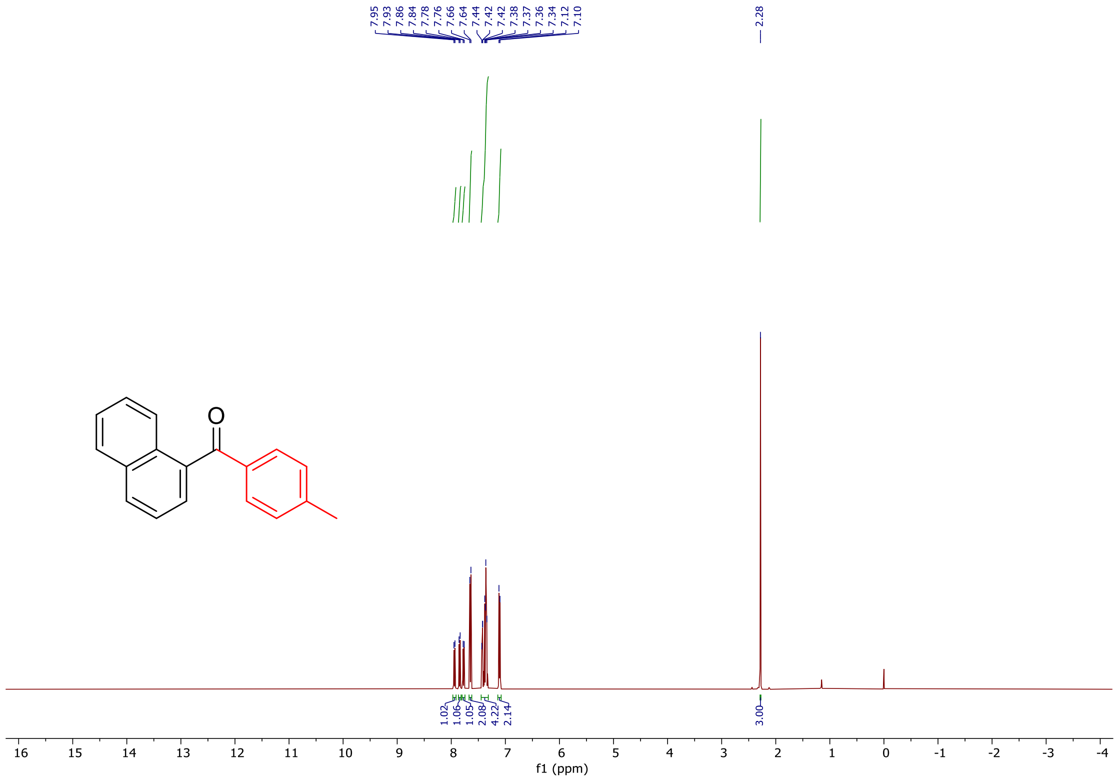

Figure S38. ¹H NMR of naphthalen-1-yl(p-tolyl)mathanone

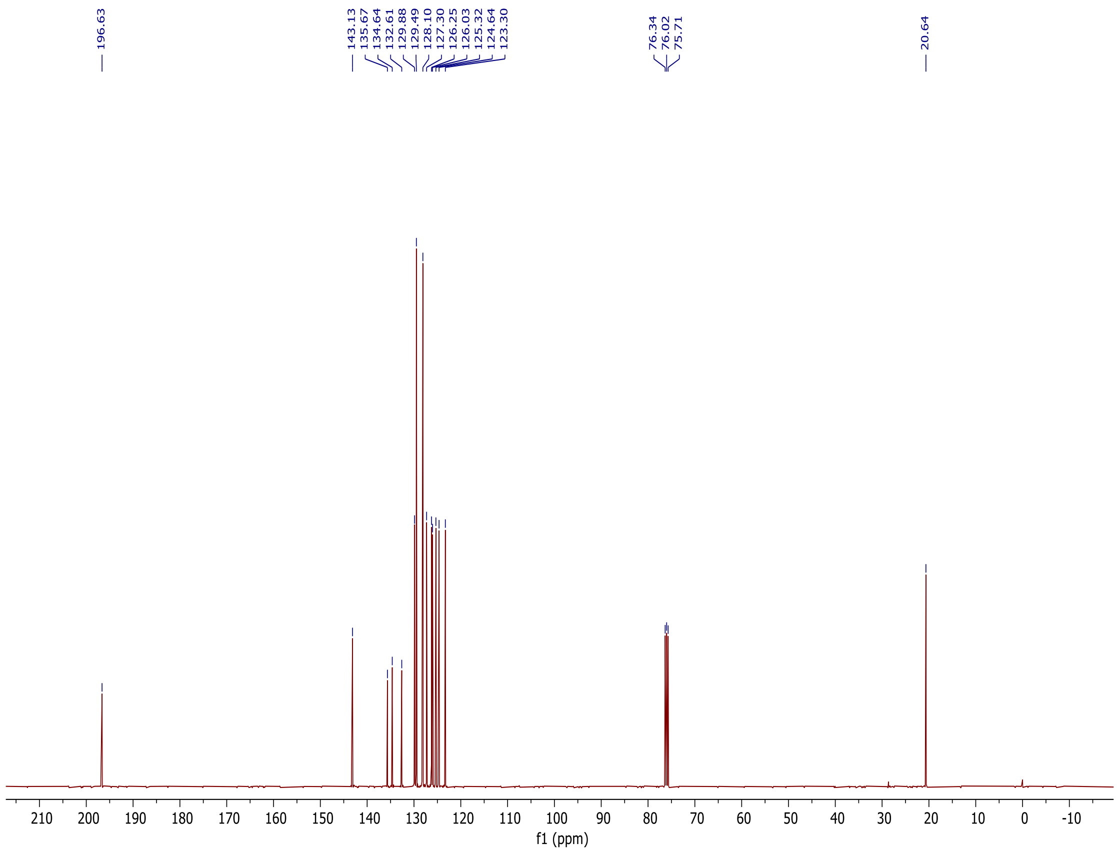

Figure S3. ¹³C NMR of naphthalen-1-yl(p-tolyl)mathanone

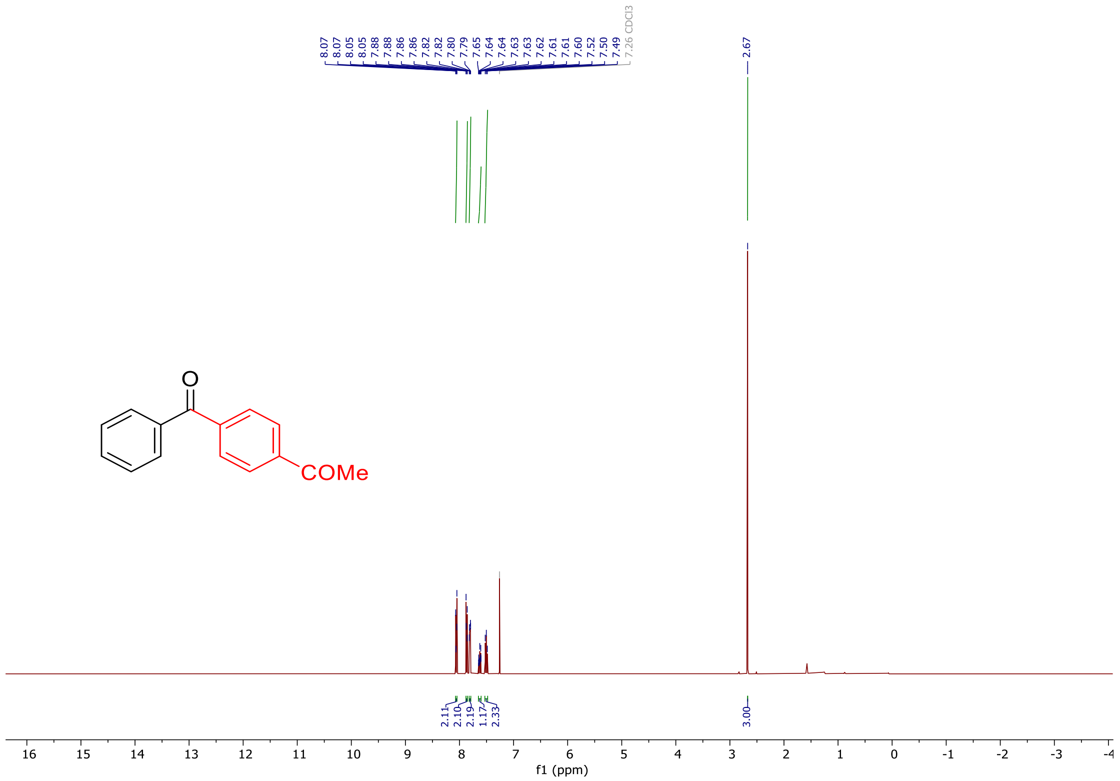

Figure S40. ^{1}H NMR of 1-(4-benzoylphenyl)ethan-1-one

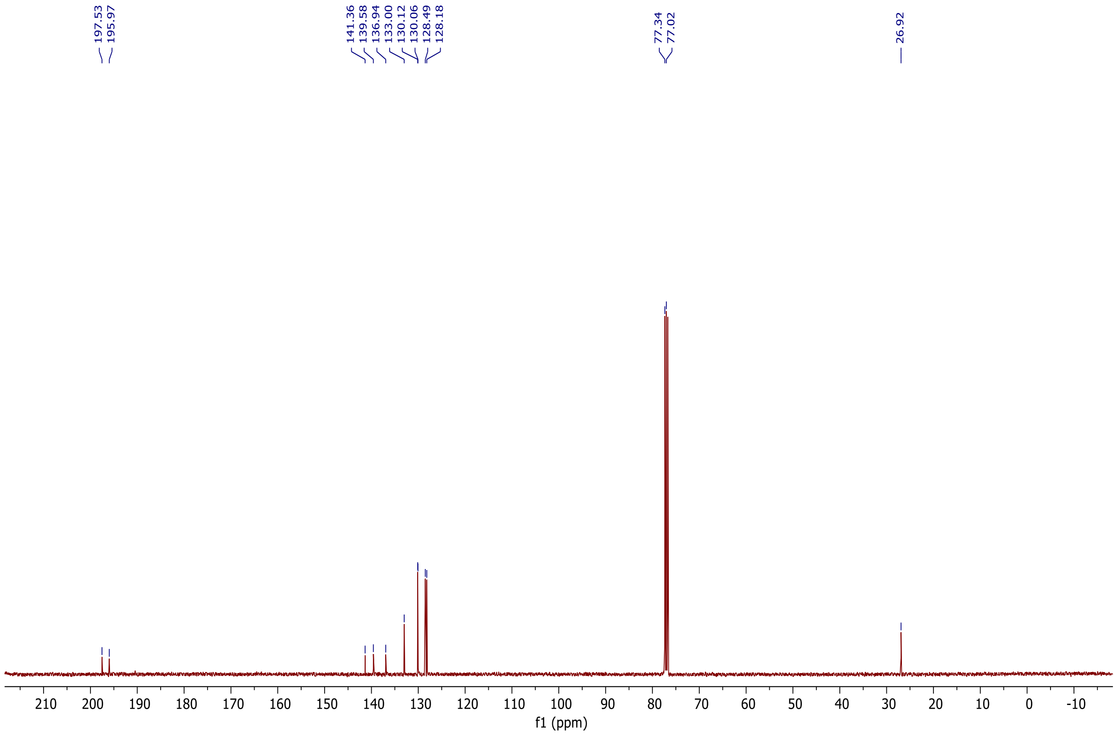

Figure S41. ^{13}C NMR of 1-(4-benzoylphenyl)ethan-1-one

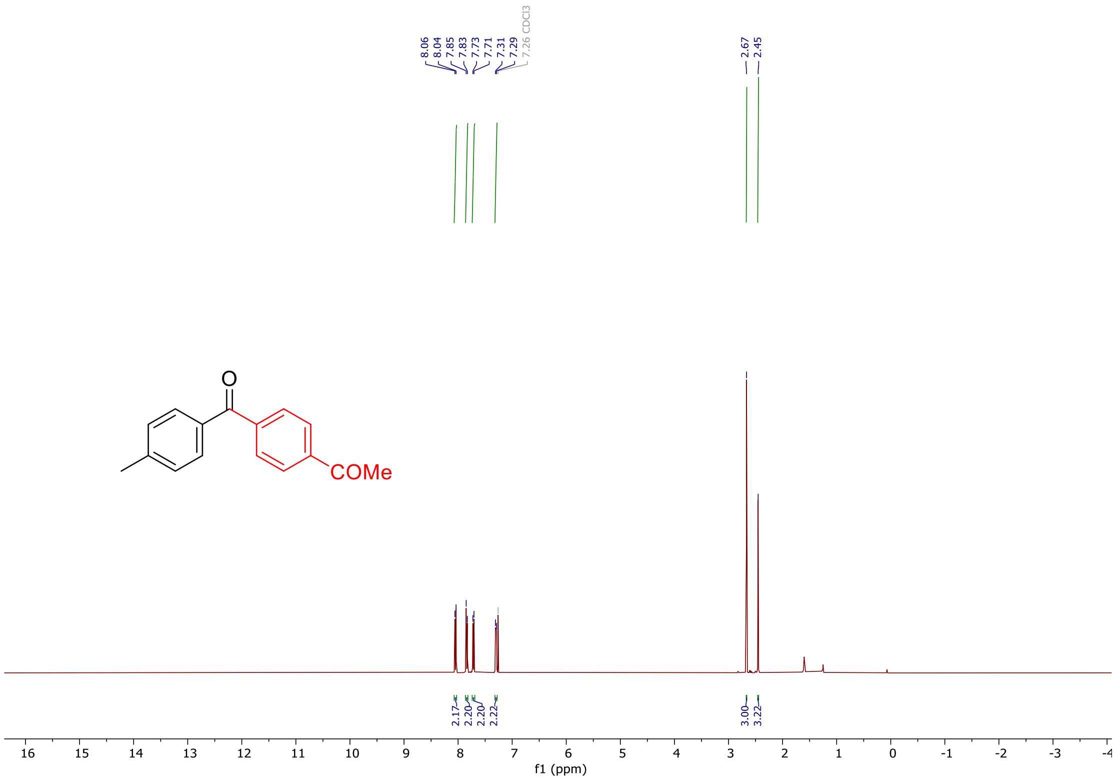

Figure S42. ^{1}H NMR of 1-(4-(4-methylbezoyl)phenyl)ethan-1-one

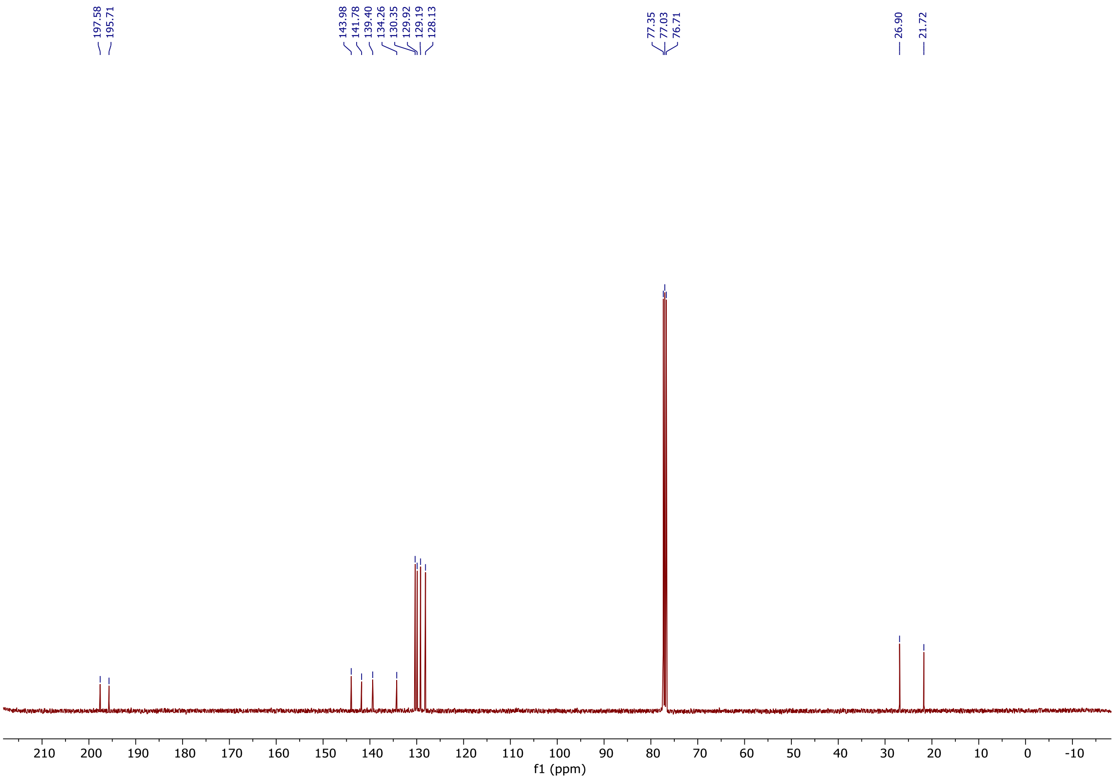

Figure S43. ^{13}C NMR of 1-(4-(4-methylbezoyl)phenyl)ethan-1-one

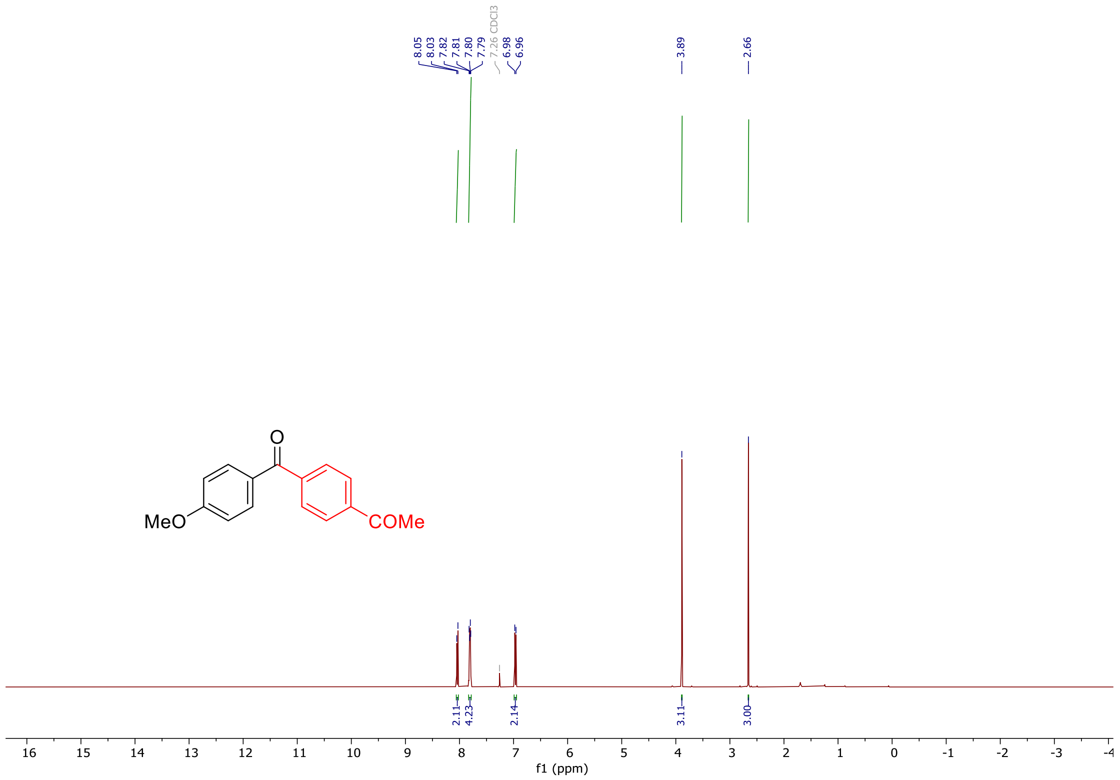

Figure S44. ^{1}H NMR of 1-(4-(4-methoxybezoyl)phenyl)ethan-1-one

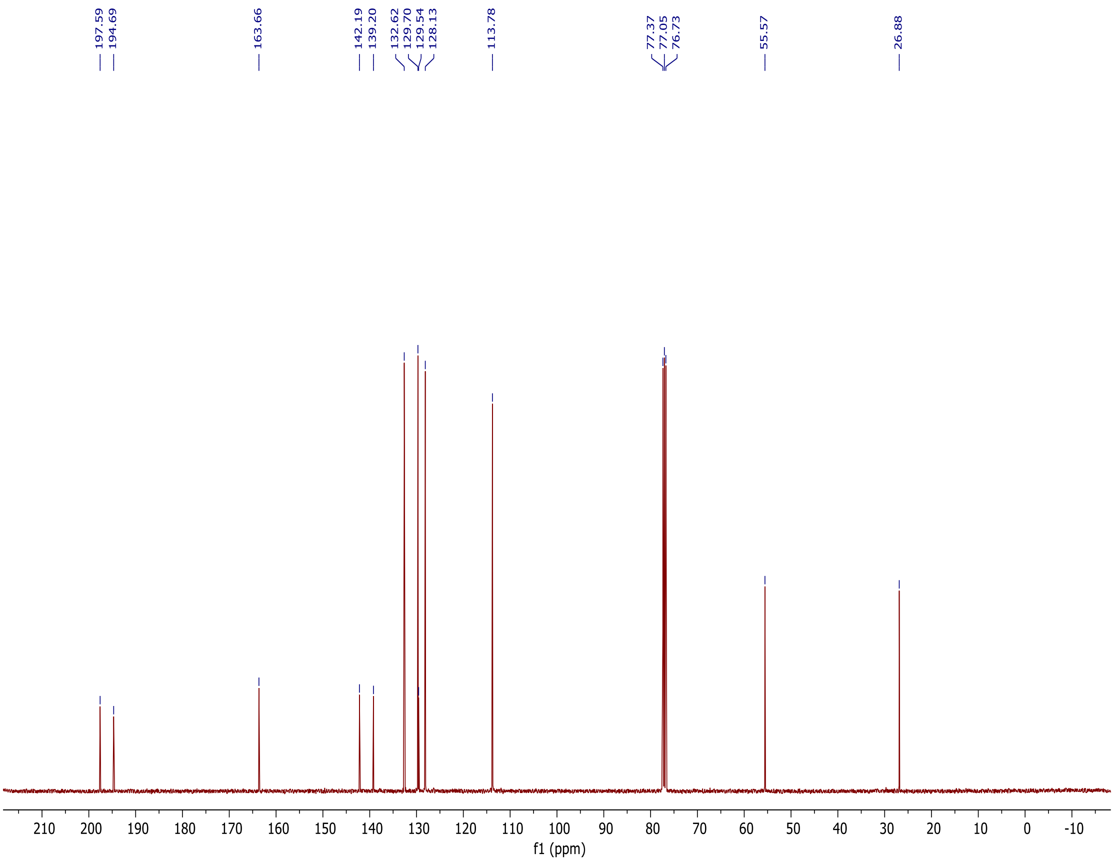

Figure S45. ^{13}C NMR of 1-(4-(4-methoxybezoyl)phenyl)ethan-1-one

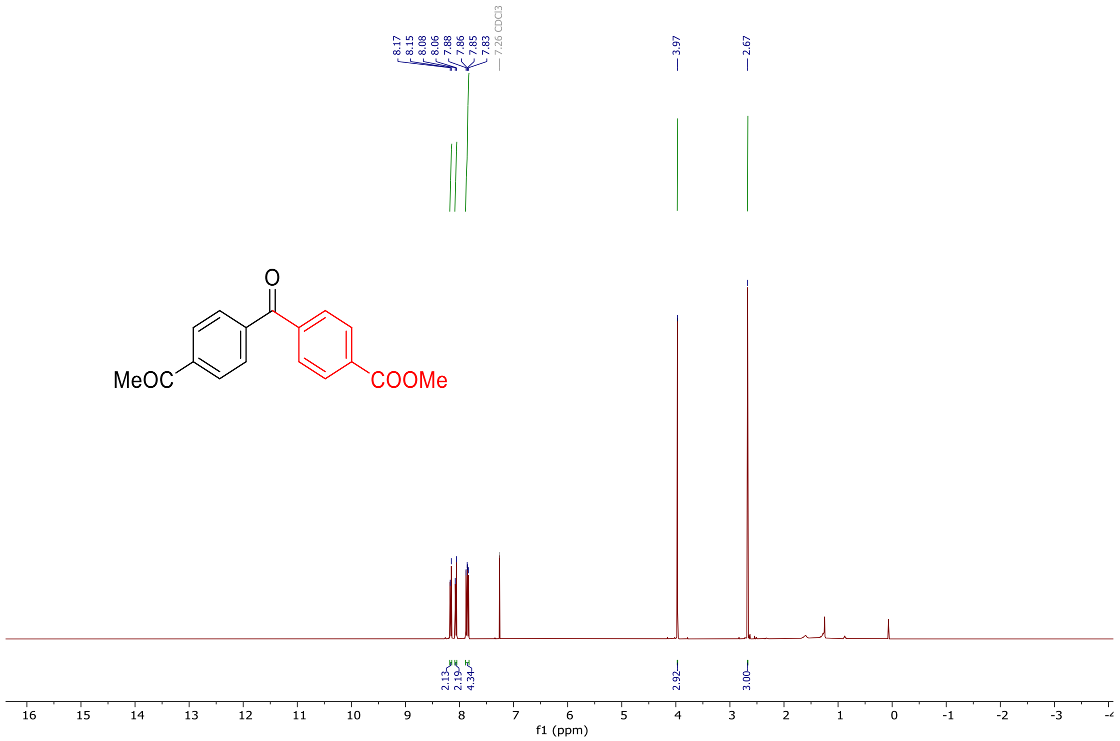

Figure S46.^{1}H NMR of methyl-4-(4-acetylbenzoyl)benzoate

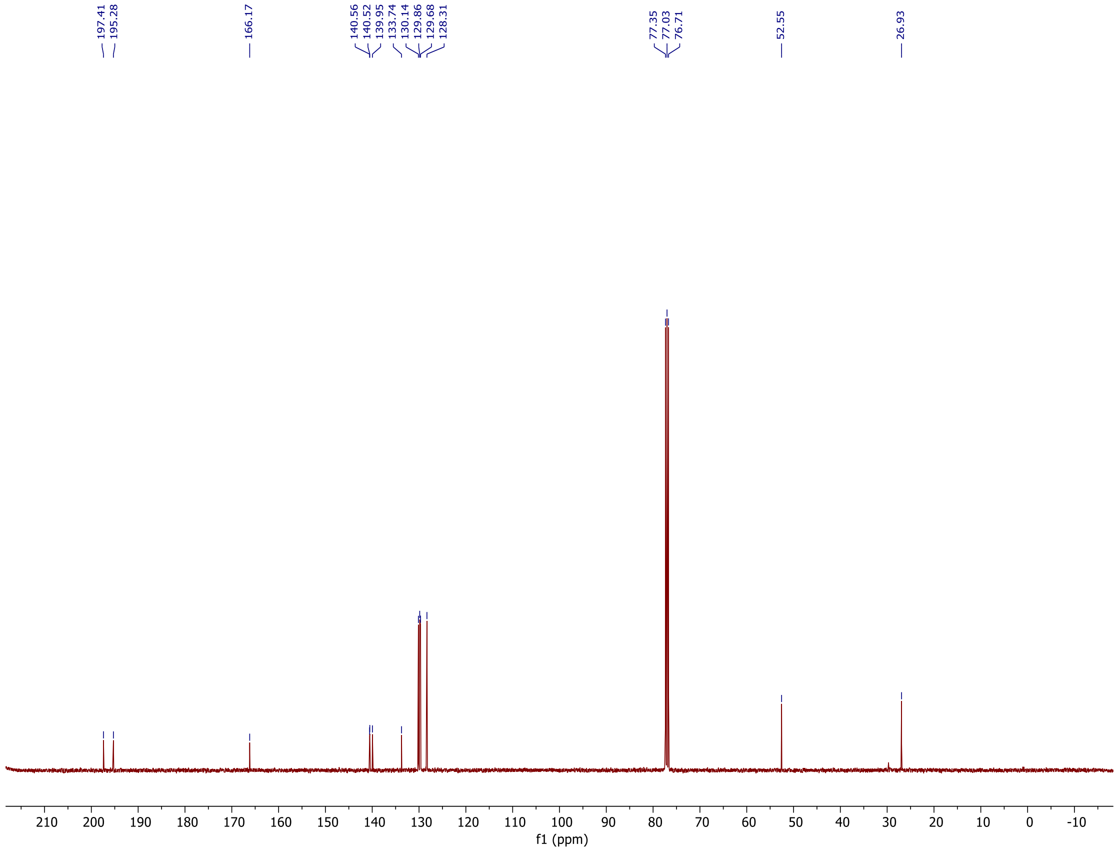

Figure S47. ^{13}C NMR of methyl-4-(4-acetylbenzoyl)benzoate

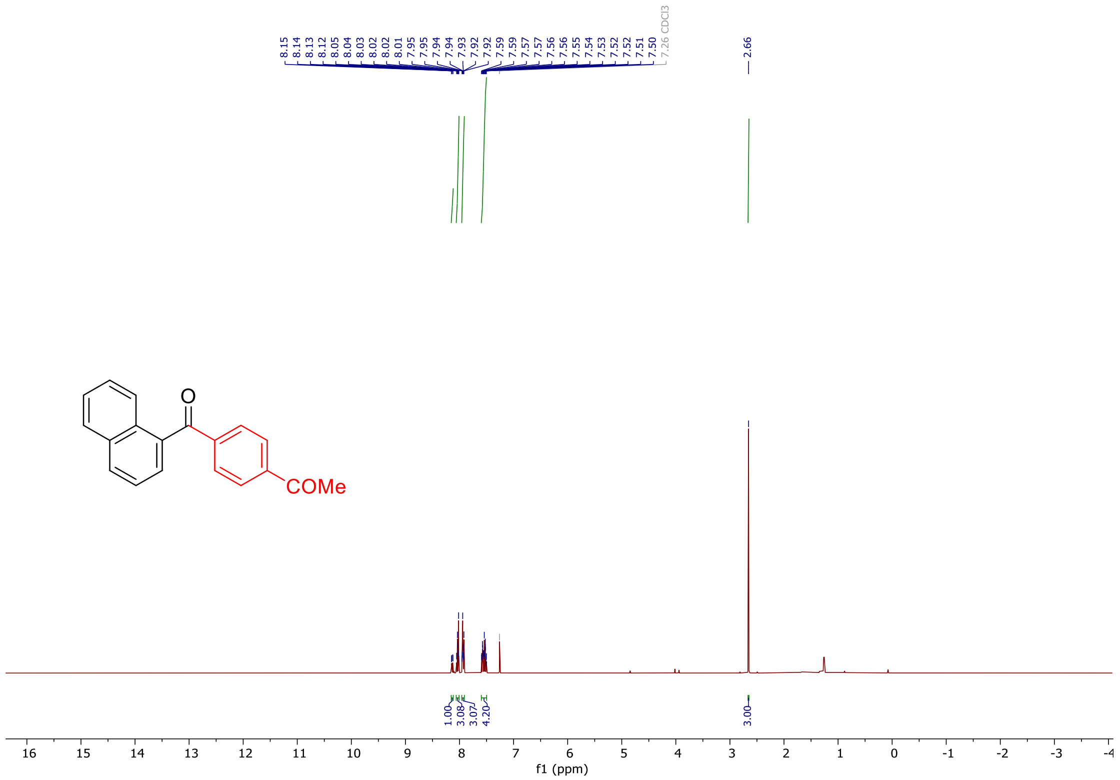

Figure S4. ^{1}H NMR of 1-(4-(1-napthoyl)phenyl)ethan-1-one

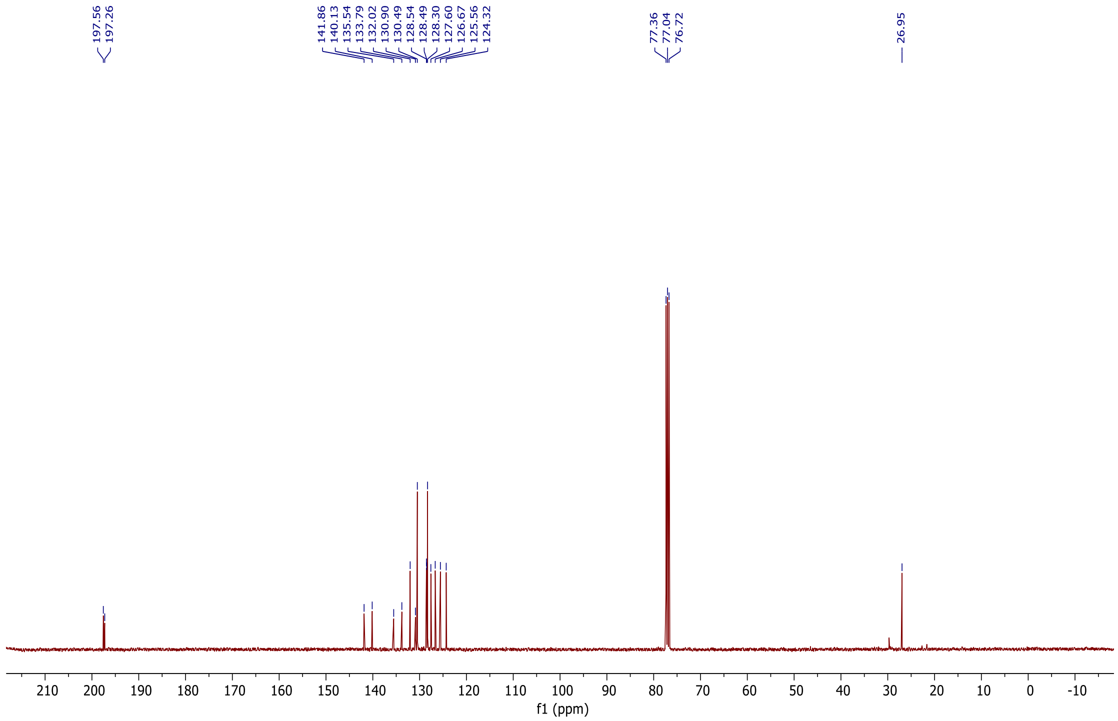

Figure S5. ^{13}C NMR of 1-(4-(1-napthoyl)phenyl)ethan-1-one

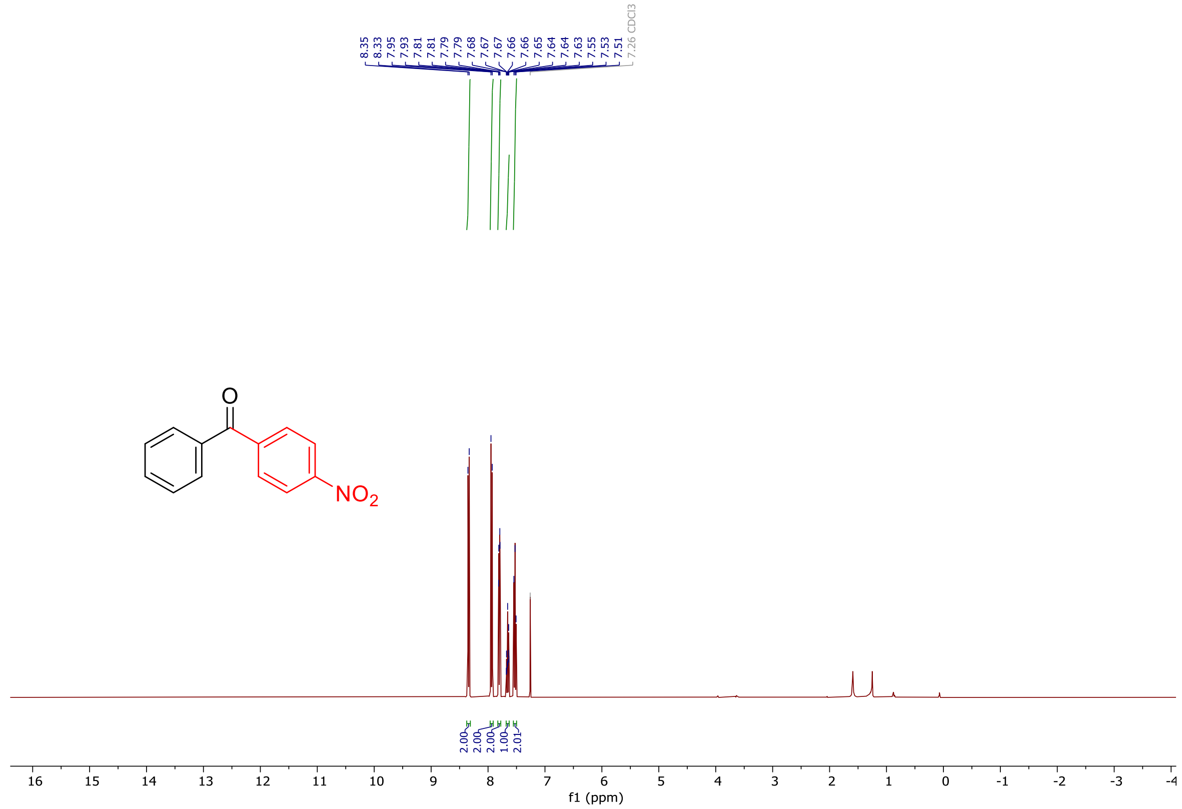

Figure S50. ^{1}H NMR of 4-nitrophenyl(phenyl)mathanone

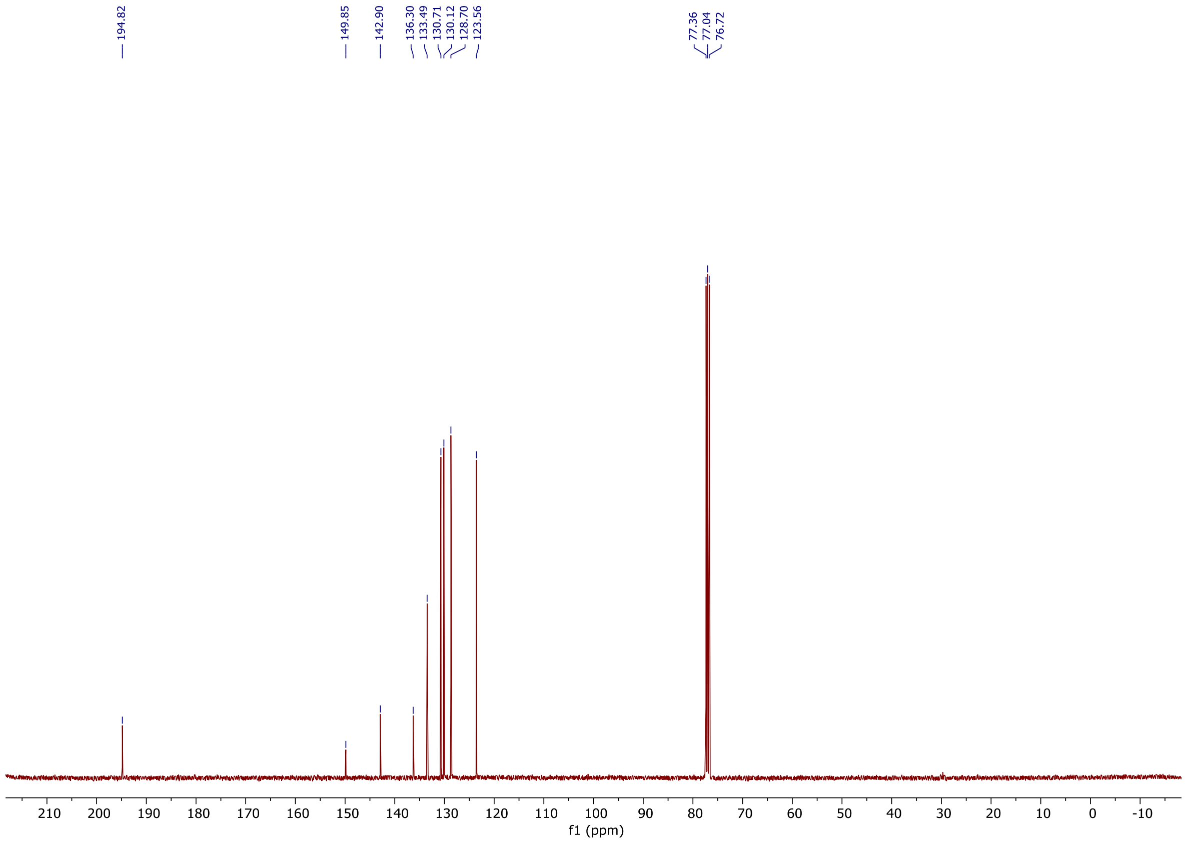

Figure S51. ^{13}C NMR of 4-nitrophenyl(phenyl)mathanone

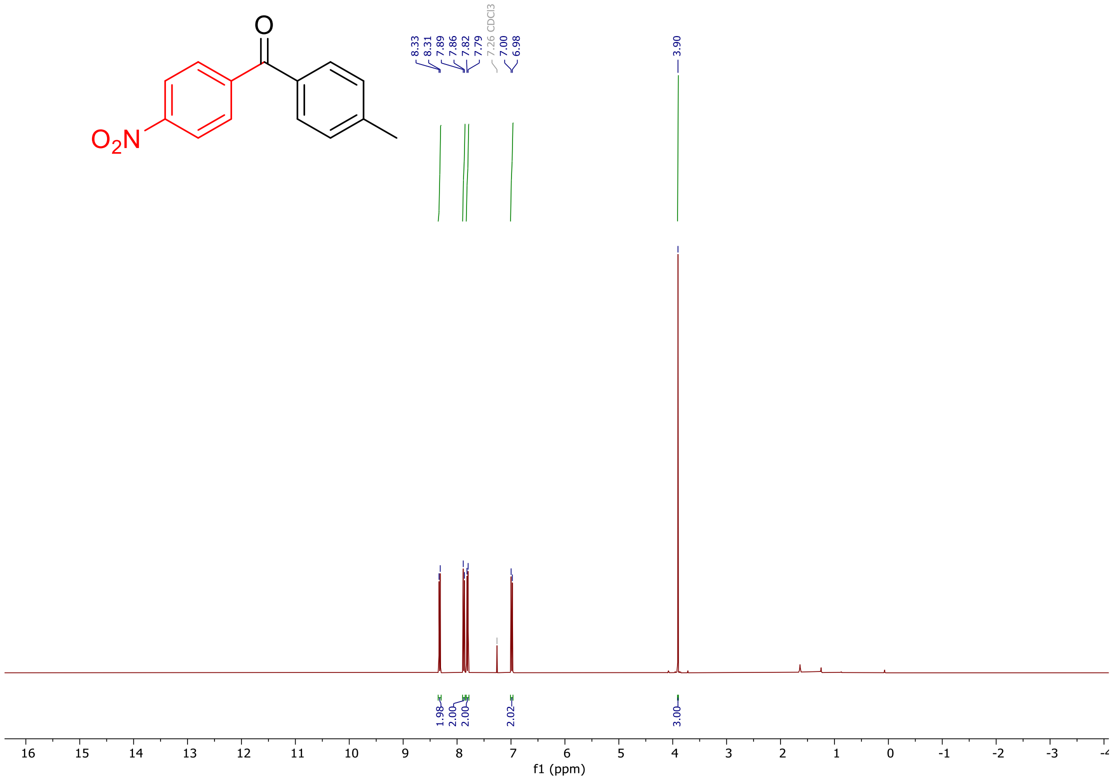

Figure S52. ^{1}H NMR of 4-nitrophenyl(p-tolyl)mathanone

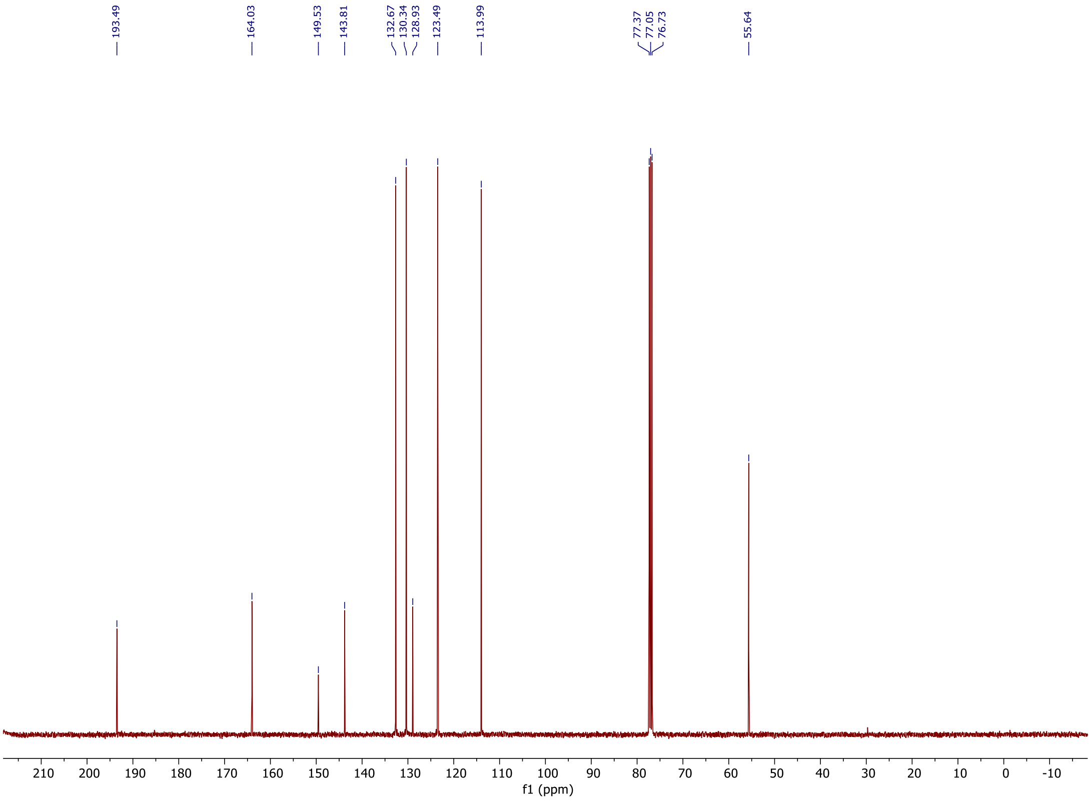

Figure S53. ^{13}C NMR of 4-nitrophenyl(p-tolyl)mathanone

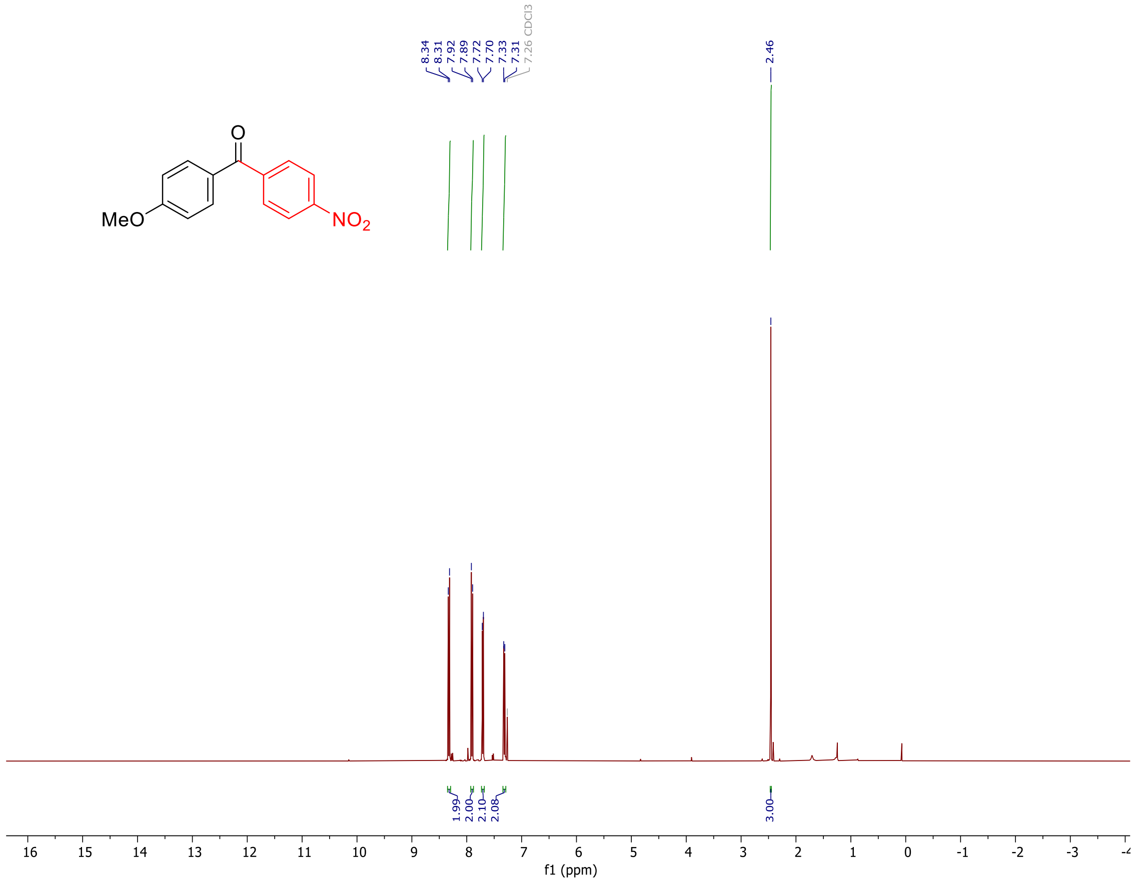

Figure S54. ^{1}H NMR of 4-methoxyphenyl(4-nitrophenyl)mathanone

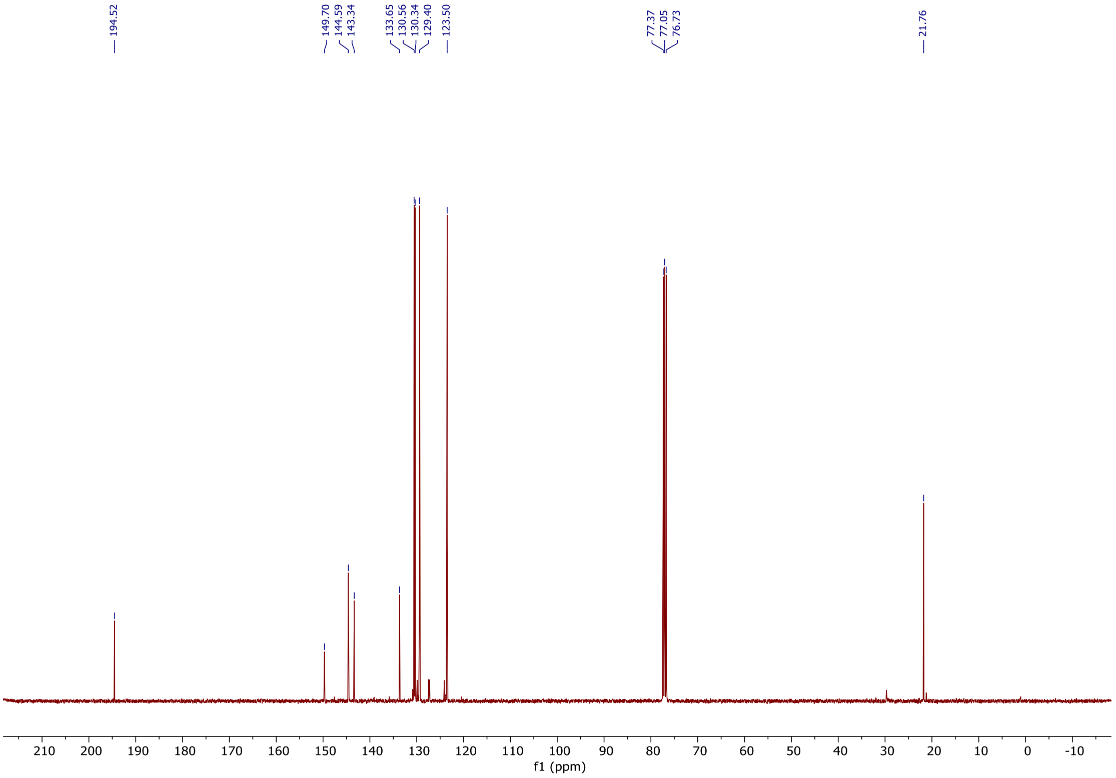

Figure S55. ^{13}C NMR of 4-methoxyphenyl(4-nitrophenyl)mathanone

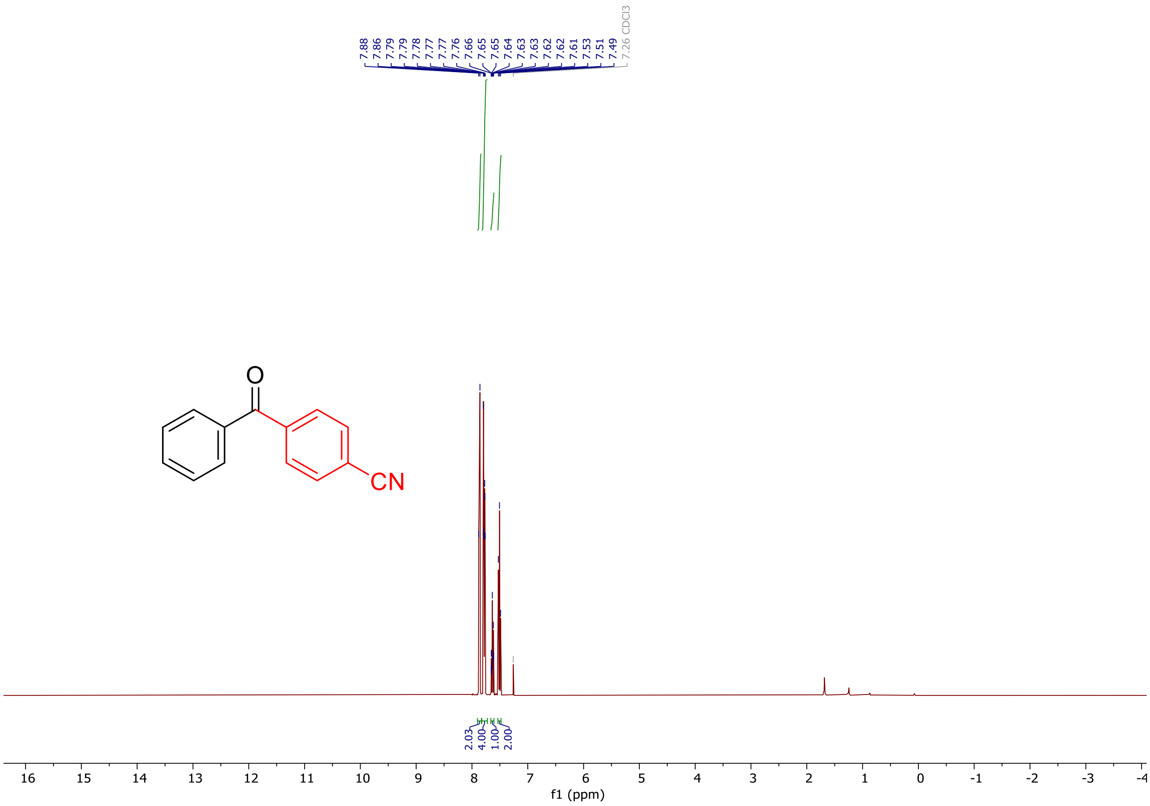

Figure S56. ^{1}H NMR of 4-benzoylbenzonitrile

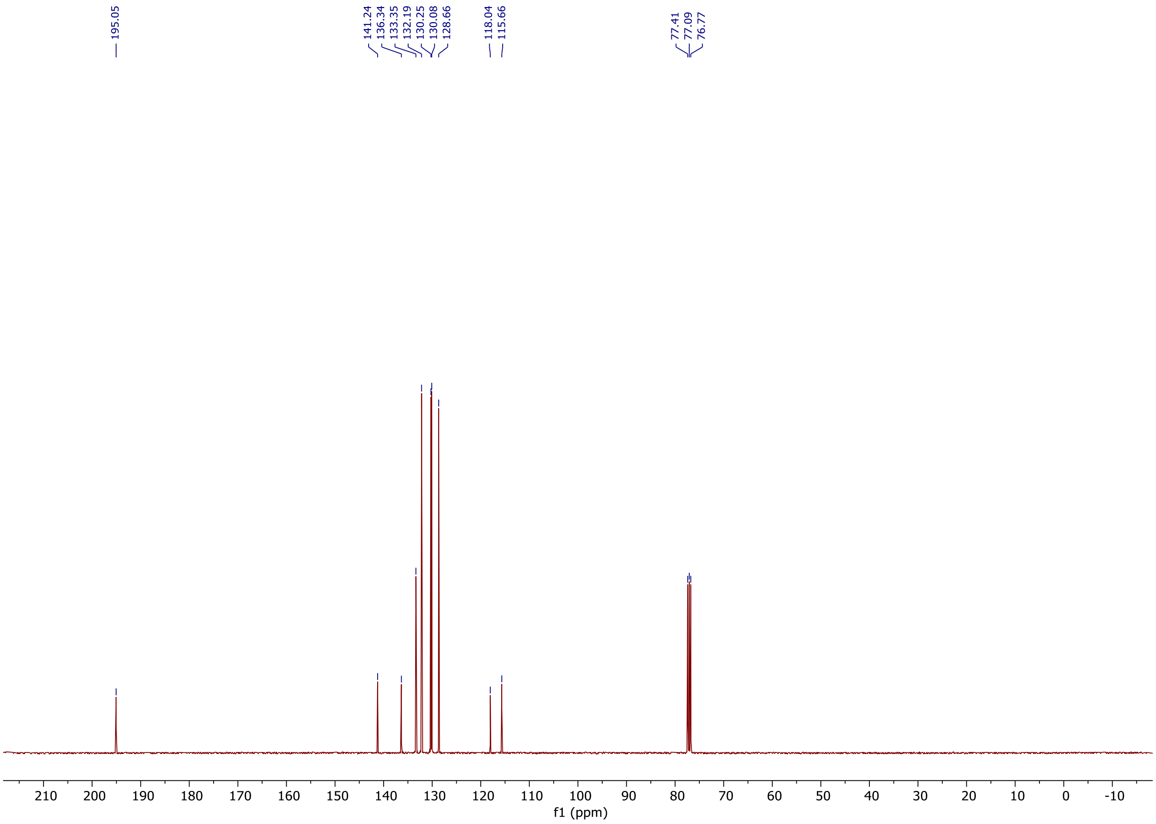

Figure S57. ^{13}C NMR of 4-benzoylbenzonitrile

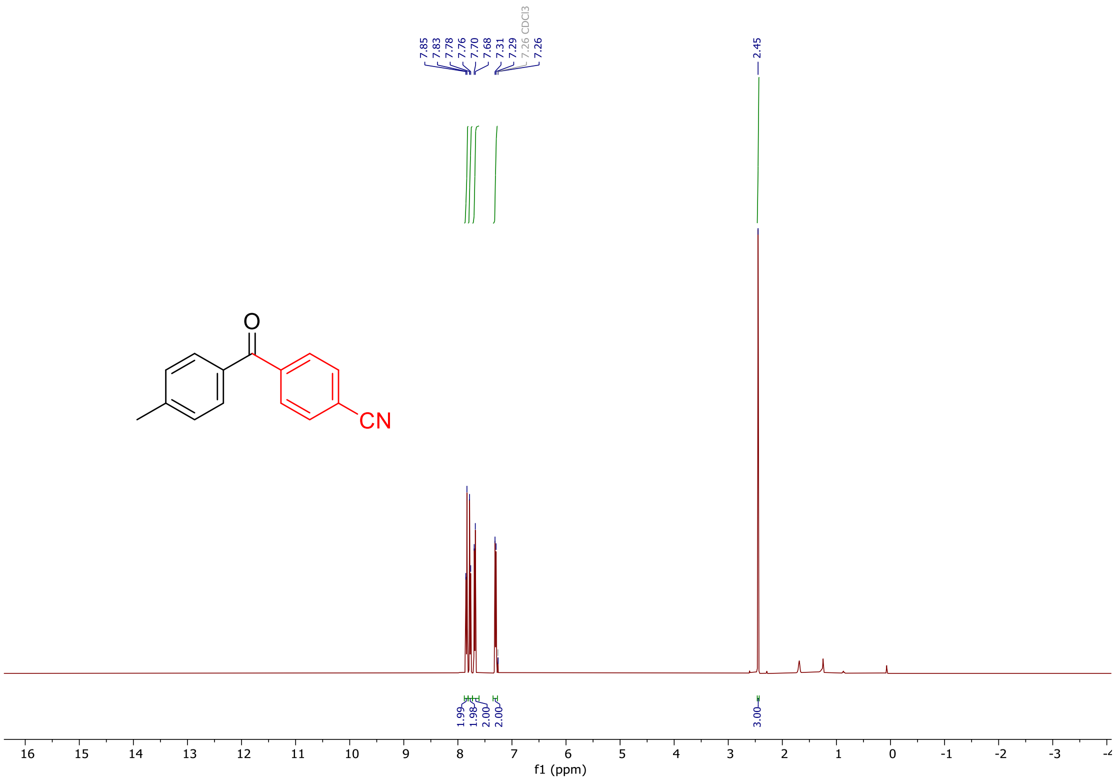

Figure S58. ^{1}H NMR of 4-(4-methylbenzoyl)benzonitrile

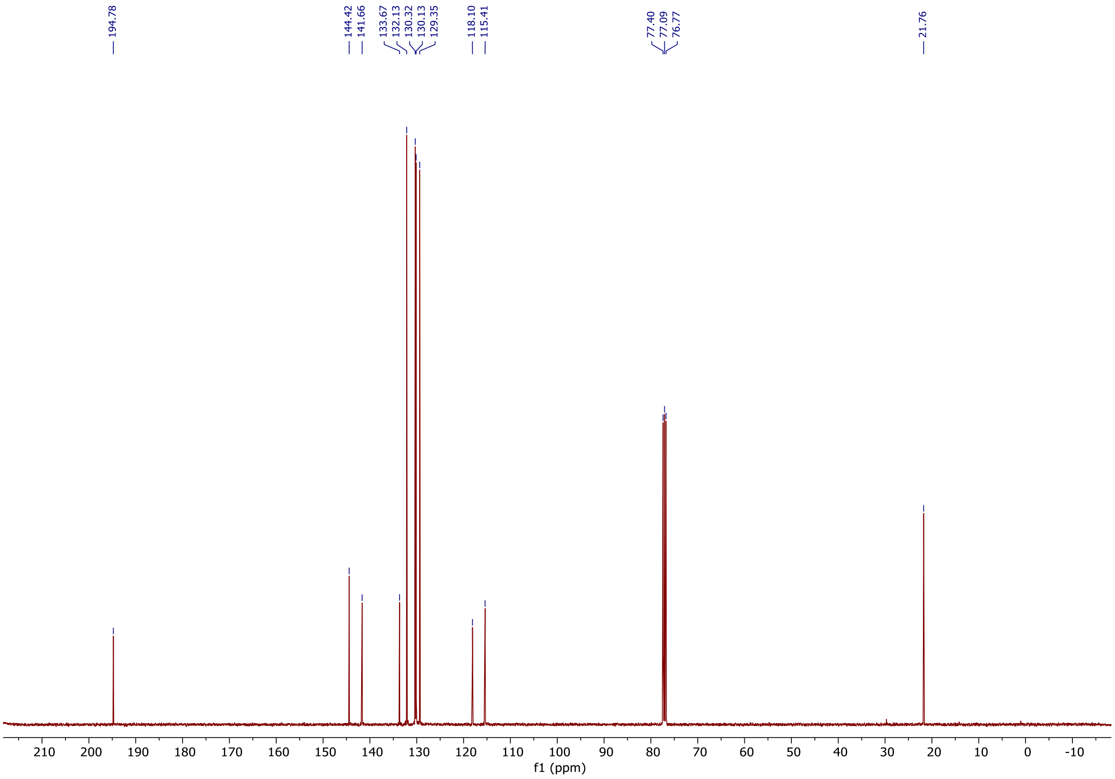

Figure S59. ^{13}C NMR of 4-(4-methylbenzoyl)benzonitrile

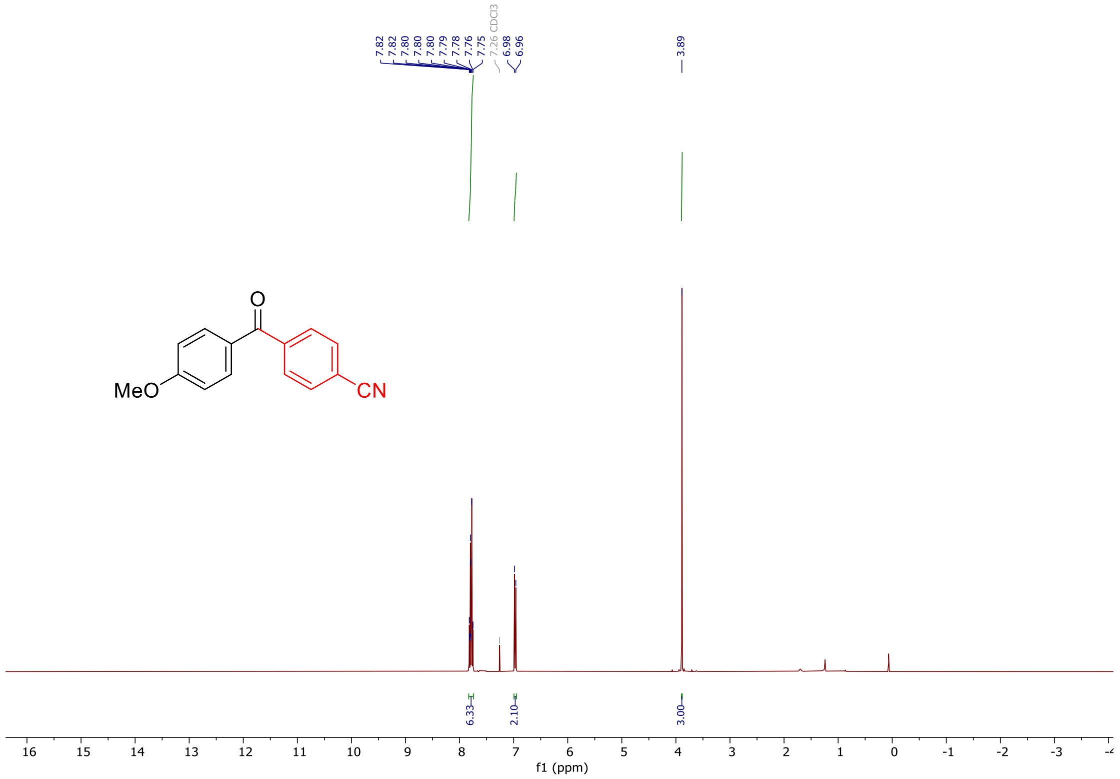

Figure S60. ^{1}H NMR of 4-(4-methoxybenzoyl)benzonitrile

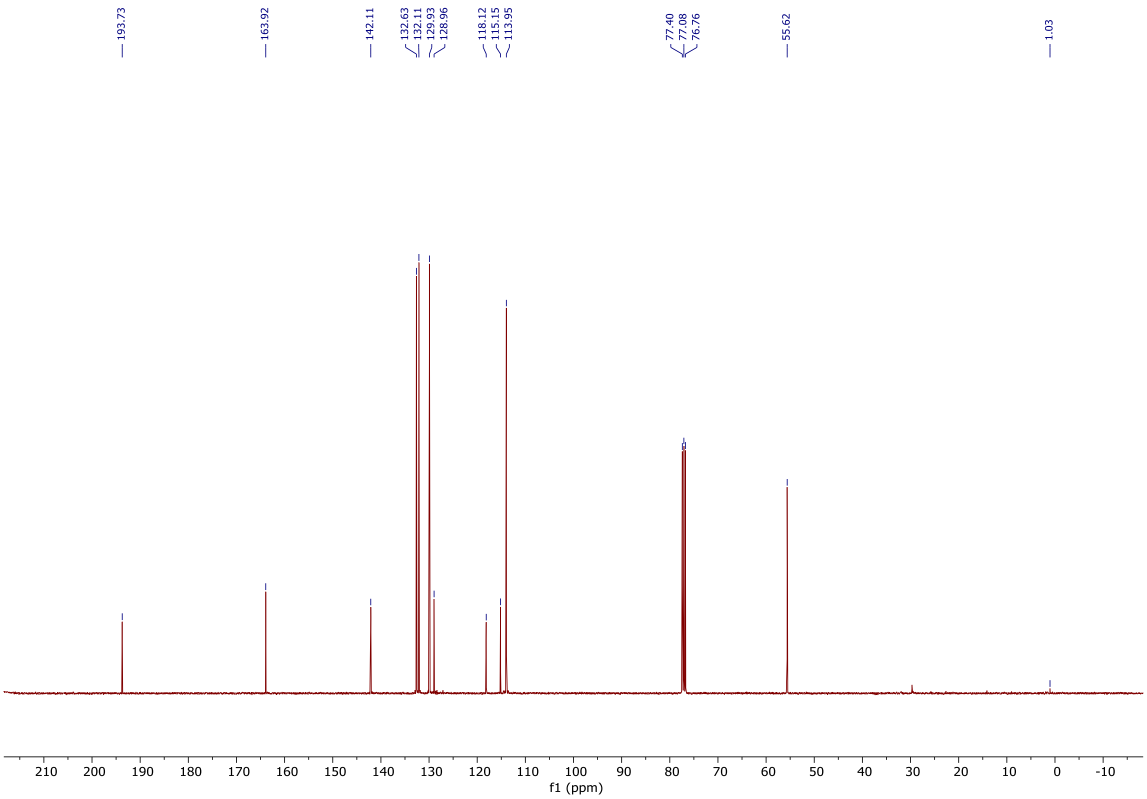

Figure S61. ^{13}C NMR of 4-(4-methoxybenzoyl)benzonitrile

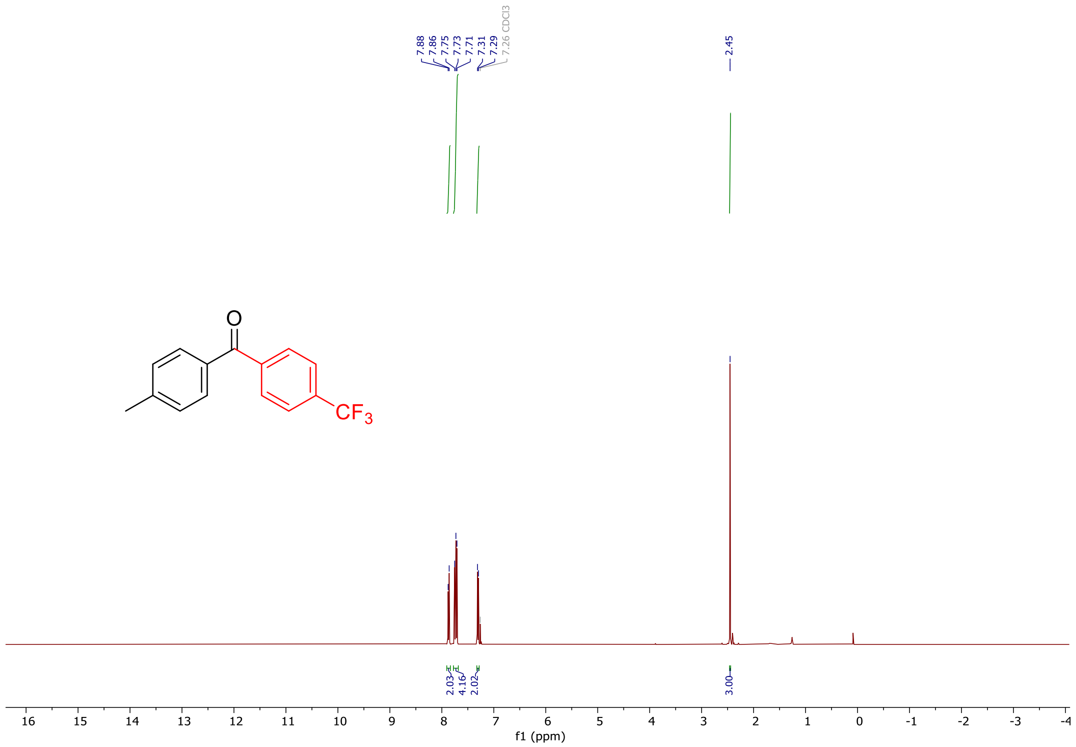

Figure S62. ^{1}H NMR of p-tolyl(4-(trifluoromethyl)phenylmethanone

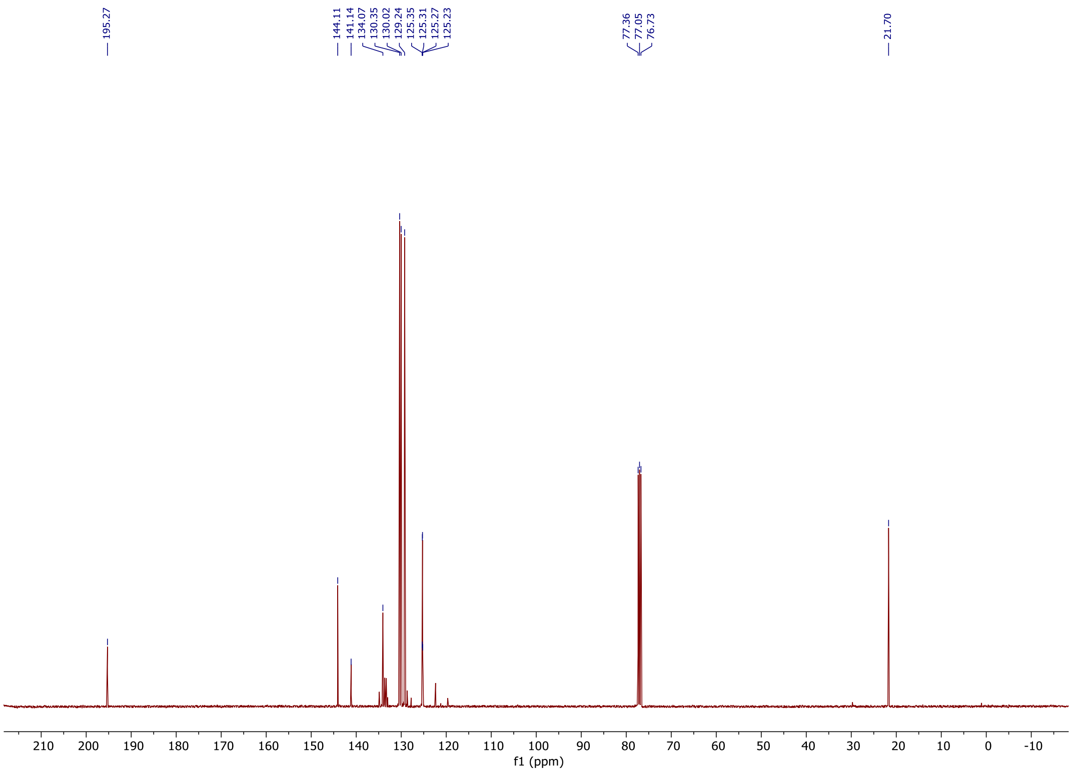

Figure S63. ^{13}C NMR of p-tolyl(4-(trifluoromethyl)phenylmethanone

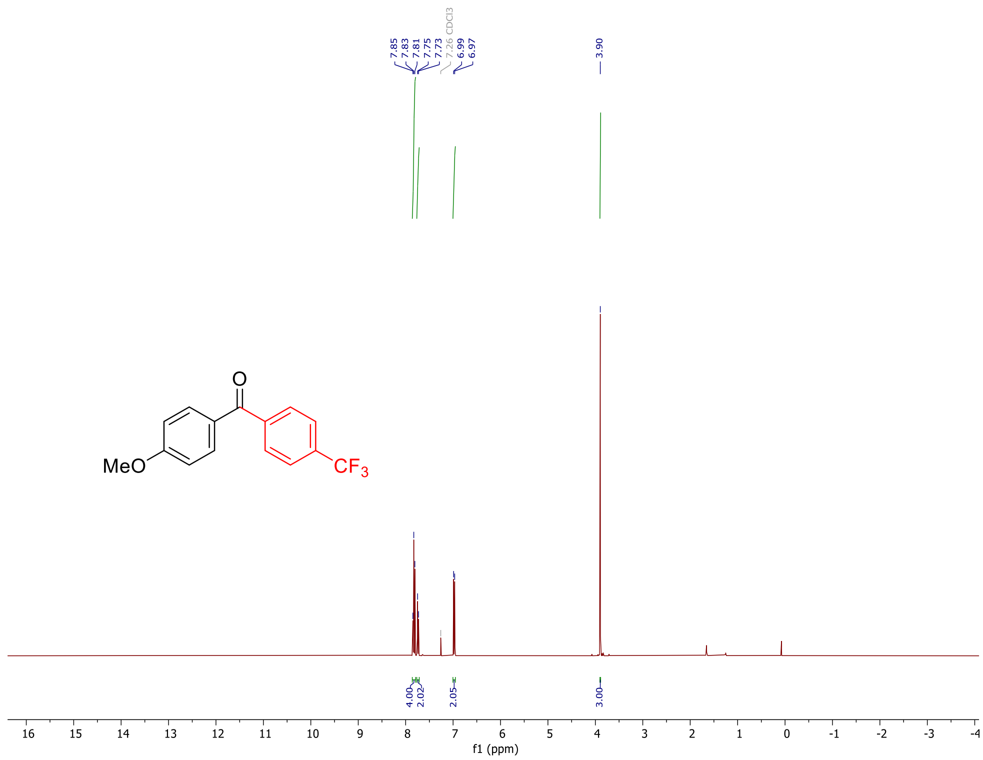

Figure S64. ^{1}H NMR of 4-methoxyphenyl(4-(trifluoromethyl)phenyl)mathanone

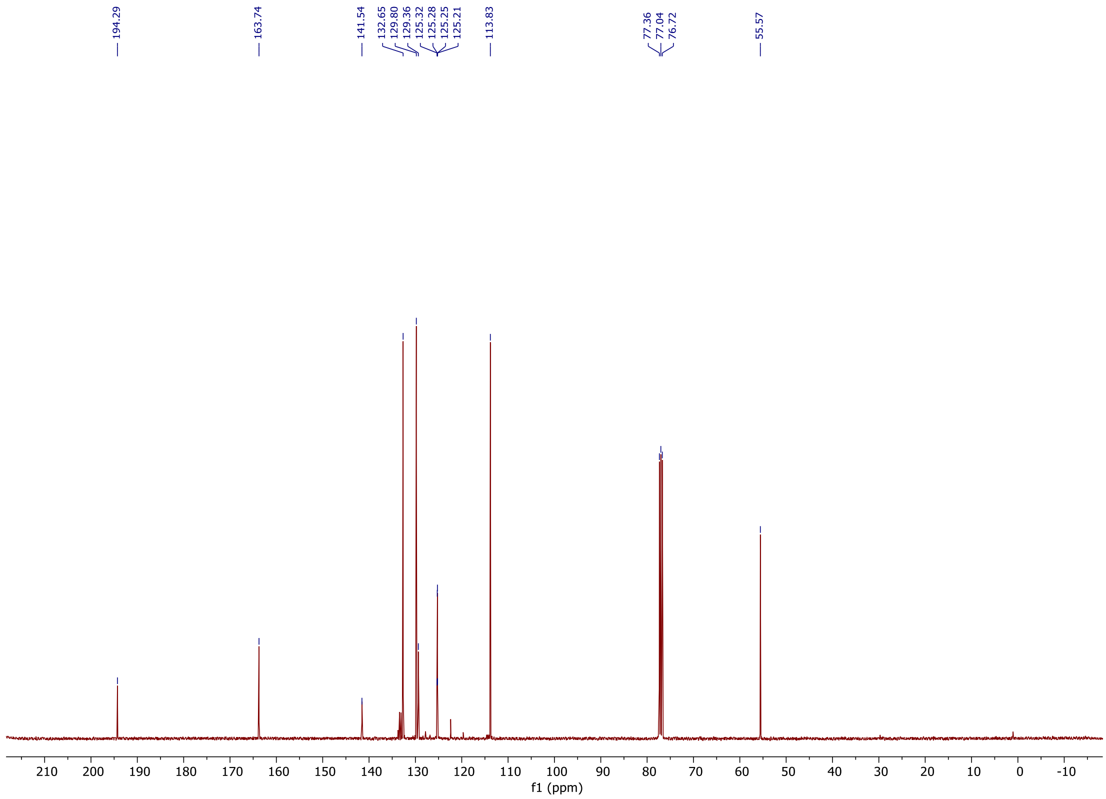

Figure S65. ^{13}C NMR of 4-methoxyphenyl(4-(trifluoromethyl)phenyl)mathanone

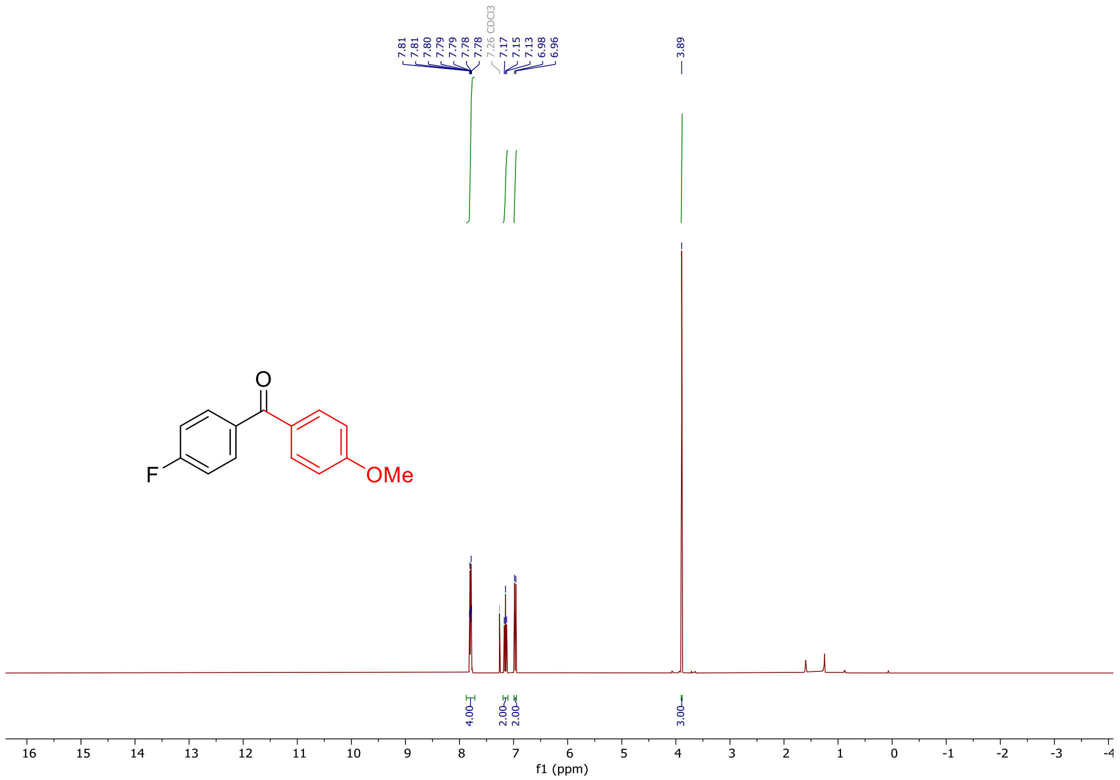

Figure S66. ^{1}H NMR of 4-fluorophenyl(4-methoxyphenyl)methanone

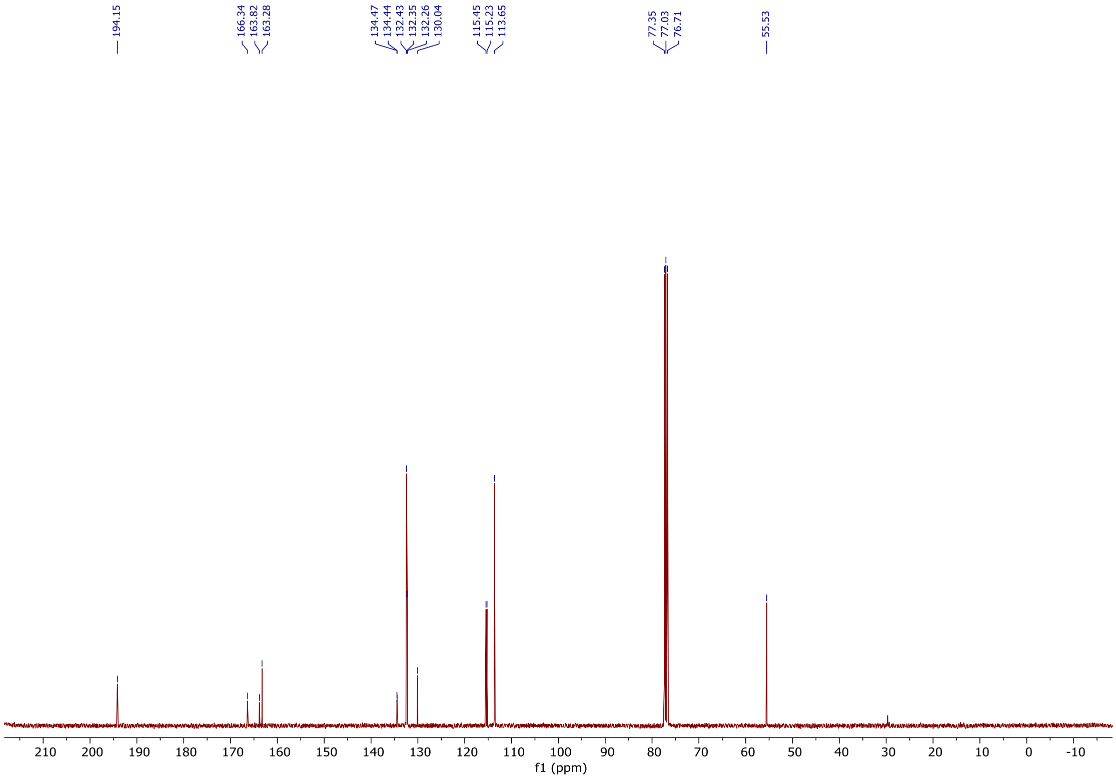

Figure S67. ^{13}C NMR of 4-fluorophenyl(4-methoxyphenyl)methanone

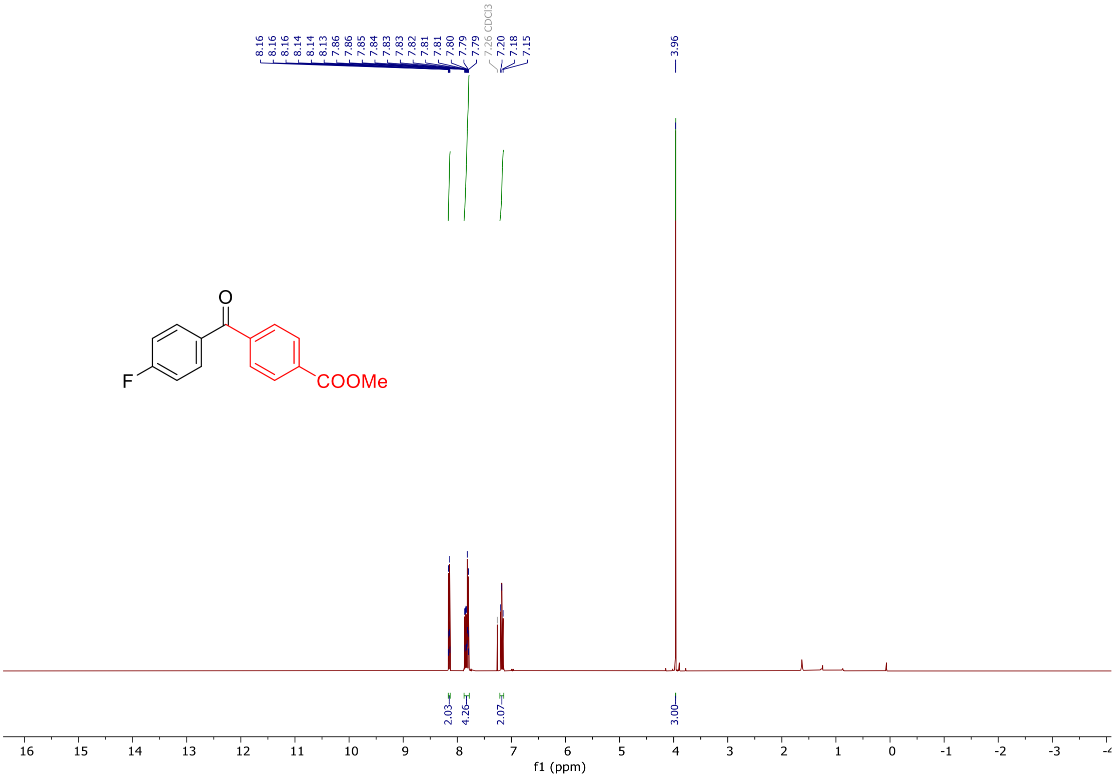

Figure S68. ^{1}H NMR of methyl 4-(4-fluorobenzoyl)benzoate

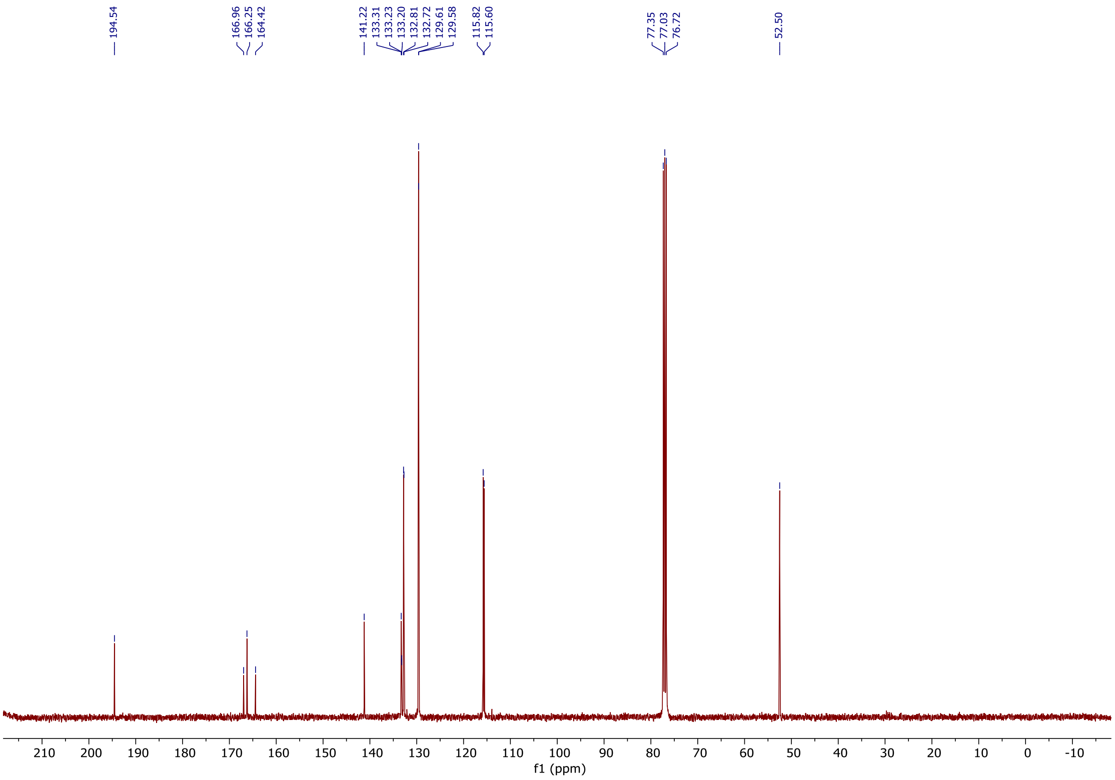

Figure S69. ^{13}C NMR of methyl 4-(4-fluorobenzoyl)benzoate
